# Ultrasonographic Evidence of Synovitis Correlates with Synovial Citrate and TBARS in Equine Osteoarthritis

**DOI:** 10.3390/vetsci13020140

**Published:** 2026-01-31

**Authors:** Anna Paula Barreira, Thaís Moreira, Rafaela Silva, Letícia Nunes, Adriana Lioi, Elizabeth Kraus, Vittoria Altheman, Marcela Ribeiro, Carla Leite, Andreza Silva, Fernando Almeida, Gilson Santos Junior, Daniel Lessa, Ana Liz Alves

**Affiliations:** 1 Department of Veterinary Medicine and Surgery, Veterinary Institute, Rural Federal University of Rio de Janeiro (UFRRJ), Seropedica 23890-000, Brazil; 2 Postgraduate Program in Animal Biotechnology, Regenerative Medicine Lab, School of Veterinary Medicine and Animal Science, São Paulo State University (UNESP), Botucatu 18618-970, Brazilana.liz@unesp.br (A.L.A.); 3 Veterinary Institute, Rural Federal University of Rio de Janeiro (UFRRJ), Seropedica 23890-000, Brazil; 4 Brazilian Jockey Club (JCB), Rio de Janeiro 22470-060, Brazil; 5 Postgraduate Program in Veterinary Medicine, Rural Federal University of Rio de Janeiro (UFRRJ), Seropedica 23890-000, Brazil; 6 Laboratory of Metabolomics (LabMet), Department of Genetics, State University of Rio de Janeiro (UERJ), Rio de Janeiro 20550-013, Brazil; 7 Department of Pathology and Veterinary Clinic, Federal Fluminense University, Niteroi 24230-340, Brazil

**Keywords:** metabolomics, oxidative stress, joint disease, horses

## Abstract

Osteoarthritis (OA) is a degenerative joint disease that affects humans and animals worldwide. Its early diagnosis remains challenging due to subtle clinical signs and late radiographic changes. This study aimed to explore candidate biomarkers associated with spontaneous OA and to investigate their correlation with ultrasonographic scores to support early diagnosis. Clinical, radiographic, and ultrasonographic evaluations were performed on 52 equine metacarpophalangeal joints, with and without OA, allowing joint scoring and classification into osteoarthritis (OAG) and control groups. Synovial fluid samples were analyzed for cartilage degradation (C2C), untargeted ^1^H NMR-based metabolomics, and lipid peroxidation (TBARS). Statistical analyses included Student’s *t*-test, Mann–Whitney U test, univariate and multivariate metabolomic analyses, and Spearman’s correlation (*p* < 0.05). Ultrasonography revealed higher scores in the synovial fold, membrane, and fluid, indicating synovitis as the predominant finding in the acute phase. C2C and TBARS concentrations were significantly higher in the OAG. Seven metabolites differed between groups, with citrate and TBARS showing the strongest correlations with ultrasonographic scores. These findings suggest increased metabolic activity and lipid peroxidation in early OA and highlight citrate and TBARS as potential auxiliary biomarkers for early diagnosis associated with synovitis.

## Simple Summary

Osteoarthritis (OA) is a joint disease in horses that causes pain and reduced mobility, but it is difficult to detect in its early stages. Since ultrasound can detect earlier disease changes than radiography, this study evaluated whether substances present in the synovial fluid, which lubricates the joints, could help identify OA based on their concentrations and their relationship with ultrasonographic findings. The metacarpophalangeal joints of 26 horses, with and without OA, were examined using radiography and ultrasound. Synovial fluid samples were analyzed for markers related to inflammation, cartilage damage, and oxidative stress. Joints affected by OA showed higher levels of citrate and oxidative stress markers compared to healthy joints, and these levels were associated with more severe ultrasound changes. These results suggest that citrate and oxidative stress markers reflect early joint inflammation and metabolic changes, highlighting their potential as supportive tools for the early diagnosis of osteoarthritis in horses alongside ultrasonography.

## 1. Introduction

Osteoarthritis (OA) is a highly prevalent joint disorder in both humans and animals, leading to progressive pain and disability [1,2,3]. Reliable biomarkers for early diagnosis and monitoring are still lacking [1,3], and identifying the disease at an early stage remains difficult as it often precedes clinical signs. OA affects several joint structures, including cartilage, subchondral bone, synovial membrane, joint capsule, ligaments, menisci, and the infrapatellar fat pad [4,5]. An imbalance between anabolic and catabolic processes results in degradation of proteoglycans and extracellular matrix components [6], progressing to fibrillation, fissures, and chondral fragments that may induce synovitis [5]. Synovitis is characterized by synovial lining hyperplasia, lymphocyte infiltration, and activated macrophages releasing pro-inflammatory cytokines and catabolic enzymes (e.g., aggrecanases and metalloproteinases) that exacerbate tissue breakdown and alter synovial fluid composition [5,7]. Although this pathological cycle occurs in most joint diseases, differences in cell density, gene expression, and mechanical loading among joints underscore the need for joint-specific therapeutic approaches [7].

Radiology is frequently used in the diagnosis of osteoarthritis despite its limited sensitivity, making it better for detecting bone changes that appear only in the late phase of osteoarthritis [8,9]. Therefore, detecting changes at an early stage requires combining other imaging techniques (ultrasonography and MRI), biomarkers (systemic and local), and clinical manifestations (pain, swelling, and morning stiffness) [6,8,9,10,11]. Radiographic signs of osteoarthritis mostly include subchondral bone sclerosis, osteophytes/enthesophytes, and joint space changes [9]. A combination of radiography and ultrasonography is the most common approach in veterinary clinical practice for an early diagnosis [12]. Ultrasonographic findings of osteoarthritis include effusion, thickening of the synovial membrane, fibrous capsule, enthesopathies, and irregularity of the cartilage and bone surface [4,12].

Reliable biomarkers for early-stage osteoarthritis remain highly sought after, including genetic markers [7] and those related to cartilage turnover and synovial activity [1,6,8]. However, none have demonstrated sufficient diagnostic accuracy, accessibility, and clinical validation for routine application [6]. Current protein-based candidates include direct markers of cartilage metabolism (CP-II, C2C, and CS-846) and synovial activation (HA), as well as indirect indicators of inflammation such as cytokines, metalloproteinases, eicosanoids, growth factors, and C-reactive protein. Specifically, CP-II reflects type II collagen synthesis, C2C indicates its degradation, CS-846 is associated with aggrecan synthesis, and hyaluronan (HA) contributes to synovial fluid viscoelasticity and extracellular matrix integrity [1,6,8,13].

Metabolomics is an emerging approach for synovial fluid evaluation that investigates biological mechanisms through metabolite profiling in fluids, cells, and tissues [3]. It can be performed using mass spectrometry or high-resolution nuclear magnetic resonance spectroscopy [14]. Proton nuclear magnetic resonance (^1^H NMR) provides a highly reproducible assessment of both global and targeted metabolic changes [6,15], enabling the simultaneous detection of multiple metabolites, including low-molecular-weight compounds directly affected by inflammatory processes [16]. In horses, ^1^H NMR distinguished septic from non-septic arthritis, identifying glucose as a key discriminator, while 14 other metabolites, including citrate, alanine, creatinine, glutamine, glycine, pyruvate, and valine, were increased in non-septic conditions [15]. Metabolomic studies in equine osteoarthritis have identified significant alterations in up to 19 metabolites, with elevated levels of choline, amino acids, nucleotides, creatine derivatives, and organic acids in affected joints [16]. In dogs, ^1^H NMR detected five discriminatory metabolites—mannose, betaine, 2-hydroxyisobutyrate, isoleucine, and lactate—supporting its potential as a biomarker-based strategy for monitoring OA progression [17].

Due to the avascular nature of articular cartilage and its low-oxygen microenvironment, oxidative stress has been proposed as an additional biochemical marker of osteoarthritis [18]. Reactive oxygen species (ROS), particularly superoxide anions and hydroxyl radicals, degrade collagen, proteoglycans, and hyaluronan, contributing to cartilage damage [19,20]. Although oxidants are normally involved in signaling, repair, and antioxidant regulation, their excessive production or impaired neutralization leads to oxidative stress, which upregulates inflammatory pathways and accelerates joint tissue degradation [1,20,21]. Lipid peroxidation of polyunsaturated fatty acids generates malondialdehyde (MDA), quantifiable by the Thiobarbituric Acid Reactive Substances (TBARS) assay [19,21]. In equine joint disease, increased oxidative stress has been associated with enhanced neutrophil infiltration and nitric oxide and myeloperoxidase release during acute inflammation, alongside elevated TBARS levels in synovial fluid [19,22].

Therefore, this study aimed to explore candidate biomarkers associated with spontaneous osteoarthritis and to evaluate their correlations with ultrasonographic features as these changes precede radiographic findings in osteoarthritis.

## 2. Materials and Methods

### 2.1. Ethical Approval

This study was carried out under the approval of the Ethical Committee on the Use of Animals in Research (CEUA/IV, UFRRJ; protocol no. 4490260819) that is registered in the Brazilian National Council for Animal Control and Experimentation (CONCEA; CIAEP no. 01.0115.2014, 6 May 2014).

### 2.2. Study Design and Experimental Animals

Twenty-six horses participated in the study. The osteoarthritis group (OAG) consisted of 20 horses with spontaneous osteoarthritis (five females and 15 males), aged 3–22 years, housed in training centers. The control group (CG) consisted of six horses without metacarpophalangeal joint injury (four females and two males), aged 3–5 years, housed at the university farm.

The horses in the OAG were assigned by referring veterinarians to the study based on previous diagnosis of osteoarthritis, confirmed by clinical, radiographic, and ultrasonographic exams. The CG consisted of young horses without exercise routine or abnormality in clinical and imaging examinations.

This was a cross-sectional, observational, and clinical study with two groups: osteoarthritis and control. The horses underwent clinical, radiographic, and ultrasound examinations of the metacarpophalangeal joints, with scores assigned. Synovial fluid was submitted initially to physical, chemical, and cytological evaluation. Then, C2C quantification was performed to assess cartilage degradation, TBARS analysis was conducted to evaluate lipid peroxidation, and untargeted ^1^H NMR spectroscopy was employed to characterize the metabolomic profile of each joint.

Data were first tested for normality using the Kolmogorov–Smirnov test. Descriptive statistics, Student’s *t*-test, and the Mann–Whitney U test were applied for group comparisons, as appropriate. Univariate and multivariate analyses were used to assess metabolomic profiles and to assess the influence of clinical and exercise-related variables on synovial fluid biomarkers. Spearman’s rank correlation was performed to evaluate the relationship between imaging scores and potential biomarkers. Statistical significance was set at *p* < 0.05.

### 2.3. Orthopedic Evaluation

Orthopedic exams of both metacarpophalangeal joints were scored in consensus by two certificated evaluators. The score system was based on a Likert scale (0—absent; 1—mild; 2—moderate; and 3—severe) for increase in joint size, joint distention (effusion), heat, and pain on palpation. Lameness analysis was done at walk and at trot on a straight line with and without fetlock flexion, alternating the limbs. The scoring was based on the American Association Equine Practitioner (0–5 scale) [23].

### 2.4. Radiographic Evaluation

Radiography of metacarpophalangeal joints was performed with an Ecoray Orange portable X-ray unit, model 1060 HF Mobile (ECORAY Co. Ltd., Seongdong-gu, Seoul, Republic of Korea) with power set at 74 kV and 2.0 mAs. The film focus distance was 80 cm. Five projections were captured: lateromedial, dorsopalmar, dorsolateral-palmaromedial 45o oblique, dorsomedial-palmarolateral 45o oblique, and flexed lateromedial. Images were stored in a digital system for evaluation by two evaluators in consensus (Equarter Academy diplomate). The inclusion criterion was the confirmation of previous diagnosis of osteoarthritis in at least one metacarpophalangeal joint in any degree or phase of evolution.

A numerical scoring system described in the literature [24] was applied to each joint, yielding a score ranging from 0 to 24 (Table 1).

### 2.5. Ultrasonographic Evaluation

Ultrasound evaluation was performed with a Sonosite M-Turbo device (Fujifilm SonoSite Inc., Bothel, WA, USA) and linear transducer (6–12 MHz) in longitudinal and transverse planes. Eight intra-articular structures were evaluated, such as synovial membrane and fold, bone surface, medial and lateral collateral ligaments (short and long), and the appearance of the synovial fluid, according to the described protocol [25].

Measurements were expressed in area (mm^2^) or thickness (mm) depending on the structure under analysis. The parameters evaluated were size, echogenicity, echotexture, shape, fiber orientation, and presence of osteochondral fragment or mineralization, based on previously established criteria [26]. A binary scoring system (0—normal; 1—abnormal) was applied to each joint, resulting in scores ranging from 0 to 42 (Table 2).

### 2.6. Group Classification Criteria

The sum of the radiographic and ultrasonographic scores generated a final score (0–66). The inclusion criterion for the OAG was the presence of sum of radiographic and ultrasonographic scores above 5, while for inclusion in the CG, it was scores of up to 4.

### 2.7. Synovial Fluid Collection, Transport, and Basic Analyses

Metacarpophalangeal arthrocentesis of both forelimbs was performed from dorsolateral pouch with surgical antisepsis. The horse was kept in a stable position with physical restraint, but if necessary, it was sedated with 1% detomidine hydrochloride (20 mcg/kg/IV). With a flexed fetlock, centesis was performed with a 30 mm long 0.7 mm needle and a 5 mL syringe. Synovial fluid was collected in tubes with and without EDTA.

Synovial fluid was initially subjected to physical, chemical, and cytological evaluation. The remaining samples were centrifuged at 720× *g* for 10 min, aliquoted into three 1.5 mL polypropylene tubes, and stored at −20 °C for advanced analyses. Enzyme-linked immunosorbent assay (ELISA) was performed to quantify a cartilage degradation biomarker (C2C), spectrophotometry was used to determine thiobarbituric acid-reactive substances (TBARS) as an indicator of lipid peroxidation, and untargeted ^1^H NMR spectroscopy was employed to identify the metabolomic profile of each joint.

### 2.8. Collagen Metabolism Analysis

At ambient temperature, synovial fluid samples were submitted to a multispecies ELISA for measuring the concentration of C2C (IBEX Pharmaceuticals, Inc., Montreal, QC, Canada) following the manufacturers’ instructions. Optic densities were read at 450 nm wavelengths, and the regression curve was performed and evaluated using Instat GraphPadTM software (GraphPad Prism version 9.3.0). The technique uses polyclonal and monoclonal antibodies, activating immune-specific responses against joint fragments, according to the methodology disclosed by the manufacturer.

### 2.9. Global ^1^H NMR-Based Metabolomics

This procedure was performed according to a previous study [27]. Briefly, Amicon Ultra-2 tubes/3KD mesh (#UFC200324, Merck Millipore, Darmstadt, Germany) were pre-rinsed with Milli Q water and subjected to four centrifugations at 4000× *g* for 20 min at room temperature to eliminate interference from large molecules. To homogenize the pH and viscosity of the samples, 500 μL of supernatant was mixed with 1000 μL of 50 mM sodium phosphate buffer at pH 7.4, 10% deuterated water (D2O), and 0.1 mM of 2,2-dimethyl-silapentane-2,5-sulfonate (DSS), which served as the lock. The mixture was filtered on Amicon Ultra according to the manufacturer’s recommendations. After that, samples were kept at −20 °C until analysis.

Once at room temperature, the samples were homogenized, placed in an NMR tube, and subjected to hydrogen proton high-resolution ^1^H NMR spectroscopy. NMR spectra from synovial fluid samples were acquired at 500.13 MHz (Bruker Avance III DRX 500 MHz spectrometer, Bruker BioSpin/Bruker Co., Billerica, MA, USA) at an ambient temperature of 298 K. The pulse used was zgESGP, with 64 K points, 512 scans, a spectral width of 19.99 ppm, an acquisition time of 3.27 s, a transmitter offset of 4.704 ppm, a receive gain of 203, and baseopt digitization mode. After acquisition, spectra were uploaded to MestReNova software version 14.2.1-27684 and zero-filled with 128 K, with phase and baseline corrected and calibrated using DSS. In the same software, the binning table was generated with a 0.02 ppm interval using the average sum method. Data were normalized using DSS, and ethanol, water, and other DSS signals were removed. Principal component analysis (PCA) was performed using Metaboanalyst 5.0 [28].

### 2.10. TBARS Analysis

The quantification of lipoperoxidation was performed according to the protocol previously described in [29], which was based on the production of thiobarbituric acid (TBARS) quantified by spectrophotometry. Aliquots at an amount of 500 μL of the samples were added to 1000 μL of 10% trichloroacetic acid solution and centrifuged at 1800× *g* for 15 min at 15 °C for protein precipitation. Aliquots at an amount of 500 μL of the supernatant were placed in tubes along with 500 μL of 1% thiobarbituric acid and dissolved in freshly prepared 0.05 N sodium hydroxide. The samples were then incubated in a boiling water bath at 100 °C for 10 min and cooled in an ice bath at 0 °C. TBARS were quantified in a spectrophotometer at a length of 532 nanometers (nm). Reactions occur at a temperature between 90 °C and 100 °C at acidic pH.

### 2.11. Statistical Analysis

Descriptive analysis, Student’s *t*-test, and Mann–Whitney U test were conducted to compare groups. A Smirnov–Kolmogorov test was performed to check the normality of the data. Student’s *t*-test was used for variables that met a normal distribution, and the Mann–Whitney U test for nonparametric variables for parametric and nonparametric tests (SAS packages, https://welcome.oda.sas.com, accessed on 24 September 2024). Statistical significance was considered at *p* < 0.05. Univariate and multivariate analyses were performed to identify metabolites in synovial OAG samples, to compare group profiles, and to assess the influence of clinical and exercise-related variables on synovial fluid biomarkers.

Spearman correlation was calculated between imaging scores and biomarkers only for the OA group (GraphPad Prism version 9.3.0/Metaboanalyst 5.0). Coefficient interpretation was done according to a previously described protocol [30], considering r = 0.00–0.10 as a negligible correlation, r = 0.10–0.39 as a weak correlation, r = 0.40–0.69 as a moderate correlation, r = 0.70–0.89 as a strong correlation, and r = 0.90–1.00 as a very strong correlation.

## 3. Results

### 3.1. Experimental Animals Profile

There was a significant difference between groups regarding age and body weight. Animals from the CG presented lower mean age (3.55 years) and body weight (332.0 kg) than those from the OAG (9.14 years and 466.7 kg, respectively) (Table 3). Also, the CG had no exercise routine, while animals from the OAG were sport horses.

### 3.2. Orthopedic Results

No significant differences in clinical manifestations were observed between groups, suggesting that the clinical signs of OA were subtle (Table 3). The highest mean score was recorded for lameness after the flexion test (1.14 ± 1.16/Supplementary Data), reinforcing the importance of including this assessment in routine lameness evaluations.

### 3.3. Radiographic Scoring

**Figure 1:**
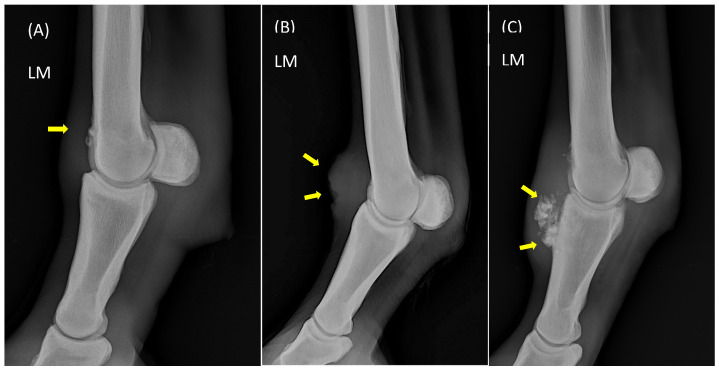
Lateromedial (LM) radiographs of metacarpophalangeal joints from different horses in this study showing signs of joint disease. (**A**) Medium-sized entheseophytes on the dorsal aspect of the third metacarpal bone at the site of capsular insertion (arrow); (**B**) soft tissue thickening (arrows); and (**C**) mineralization of the dorsal aspect of the synovial membrane/capsule (upper arrow), associated with osteoproliferation of the proximal phalanx (lower arrow).

A significant difference was observed between groups in the radiographic and ultrasonographic scores, as expected. Detailed radiographic analysis revealed that the highest mean score corresponded to soft tissue thickening (1.62 ± 1.10), followed by size (1.50 ± 1.18) and number (1.45 ± 1.15) of osteophytes (Supplementary Data) (Figure 1), emphasizing the importance of ultrasonographic examination.

### 3.4. Ultrasonographic Scoring

**Figure 2:**
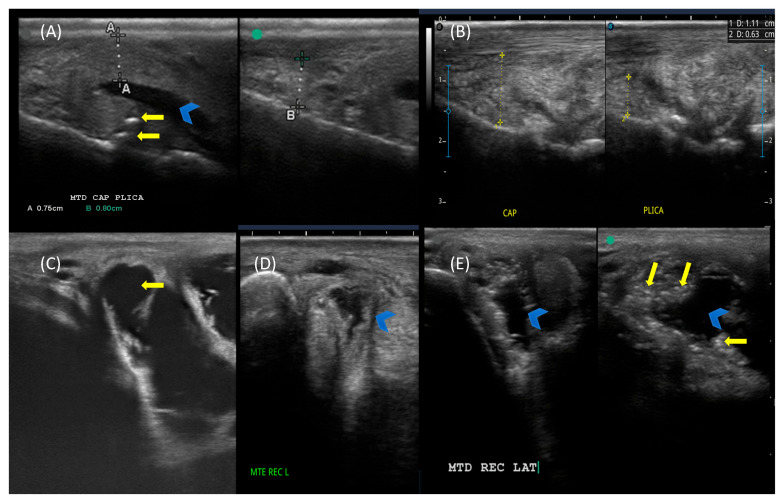
Ultrasonographic scans of metacarpophalangeal joints from different horses showing synovitis. Dorsal longitudinal scans showing (**A**) hypoechoic capsular thickening (A + 0.75 cm), focal mineralization (yellow arrows), effusion (blue arrowhead), and heterogeneous synovial plica (B + 0.80 cm) and (**B**) hyperechoic, heterogeneous capsular thickening (capsule + 1.11 cm; plica + 0.63 cm). Transverse scans of the dorsolateral joint recess showing (**C**) moderate effusion (yellow arrow); (**D**) moderate thickening with mild effusion (blue arrowhead); and (**E**) moderate effusion (blue arrowheads) with capsular thickening and multifocal mineralization (yellow arrows). Green dot demonstrates proximal position on longitudinal scan and lateral position on transverse scan.

The most prevalent ultrasonographic findings were moderate joint effusion and hypoechoic capsule thickening, consistent with acute synovitis, even though a few joints presented mineralization of the joint capsule. In the OAG, the highest mean score was recorded for the synovial plicae (2.75 ± 1.10), which showed a significant difference between groups (*p* = 0.001) (Figure 2).

### 3.5. Synovial Fluid Basic Results

Arthrocentesis was successfully performed in 49 out of 52 joints; however, obtaining sufficient synovial fluid volume for all laboratory analyses was challenging. For the first ten joint harvests, samples were transported on dry ice (−80 °C), as recommended in the literature; however, this procedure compromised cellular integrity. To preserve cellular morphology, subsequent samples were shipped under refrigeration (0 °C). Basic analyses were performed to confirm sample quality prior to advanced techniques.

Physicochemical analysis (CG n = 1/OAG n = 37) of the synovial fluid revealed significant differences between groups in volume, total protein, and pH, all of which were elevated in the OAG (Table 4). Cytological evaluation (CG n = 8/OAG n = 22) showed a predominance of large mononuclear cells in both groups; however, statistical differences were observed only for neutrophils and eosinophils. Interestingly, the mean total nucleated cell count (TNCC) and the percentages of neutrophils and eosinophils were higher in the CG compared to the OAG (Table 4).

### 3.6. Collagen Metabolism Results

With respect to collagen metabolism (CG n = 8/OAG n = 37), the markedly higher C2C concentration observed in the OAG compared to the CG (Supplementary Data) indicates enhanced cartilage degradation in the affected joints, as expected (Table 4).

### 3.7. Global ^1^H NMR-Based Metabolomics Results

**Figure 3:**
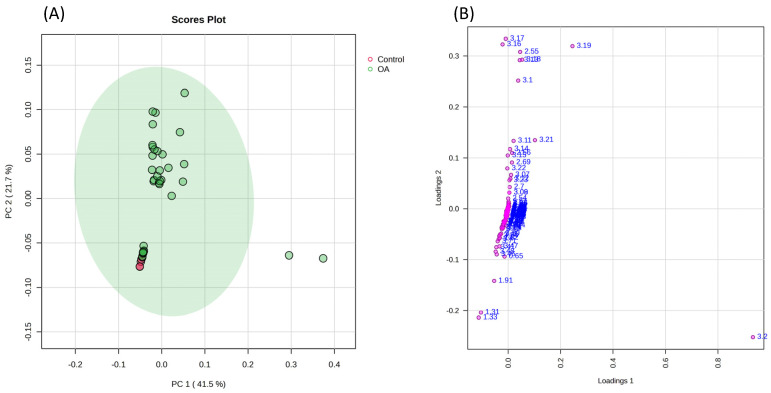
(**A**) Principal component analysis (PCA) score plot of control (red) and osteoarthritis (green) groups in equine synovial fluid samples, which did not indicate a clear difference between groups. Component 1 accounted for 41.5% of the total variance, while component 2 explained 21.7%. (**B**) PCA loading plot showing the distribution of buckets in loading 1, indicating that choline and citrate contributed the most to the principal component. Pink dots show higher magnitudes and contributed more strongly to group separation.

**Figure 4:**
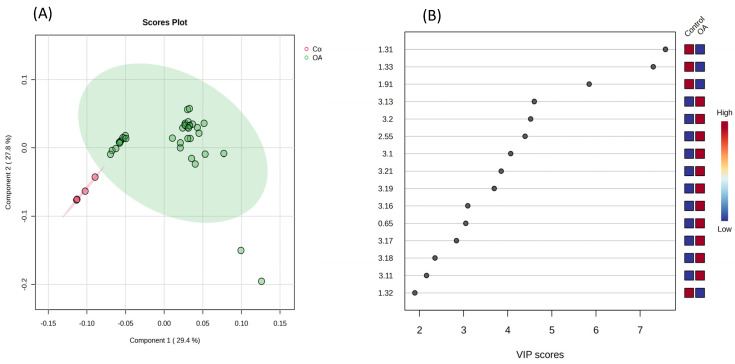
(**A**) Partial Least Squares Discriminant Analysis (PLS-DA) revealed differences between the OAG (green) and CG (red). (**B**) Variable Importance in Projection (VIP) scores were used to assess the contribution of each variable to the separation between groups, identifying citrate and choline as elevated in the osteoarthritis group, whereas lactate and acetate were elevated in the control group (red square).

Regarding the metabolomic analysis (CG n = 4/OAG n = 37), a data matrix comprising 336 buckets was obtained. Although the principal component analysis (PCA) did not indicate a clear difference between groups regarding synovial metabolites (Figure 3), the supervised Partial Least Squares Discriminant Analysis (PLS-DA) revealed a tendency toward separation (Figure 4A). In Variable Importance in Projection (VIP), samples from the osteoarthritis group showed a relative increase in the spectral signals of some metabolites compared to the control group, identifying them as the main discriminants (Figure 4B). These findings were supported by cross-validation tests (R^2^, Q^2^, and permutation) (Supplementary Data), which confirmed the robustness and reliability of the models in membrane metabolism, anaerobic glycolysis, and lipid profiles in the synovial fluid.

**Figure 5:**
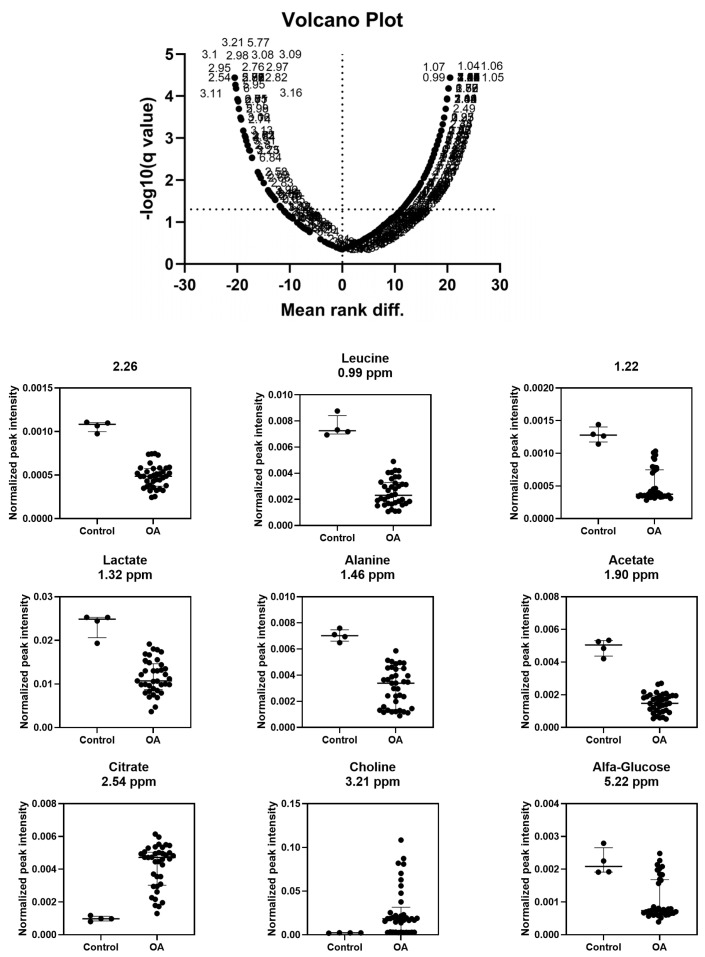
Univariate analysis of the metabolites that demonstrated the greatest significant difference between groups; according to the volcano plot, they were citrate (2.54 ppm; *p* = 0.00002), choline (3.21 ppm; *p* = 0.00002), leucine (0.99 ppm; *p* = 0.00002), lactate (1.32 ppm; *p* = 0.00002), alanine (1.46 ppm; *p* = 0.00002), α- glucose (5.22 ppm; *p* = 0.00476), and acetate (1.90 ppm; *p* = 0.00002), confirmed by univariate analysis.

According to the volcano plot, the regions that most contributed to the distinction between groups corresponded to seven metabolites: citrate (2.54 ppm/*p* = 0.00002), choline (3.21 ppm/*p* = 0.00002), leucine (0.99 ppm/*p* = 0.00002), lactate (1.32 ppm/*p* = 0.00002), alanine (1.46 ppm/*p* = 0.00002), α-glucose (5.22 ppm/*p* = 0.00476), and acetate (1.90 ppm/*p* = 0.00002) (Figure 5/Supplementary Files). In the OAG, compared with the CG, more intense bucket signals were observed for citrate and choline, whereas α-glucose, leucine, lactate, alanine, and citrate showed less intense signals.

### 3.8. TBARS Concentration 

The analysis of lipid peroxidation (CG n = 8/OAG n = 37) also revealed a significant difference between groups in TBARS concentrations, with mean values being higher in the osteoarthritis group compared to the control group (Supplementary Data) (Table 4).

### 3.9. Correlation Between Imaging Scores and Potential Biomarkers

**Figure 6:**
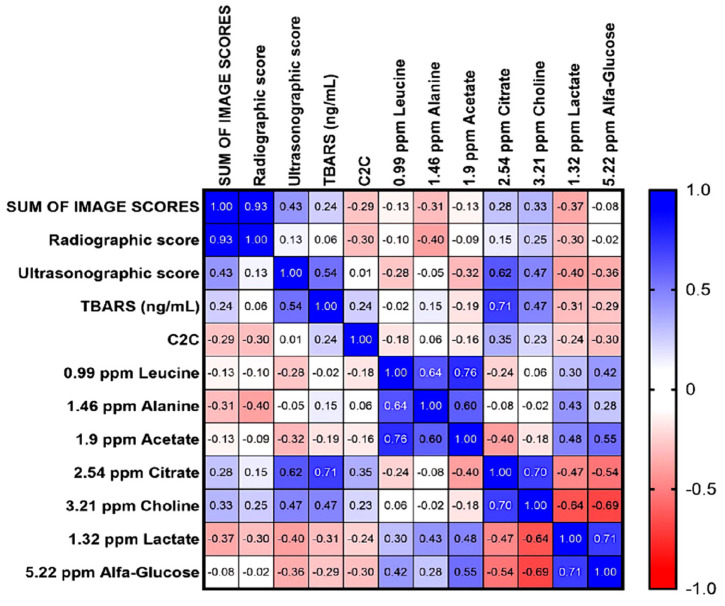
A clustered heatmap based on Spearman’s correlations (R) was generated between imaging scores and synovial biomarkers. The strongest correlations were observed between ultrasonographic scores and citrate and TBARS concentrations, whereas the highest correlation with radiographic scores was observed for alanine. Correlation coefficients (R value) are shown in each square.

Spearman’s correlation between imaging scores and biomarkers of the OA group demonstrated that radiographic scores showed a moderate negative correlation with alanine concentration (r = −0.40), suggesting that the inflammatory process tends to decrease in the presence of visible bone alterations on radiographs, which are characteristic of the late stage of the disease. Regarding ultrasound scores, the strongest correlations were observed with citrate (r = 0.62) and TBARS (r = 0.54) (Figure 6).

Together, these results reinforce the association between ultrasound findings, primarily represented by acute synovitis, and the concentrations of citrate and TBARS in spontaneous osteoarthritis, suggesting that these metabolites may serve as indicators of increased metabolic activity and oxidative damage in the early stages of the disease.

## 4. Discussion

### 4.1. Clinical Trial

Differences between groups in this study were a consequence of the strict inclusion criteria applied to the control group, which required the complete absence of clinical, radiographic, and ultrasonographic signs of osteoarthritis. Although previous studies have demonstrated that established risk factors for osteoarthritis, including age, body weight, and athletic activity, may influence the joint environment and synovial homeostasis [5,31], the PCA and PLS-DA results (Supplementary Data) support the presence of consistent multivariate differences among the analyzed groups while indicating a similar behavior of covariates such as age, body weight, and equestrian modality across imaging score categories.

Subtle clinical signs observed in our study reinforce the challenge of detecting early stages of osteoarthritis, as previously reported in the literature [5].

### 4.2. Radiographic Findings

Radiographic findings such as osteophytes and enthesophytes were frequently observed; however, these typically develop during later stages of OA, over 40 days after the onset of the initial inflammatory process, according to studies that experimentally induced OA in equine metacarpophalangeal joints [10]. The highest radiographic score was related to increased radiopacity and soft tissue thickening, which require the use of complementary imaging modalities such as ultrasonography or magnetic resonance imaging [9,11].

### 4.3. Ultrasonographic Findings

On ultrasonographic examination, soft tissue inflammation was predominantly characterized by joint effusion and hypoechoic thickening of the joint capsule, particularly involving the plicae. These ultrasonographic features are consistent with acute synovitis. According to the literature, acute synovitis is characterized by synovial membrane thickening, prominence of synovial folds, villous edema, and synovial fluid effusion [31,32].

According to the literature [33], acute synovitis is common in horses and represents a primary cause of joint pain even when radiographic abnormalities are absent. In the present study, signs of acute synovitis were prevalent, but chronic synovitis was also identified, as evidenced by mineralization of the joint capsule. This condition may result from repeated trauma, osteochondral fragmentation, or both, potentially progressing to dystrophic mineralization and osteoarthritis [34].

### 4.4. Synovial Sample Handling and Basic Analyses

The absence of synovial fluid retrieval in three out of 52 joints was attributed to chronic synovitis, particularly when the synovial plica was affected, leading to fibrosis and reduced synovial production [10]. In contrast, effusion was the most frequent finding, associated with acute synovitis. The synovial membrane is composed of macrophages, fibroblast-like cells, and fenestrated capillaries, separated by a matrix rich in collagen and glycosaminoglycans. During acute inflammation, vasodilatation and cell migration increase fluid and protein content, while enzymes such as hyaluronidase and metalloproteinases degrade hyaluronan, increasing fluid volume and reducing viscosity [33,35]. Conversely, lower cellularity in the osteoarthritis group compared with controls was attributed to the use of dry ice during sample transport, which likely caused cell lysis.

### 4.5. Collagen Metabolism Biomarker

Higher C2C concentrations measured by ELISA were observed in the osteoarthritis group, reflecting cartilage degradation in the affected joints, as expected. The relevance of extracellular matrix turnover biomarkers has been increasingly recognized over the past decade [6]. However, the clinical application of this technique remains limited by its high cost and the requirement to analyze large sample sets due to the short shelf life of test reagents.

### 4.6. Global Metabolomics

The metabolomic analysis revealed a higher spectral bucket intensity for citrate and choline in the osteoarthritis group. Among the detected metabolites, citrate exhibited the strongest correlations with ultrasonographic scores and TBARS concentrations, suggesting a close association with acute soft tissue alterations and oxidative damage. The acute phase of osteoarthritis is characterized by increased and heterogeneous synovial plicae, as well as thickening of the joint capsule [32], which were the main ultrasonographic findings observed in the present study.

The increased citrate signal detected in the osteoarthritis group may reflect a compensatory metabolic response to elevated energy demands or impaired glucose utilization by chondrocytes and synoviocytes [21]. Citrate is generated in the mitochondria and, once exported to the cytosol, can be converted into acetyl-CoA, providing carbon units and reducing equivalents required for lipid synthesis and membrane formation. This pathway is particularly relevant in processes involving cellular growth and proliferation. In the context of joint inflammation, alterations in citrate metabolism may therefore reflect both energetic imbalance and changes in cellular activation [36].

Beyond its metabolic role, citrate has increasingly been recognized as a regulator of immune and inflammatory responses. A previous study demonstrated that increased citrate concentrations following cryotherapy in humans with osteoarthritis were associated with a potential inhibitory effect on inflammation, attributed to reduced citrate conversion into acetyl-CoA and a concomitant decrease in inflammatory mediators such as reactive oxygen species, nitric oxide, and prostaglandins [37]. Collectively, these findings support the hypothesis that elevated citrate levels in the synovial fluid of the osteoarthritis group may represent an adaptive response aimed at modulating inflammation and oxidative stress within the joint environment.

The present results are consistent with previous reports describing increased citrate signals in inflammatory joint conditions; however, they differ with respect to glucose metabolism. A prior study investigating equine joint diseases reported higher signals for glucose, citrate, and other metabolites in inflammatory conditions, including osteoarthritis and osteochondrosis, when compared with septic arthritis [21]. In contrast, the present study demonstrated a less intense α-glucose signal in the osteoarthritis group, which may be explained by the nonspecific nature of glucose as a biomarker and its rapid utilization by metabolically active tissues [21].

Furthermore, the increased choline signal observed in the osteoarthritis group suggests enhanced phospholipid turnover and membrane remodeling, processes commonly associated with inflammatory cell activation and synovial hyperplasia. Choline may also function as an osmolyte, contributing to membrane stability under inflammatory stress. While early stages of osteoarthritis are often characterized by metabolites predominantly related to glycolysis, more advanced stages tend to exhibit shifts toward altered energy metabolism and increased glucose consumption [38]. Other metabolites associated with energy metabolism, such as lactate, alanine, and acetate, were also detected but appeared to play a secondary role, as indicated by their lower signal intensities and weaker correlations with ultrasonographic scores.

### 4.7. Lipid Peroxidation Analysis

The higher TBARS concentrations observed in the osteoarthritis group indicate increased lipid peroxidation within the joint environment, reflecting enhanced oxidative damage [19,20]. This finding is consistent with the inflammatory nature of osteoarthritis, in which overproduction of reactive oxygen species (ROS) by activated chondrocytes and synovial cells contributes to oxidative damage of membrane lipids, proteins, and DNA [21]. Elevated lipid peroxidation markers, such as TBARS, have been associated with the degradation of cartilage matrix components, particularly collagen and proteoglycans, thereby accelerating joint degeneration. The imbalance between pro-oxidant and antioxidant mechanisms in osteoarthritic joints disrupts redox homeostasis, perpetuating inflammation and tissue injury. These results reinforce the relevance of lipid peroxidation markers as potential indicators of disease activity and therapeutic response. However, the specificity of TBARS quantification has been questioned in recent studies [18] and should be considered in future investigations.

## 5. Conclusions

Despite providing valuable insights into joint metabolism in spontaneous osteoarthritis compared with healthy joints, this study included horses with different disease stages, severities, and athletic backgrounds, which may have influenced the results. Future studies based on controlled experimental designs are needed to corroborate these findings and clarify the observed associations.

The metabolomic profile obtained reinforces the potential of ^1^H NMR-based metabolomics as a sensitive approach for detecting biochemical alterations in equine osteoarthritis and highlights citrate as a promising biomarker. The increased TBARS concentrations indicate that lipid peroxidation plays a key role in the pathophysiology of metacarpophalangeal osteoarthritis in horses. Their correlation with ultrasonographic signs of acute synovitis suggests their potential as early-stage biomarkers of the disease.

Overall, these results show that osteoarthritis is characterized not only by structural and inflammatory alterations, such as osteophyte formation, effusion, and capsular thickening, but also by metabolic reprogramming within the joint microenvironment. Nevertheless, further studies with more homogeneous clinical cohorts are required to validate these findings and strengthen their applicability in early diagnosis.

## Figures and Tables

**Table 1:** Score system for radiographic parameters of structures from metacarpophalangeal joints, according to Trumble et al. (2008) [24], that resulted in a radiographic score for each joint (0–24).

PARAMETER	SCORE			
	0	1	2	3
subchondral bone sclerosis	absent	mild	moderate	severe
soft tissue	normal	mild thickening	moderate thickening	mineralization
narrowing of the joint space	absent	<3 mm	3–5 mm	>6 mm
number of oste/entheseophytes	absent	1 a 2	3 a 4	>4
size of oste/entheseophytes	absent	small	medium	large
number of fragments	absent	1	2	>3
size of fragments	absent	small	medium	large

**Table 2:** Ultrasonographic binary scoring system for intra-articular structures of the equine metacarpophalangeal joint, adapted from Reef (1998) [26]. Normal findings were scored as 0, and abnormal findings as 1. The sum of individual parameters resulted in the final ultrasonographic score for each joint. Blank cells indicate non-applicable classification.

Structure	Size (mm)	Echogenicity	Echotexture	Shape	Fragment/Mineralization	Fiber Alignment
Synovial membrane	≤5.0 (0); >5.1 (1)	Echoic (0); Hypo-/Hyperechoic (1)	Homogeneous (0); Heterogeneous (1)	Regular (0); Irregular (1)	Absent (0); Present (1)	—
Synovial plica	≤5.0 (0); >5.1 (1)	Hypoechoic (0); Anechoic/Hyperechoic (1)	Homogeneous (0); Heterogeneous (1)	Regular (0); Irregular (1)	Absent (0); Present (1)	—
Bone contour	—	Hyperechoic (0); Hypoechoic (1)	Homogeneous (0); Heterogeneous (1)	Regular (0); Irregular (1)	Absent (0); Present (1)	—
Synovial fluid	Normal (0); increased/Decreased (1)	Anechoic (0); Hypo-/Hyperechoic (1)	—	—	—	—
LSCL	≤4.5 (0); >4.6 (1)	Echoic (0); Hypo-/Hyperechoic (1)	Homogeneous (0); Heterogeneous (1)	Regular (0); Irregular (1)	Absent (0); Present (1)	Parallel (0); Disorganized (1)
LLCL	≤4.5 (0); >4.6 (1)	Echoic (0); Hypo-/Hyperechoic (1)	Homogeneous (0); Heterogeneous (1)	Regular (0); Irregular (1)	Absent (0); Present (1)	Parallel (0); Disorganized (1)
MSCL	≤4.5 (0); >4.6 (1)	Echoic (0); Hypo-/Hyperechoic (1)	Homogeneous (0); Heterogeneous (1)	Regular (0); Irregular (1)	Absent (0); Present (1)	Parallel (0); Disorganized (1)
MLCL	≤4.5 (0); >4.6 (1)	Echoic (0); Hypo-/Hyperechoic (1)	Homogeneous (0); Heterogeneous (1)	Regular (0); Irregular (1)	Absent (0); Present (1)	Parallel (0); Disorganized (1)

Abbreviations: LSCL, lateral short collateral ligament; LLCL, lateral long collateral ligament; MSCL, medial short collateral ligament; MLCL, medial long collateral ligament.

**Table 3:** Descriptive statistics, normality assessment, and Mann–Whitney U test for comparative analysis of demographic, orthopedic, and imaging data between the control and osteoarthritis groups.

Parameters		Control Group	Osteoarthritis Group	*p* Value
		Mean ± Standard Deviation	Mean ± Standard Deviation	
Demographic data	Age (years)	3.55 ^a^ ± 1.21	9.14 ^b^ ± 5.89	0.0085
	Weight (Kg)	332.00 ^a^ ± 27.07	466.70 ^b^ ± 38.06	<0.0001
Imaging scores	Sum of image scores	3.27 ^a^ ± 0.79	17.51 ^b^ ± 6.84	<0.0001
	Radiographic scores	0.91 ^a^ ± 0.83	8.81 ^b^ ± 5.58	<0.0001
	Ultrasonographic scores	2.36 ^a^ ± 0.50	8.70 ^b^ ± 3.32	<0.0001
Orthopedic scores	Joint distention	0 ^a^ ± 0	0.57 ^a^ ± 0.69	0.1479
	Local heat	0 ^a^ ± 0	0.35 ^a^ ± 0.59	0.4276
	Pain on palpation	0 ^a^ ± 0	0.27 ^a^ ± 0.61	0.5864
	Lameness	0 ^a^ ± 0	0.59 ^a^ ± 1.07	0.3872
	Lameness in flexion	0 ^a^ ± 0	1.14 ^a^ ± 1.16	0.0584

Groups sharing the same letter (a or b) do not differ significantly, while different letters denote statistically significant differences.

**Table 4:** Descriptive analysis was performed for all synovial fluid parameters. Normality was assessed using the Shapiro–Wilk test. The Mann–Whitney U test was applied to TBARS and all physicochemical and cytological variables that did not meet normality assumptions, whereas Student’s *t*-test was used for C2C, which passed the normality test.

Parameters of Synovial Fluid		Control Group	Osteoarthritis Group	*p* Value
		Mean ± Standard Deviation	Mean ± Standard Deviation	
Physicochemical	Volume (mL)	1.60 ^a^ ± 0.80	5.80 ^b^ ± 2.50	0.0032
	Color (score)	0.91 ^a^ ± 1.58	2.00 ^a^ ± 1.31	0.0594
	Turbidity (score)	0.82 ^a^ ± 1.25	0.68 ^a^ ± 0.88	0.7527
	Viscosity (score)	3.36 ^a^ ± 1.12	3.08 ^a^ ± 1.50	0.9716
	Total protein (g/dL)	0.51 ^a^ ± 0.21	1.07 ^b^ ± 0.43	<0.0001
	Mucin test (score)	0.64 ^a^ ± 0.67	1.97 ^a^ ± 1.22	0.0654
	pH	7.64 ^a^ ± 0.32	8.11 ^b^ ± 0.67	0.0089
Cytology	TNCC (cells/μL)	114.60 ^a^ ± 72.07	96.05 ^a^ ± 85.30	0.107
	Neutrophils (%)	19.88 ^a^ ± 11.46	11.86 ^b^ ± 13.61	0.043
	Lymphocytes (%)	19.13 ^a^ ± 9.93	18.36 ^a^ ± 15.86	0.900
	Large mononucleated cells (%)	59.25 ^a^ ± 18.57	69.68 ^a^ ± 20.41	0.092
	Eosinophils (%)	1.75 ^a^ ± 1.04	0.09 ^b^ ± 0.29	<0.001
Cartilage degradation	C2C (ng/mL)	4739 ^a^ ± 21.29	78.03 ^b^ ± 30.73	0.011
Oxidative damage	TBARS (ng/mL)	603.14 ^a^ ± 144.43	637.77 ^b^ ± 152.43	0.009

Groups sharing the same letter (a or b) do not differ significantly, while different letters denote statistically significant differences. Abbreviations: TNCC—total nucleated cell count; C2C—type II collagen degradation biomarker; and TBARS—thiobarbituric acid reactive substances.

## Data Availability

The data presented in this study are openly available in the 2026 Journal of Veterinary Science—Supplementary Material, “Ultrasonographic evidence of synovitis correlates with synovial citrate and TBARS in equine osteoarthritis”, at https://drive.google.com/drive/u/0/folders/1PZdJ8j9BaZzBjw_rCm7iG4cedCkPVcOn, accessed on 18 December 2025. Complete data table, image scoring, and all statistical analysis will be made available.

## Supplementary Materials

The following are available online at https://www.mdpi.com/article/10.3390/vetsci13020140/s1. The following supporting information can be downloaded at https://drive.google.com/drive/u/0/folders/1PZdJ8j9BaZzBjw_rCm7iG4cedCkPVcOn, accessed on 18 December 2025. Complete data table, image scoring and all statistical analysis.

## Abbreviations

The following abbreviations are used in this manuscript:

OAG	Osteoarthritis group
CG	Control group
^1^H NMR	Hydrogen nuclear magnetic resonance spectroscopy
TBARS	Thiobarbituric acid reactive substances
C2C	Type II collagen degradation biomarker
CPII	Type II collagen C-pro-peptide biomarker
CS846	Chondroitin sulfate 846 epitope biomarker
HA	Hyaluronan
MRI	Magnetic resonance imaging
MDA	Malondialdehyde
EDTA	Ethylenediaminetetraacetic acid
PCA	Principal component analysis
PLS-DA	Partial least squares discriminant analysis
VIP	Variable importance in projection
