# Supplementary material for: Ultrasonographic Evidence of Synovitis Correlates with Synovial Citrate and TBARS in Equine Osteoarthritis
Source: Vet Sci. 2026 Jan 31;13(2):140. doi: 10.3390/vetsci13020140 (PMC12945042; doi:10.3390/vetsci13020140)
Supplement: Supplementary file 1 [file vetsci-13-00140-s001.zip › 2025 Supplementary files/2025 1 DATA TABLE - descriptive analysis.pdf]

SAMPLE ID	GROUP	ANIMAL	Image scores			Registration data				Orhtopedic exam					Physical-chemical of synovial fluid							Cytological analysis of syn		
			SUM	Radio-graphic	Ultra-sonographic	equestrian discipline	sex	age (years)	wheight (Kg)	joint distention (score)	local heat (score)	pain on palpation (score)	lameness in trot (score)	lameness in flexion (score)	volume (mL)	color (score)	turbidity (score)	viscosity (score)	total ptn (g/dL)	mucine (score)	ph	TNCC (cells/µL)	Neutrophils (%)	Limphocytes (%)
2	CG	Dilúvio MTE	2	0	2	1	2	5	345	0	0	0	0	0	0,8	1	2	3	0,8	1	8	40		
11	CG	Gabarito MTD	2	0	2	1	2	2	286	0	0	0	0	0	2,4	0	0	2	0,2	0	7,5	140	18	29
1	CG	Dilúvio MTD	3	1	2	1	2	5	345	0	0	0	0	0	1,6	0	0	3	0,6	1	7,5	100	35	32
3	CG	Disparada MTD	3	0	3	1	1	5	325	0	0	0	0	0	0,4	4	3	5	0,4	0	8			
5	CG	Eleita MTD	3	1	2	1	1	3	380	0	0	0	0	0	0,5	0	0	5	0,6	1	8	200	27	24
9	CG	Encantada MTD	3	0	3	1	1	3	335	0	0	0	0	0	2,2	0	0	2	0,4	1	7,5	30		
4	CG	Disparada MTE	4	2	2	1	1	5	325	0	0	0	0	0	1,4	0	0	5	0,4	1	7	200	10	6
7	CG	Empoderada M	4	1	3	1	1	3	345	0	0	0	0	0	2,4	1	1	3	0,8	0	8	10	6	10
8	CG	Empoderada M	4	1	3	1	1	3	345	0	0	0	0	0	2,6	0	0	3	0,6	0	7,5	200	27	24
10	CG	Encantada MTE	4	2	2	1	1	3	335	0	0	0	0	0	1,6	4	3	3	0,6	0	7,5	100	6	20
12	CG	Gabarito MTE	4	2	2	1	2	2	286	0	0	0	0	0	2,2	0	0	3	0,2	2	7,5	126	30	8
**CG**		**MIN**	2	0	2	1	1	2	286	0	0	0	0	0	0,4	0	0	2	0,2	0	7	10	6	6
**CG**		**MAX**	4	2	3	1	2	5	380	0	0	0	0	0	2,6	4	3	5	0,8	2	8	200	35	32
**CG**		**MEAN**	3,27	0,91	2,36	1,00	1,36	3,55	332,00	0,00	0,00	0,00	0,00	0,00	1,6	0,91	0,82	3,36	0,51	0,64	7,64	114,60	19,88	19,13
**CG**		**MEDIAN**	3,00	1,00	2,00	1,00	1,00	3,00	335,00	0,00	0,00	0,00	0,00	0,00	1,6	0,00	0,00	3,00	0,60	1,00	7,50	113,00	22,50	22,00
**CG**		**NDARD DEVIAT**	0,79	0,83	0,50	0,00	0,50	1,21	27,07	0,00	0,00	0,00	0,00	0,00	0,8	1,58	1,25	1,12	0,21	0,67	0,32	72,07	11,46	9,93
27	OAG	Love is forever	6	2	4	2	1	5	470	0	0	0	0	0	2,5	0	0	3	1,0	0	8			
40	OAG	Lemmon MTE	8	1	7	2	2	17	470	0	0	1	0	1	2,0	1	0	2	1,0	2	9	100	2	14
45	OAG	Pinguim MTD	9	5	4	3	2	12	400	1	0	0	0	0	6,0	4	1	3	1,0	3	9			
30	OAG	Luke MTE	11	5	6	2	2	4	439	0	0	0	0	3	7,0	1	1	3	1,0	3	8	30		
32	OAG	Oscar winner M	11	5	6	2	2	4	489	0	0	0	0	0	8,0	2	1	5	1,0	0	8		0	19
51	OAG	Zélia MTD	11	4	7	3	1	17	430	1	0	0	0	3	6,0	1	1	3	1,0	3	8			
17	OAG	Caetano da Ser	11	2	9	2	2	6	475	0	1	0	1	1	5,0	3	2	10	1,0	0	8	30		
49	OAG	Xtreme MTD	12	5	7	3	2	11	554	0	2	0	0	0	8,0	1	0	2	1,0	2	8			
16	OAG	Best yet MTE	12	5	7	2	2	4	496	1	0	0	0	0	7,0	1	0	3	1,0	2	8	20	14	41
28	OAG	Love is forever	12	6	6	2	1	5	470	2	0	2	0	1	10,0	2	1	3	2,4	3	8			
39	OAG	Lemmon MTD	13	3	10	3	2	17	470	2	1	1	2	2	3,5	1	0	3	1,0	2	9	100	26	28
31	OAG	Oscar winner M	13	3	10	2	2	4	489	1	0	0	0	2	8,0	1	1	5	0,8	0	8	100	0	2
29	OAG	Luke MTD	13	7	6	2	2	4	439	0	0	0	1	2	10,0	1	0	4	1,2	3	8	35	2	10
34	OAG	Alpargata MTE	13	8	5	3	1	11	470	0	0	0	2	2	1,0	1	0	3	1,0	0	8	80	15	61
37	OAG	Dommis MTD	14	5	9	3	2	8	450	0	0	0	2	2	2,0	4	0	3	1,0	1	8	230	18	40
26	OAG	Ishtevish MTE	14	4	10	2	2	3	470	2	1	0	0	2	5,0	1	0	5	0,6	3	7	70	1	7
52	OAG	Zélia MTE	15	8	7	3	1	17	430	0	1	0	2	2	2,0	4	0	2	1,0	3	9			
15	OAG	Best yet MTD	15	6	9	2	2	4	496	1	0	0	0	0	8,0	1	0	3	0,8	3	8	25	4	30
21	OAG	End one MTD	15	3	12	2	2	3	480	0	0	0	3	0	6,5	1	1	3	1,0	3	7	100	38	2
33	OAG	Alpargata MTD	15	9	6	3	1	11	470	0	0	0	0	1	2,0	1	0	3	1,0	0	8	100	20	10
19	OAG	Dreher MTD	16	8	8	2	2	3	417	0	1	0	0	0	6,0	4	1	3	0,6	3	8		3	12
22	OAG	End one MTE	17	10	7	2	2	3	480	0	0	0	0	0	6,0	3	1	2	1,0	3	7		54	1
50	OAG	Xtreme MTE	18	6	12	3	2	11	554	0	1	0	0	0	7,0	1	0	1	1,0	3	8			
44	OAG	Neanderthal M	20	16	4	3	2	15	455	0	0	0	0	0	4,0	4	0	2	1,0					
36	OAG	Dengosa MTE	22	16	6	3	1	18	400	1	1	0	0	3	8,0	2	0	2	1,0	2	9			
43	OAG	Neanderthal M	22	15	7	3	2	15	455	0	0	0	0	0	3,0	1	1	4	1,0		9			
35	OAG	Dengosa MTD	23	15	8	3	1	18	400	0	1	0	0	0	8,0	1	0	2	1,0	2	9			
24	OAG	Farawhip MTE	23	7	16	2	2	6	490	2	0	2	0	1	9,0	4	2	2	1,0	3	7	85	13	16
25	OAG	Ishtevish MTD	24	8	16	2	2	3	470	1	0	0	0	1	4,5	1	0	4	0,6	0	8		0	0
23	OAG	Farawhip MTD	24	8	16	2	2	6	490	1	0	2	3	3	6,0	4	3	2	2,4	3	7	30	9	33
41	OAG	Milongueiro MT	25	17	8	3	2	16	499	1	2	1	4	4	4,0	1	0	3	1,0		9			
42	OAG	Milongueiro MT	26	17	9	3	2	16	499	0	0	1	1	2	7,0	1	0	3	1,0	2	9			
18	OAG	Caetano da Ser	27	17	10	2	2	6	475	1	0	0	1	1	5,0	3	2	2	0,8	3	7	140	5	27
14	OAG	A todo vapor M	28	17	11	2	2	5	505	1	0	0	0	2	10,0	4	3	2	2,4	0	9	150	20	10
13	OAG	A todo vapor M	29	20	9	2	2	5	505	1	0	0	0	1	8,0	2	2	3	1,2	2	8	30	7	16
20	OAG	Dreher MTE	29	15	14	2	2	3	417	1	1	0	0	0	5,0	4	1	2	0,4	3	8		5	0
48	OAG	Soledad MTE	32	18	14	3	1	22	400	0	0	0	0	0	3,0	2	0	4	1,0	2	8	370	5	25
**OAG**		**MIN**	6	1	4	2	1	3	400	0	0	0	0	0	1,0	0	0	1	0,4	0	7	20	0	0
**OAG**		**MAX**	32	20	16	3	2	22	554	2	2	2	4	4	10,0	4	3	10	2,4	3	9	370	54	61
**OAG**		**MEAN**	17,51	8,81	8,70	2,43	1,76	9,14	466,70	0,57	0,35	0,27	0,59	1,14	5,8	2,00	0,68	3,08	1,07	1,97	8,11	96,05	11,86	18,36
**OAG**		**MEDIAN**	15,00	7,00	8,00	2,00	2,00	6,00	470,00	0,00	0,00	0,00	0,00	1,00	6,0	1,00	0,00	3,00	1,01	2,00	8,00	85,00	6,00	15,00
**OAG**		**NDARD DEVIAT**	6,84	5,58	3,32	0,50	0,43	5,89	38,06	0,69	0,59	0,61	1,07	1,16	2,5	1,31	0,88	1,50	0,43	1,22	0,67	85,30	13,61	15,86

novial fluid		Oxdative stress	Cartilage degradation	Metabolomic						
Large mononucleated cells (%)	Eosinophils (%)	TBARS (ng/mL)	C2C (ng/mL)	Citrate (2.54)	Choline (3.20)	Leucine (0.99)	Lactato (1.32)	Alanine (1.46)	Acetato (1.90)	Alfa-Glucose (5.22)
			63,66							
51	2	420,50	50,50	0,0010	0,0020	0,0072	0,0245	0,0069	0,0053	0,0019
32	1		15,97	0,0012	0,0025	0,0069	0,0259	0,0076	0,0048	0,0023
46	3	470,73								
		429,06	79,11							
83	1	464,85								
82	2	504,10	63,66							
46	3	479,01	26,56							
72	2	541,57	46,61	0,0010	0,0024	0,0088	0,0193	0,0065	0,0042	0,0028
62	0	441,86	33,06	0,0008	0,0021	0,0073	0,0253	0,0071	0,0053	0,0019
32	0	420,50	15,97	0,0008	0,0020	0,0069	0,0193	0,0065	0,0042	0,0019
83	3	541,57	79,11	0,0012	0,0025	0,0088	0,0259	0,0076	0,0053	0,0028
59,25	1,75	468,96	47,39	0,00	0,00	0,01	0,02	0,01	0,00	0,00
56,50	2,00	467,79	48,56	0,00	0,00	0,01	0,02	0,01	0,01	0,00
18,57	1,04	40,18	21,29	0,00	0,00	0,00	0,00	0,00	0,00	0,00
		742,36	101,78	0,0050	0,0054	0,0011	0,0096	0,0013	0,0006	0,0006
84	0	559,43	47,91	0,0026	0,0016	0,0019	0,0075	0,0024	0,0011	0,0005
		448,68	31,80	0,0019	0,0029	0,0017	0,0156	0,0036	0,0014	0,0020
		559,15	57,64	0,0054	0,0110	0,0044	0,0106	0,0035	0,0017	0,0006
81	0	635,93	95,61	0,0055	0,0047	0,0015	0,0107	0,0013	0,0005	0,0007
		569,15	71,46	0,0036	0,0016	0,0022	0,0180	0,0035	0,0013	0,0019
		1026,65	140,40	0,0050	0,0183	0,0031	0,0121	0,0046	0,0019	0,0006
		607,34	87,86	0,0047	0,0016	0,0019	0,0169	0,0059	0,0022	0,0017
45	0	507,64	86,02	0,0045	0,0060	0,0036	0,0069	0,0029	0,0018	0,0007
		638,85	151,13	0,0060	0,0041	0,0011	0,0167	0,0034	0,0007	0,0007
46	0	531,97	87,86	0,0043	0,0220	0,0020	0,0099	0,0034	0,0015	0,0006
98	0	721,95	119,67	0,0055	0,0052	0,0018	0,0083	0,0025	0,0009	0,0008
88	0	1020,82	96,62	0,0061	0,0105	0,0049	0,0107	0,0037	0,0020	0,0007
24	0	491,51	84,46	0,0221	0,4000	0,0016	0,0037	0,0012	0,0009	0,0004
42	0	568,10	55,80	0,0047	0,0068	0,0012	0,0099	0,0024	0,0010	0,0006
92	0	583,45	92,80	0,0046	0,0546	0,0030	0,0130	0,0050	0,0015	0,0007
		392,85	109,36	0,0031	0,0017	0,0023	0,0179	0,0039	0,0012	0,0018
66	0	598,03	109,32	0,0046	0,0057	0,0037	0,0070	0,0030	0,0090	0,0007
60	0	786,10	93,97	0,0048	0,0091	0,0032	0,0098	0,0050	0,0014	0,0009
70	0	591,23	115,33	0,0035	0,0196	0,0013	0,0047	0,0016	0,0011	0,0006
85	0	666,55	52,78	0,0053	0,0045	0,0003	0,0089	0,0012	0,0006	0,0007
44	1	963,96	89,52	0,0048	0,0653	0,0041	0,0112	0,0051	0,0016	0,0008
		492,75	92,80	0,0037	0,0016	0,0031	0,0192	0,0020	0,0022	0,0016
		517,73	26,11	0,0020	0,0023	0,0031	0,0154	0,0013	0,0014	0,0020
		489,81	91,54	0,0022	0,0019	0,0024	0,0130	0,0009	0,0002	0,0023
		627,92	34,54	0,0017	0,0023	0,0027	0,0144	0,0012	0,0017	0,0017
		525,07	66,73	0,0029	0,0016	0,0020	0,0149	0,0024	0,0018	0,0021
71	0	618,44	68,45	0,0053	0,1515	0,0041	0,0080	0,0039	0,0017	0,0008
100	0	637,39	35,62	0,0050	0,0075	0,0021	0,0130	0,0049	0,0011	0,0007
58	0	627,92	44,20	0,0047	0,0500	0,0037	0,0132	0,0040	0,0019	0,0007
		613,22	31,35	0,0018	0,0014	0,0028	0,0135	0,0045	0,0027	0,0025
		488,34	42,20	0,0013	0,0021	0,0014	0,0174	0,0012	0,0017	0,0021
68	0	596,57	67,97	0,0051	0,0153	0,0030	0,0122	0,0045	0,0019	0,0007
69	1	870,66	67,97	0,0049	0,0247	0,0033	0,0098	0,0049	0,0026	0,0007
77	0	536,80	67,97	0,0050	0,1474	0,0042	0,0100	0,0046	0,0020	0,0008
95	0	596,57	64,18	0,0055	0,0047	0,0016	0,0085	0,0011	0,0005	0,0007
70	0	523,30	106,34	0,0045	0,0054	0,0011	0,0079	0,0014	0,0009	0,0007
24	0	392,85	26,11	0,001294064	0,001374466	0,000311279	0,003664542	0,000893841	0,000208828	0,000392351
100	1	1026,65	151,13	0,022121435	0,399971493	0,004906042	0,019173855	0,005863626	0,00901668	0,002480627
69,68	0,09	620,92	78,03	0,0047	0,0292	0,0025	0,0116	0,0031	0,0016	0,0011
70,00	0,00	596,57	84,46	0,0047	0,0054	0,0023	0,0107	0,0034	0,0015	0,0007
20,41	0,29	147,57	30,73	0,0032	0,0718	0,0011	0,0038	0,0015	0,0014	0,0006
