# Supplementary material for: Ultrasonographic Evidence of Synovitis Correlates with Synovial Citrate and TBARS in Equine Osteoarthritis
Source: Vet Sci. 2026 Jan 31;13(2):140. doi: 10.3390/vetsci13020140 (PMC12945042; doi:10.3390/vetsci13020140)
Supplement: Supplementary file 1 [file vetsci-13-00140-s001.zip › 2025 Supplementary files/2025 2 Statistics of metabolomic analysis - raw data.pdf]

1H Chemical Shift, ppm	P value	Mean rank of Control	Mean rank of OA	Mean ran k diff.	me	q value	- log10(q valu e)
2,26	0,00001	39,5	19	20,5	0	0,000036	4,439
0,99	0,00002	39,5	19	20,5	0	0,000036	4,439
1,04	0,00002	39,5	19	20,5	0	0,000036	4,439
1,05	0,00002	39,5	19	20,5	0	0,000036	4,439
1,06	0,00002	39,5	19	20,5	0	0,000036	4,439
1,07	0,00002	39,5	19	20,5	0	0,000036	4,439
1,08	0,00002	39,5	19	20,5	0	0,000036	4,439
1,1	0,00002	39,5	19	20,5	0	0,000036	4,439
1,21	0,00002	39,5	19	20,5	0	0,000036	4,439
1,22	0,00002	39,5	19	20,5	0	0,000036	4,439
1,31	0,00002	39,5	19	20,5	0	0,000036	4,439
1,32	0,00002	39,5	19	20,5	0	0,000036	4,439
1,33	0,00002	39,5	19	20,5	0	0,000036	4,439
1,34	0,00002	39,5	19	20,5	0	0,000036	4,439
1,46	0,00002	39,5	19	20,5	0	0,000036	4,439
1,48	0,00002	39,5	19	20,5	0	0,000036	4,439
1,49	0,00002	39,5	19	20,5	0	0,000036	4,439
1,5	0,00002	39,5	19	20,5	0	0,000036	4,439
1,51	0,00002	39,5	19	20,5	0	0,000036	4,439
1,52	0,00002	39,5	19	20,5	0	0,000036	4,439
1,9	0,00002	39,5	19	20,5	0	0,000036	4,439
1,91	0,00002	39,5	19	20,5	0	0,000036	4,439
1,92	0,00002	39,5	19	20,5	0	0,000036	4,439
1,93	0,00002	39,5	19	20,5	0	0,000036	4,439
1,94	0,00002	39,5	19	20,5	0	0,000036	4,439
2,13	0,00002	39,5	19	20,5	0	0,000036	4,439
2,14	0,00002	39,5	19	20,5	0	0,000036	4,439
2,16	0,00002	39,5	19	20,5	0	0,000036	4,439
2,27	0,00002	39,5	19	20,5	0	0,000036	4,439
2,28	0,00002	39,5	19	20,5	0	0,000036	4,439
2,29	0,00002	39,5	19	20,5	0	0,000036	4,439
2,3	0,00002	39,5	19	20,5	0	0,000036	4,439
2,44	0,00002	39,5	19	20,5	0	0,000036	4,439
2,46	0,00002	39,5	19	20,5	0	0,000036	4,439
2,47	0,00002	39,5	19	20,5	0	0,000036	4,439
2,54	0,00002	2,5	23	-20,5	0	0,000036	4,439
2,65	0,00002	39,5	19	20,5	0	0,000036	4,439
2,68	0,00002	39,5	19	20,5	0	0,000036	4,439
2,76	0,00002	2,5	23	-20,5	0	0,000036	4,439
2,78	0,00002	2,5	23	-20,5	0	0,000036	4,439
2,81	0,00002	2,5	23	-20,5	0	0,000036	4,439
2,82	0,00002	2,5	23	-20,5	0	0,000036	4,439
2,95	0,00002	2,5	23	-20,5	0	0,000036	4,439
2,97	0,00002	2,5	23	-20,5	0	0,000036	4,439
2,98	0,00002	2,5	23	-20,5	0	0,000036	4,439

3,08	0,00002	2,5	23	-20,5	0	0,000036	4,439
3,09	0,00002	2,5	23	-20,5	0	0,000036	4,439
3,1	0,00002	2,5	23	-20,5	0	0,000036	4,439
3,11	0,00002	2,5	23	-20,5	0	0,000036	4,439
3,16	0,00002	2,5	23	-20,5	0	0,000036	4,439
3,21	0,00002	2,5	23	-20,5	0	0,000036	4,439
3,26	0,00002	39,5	19	20,5	0	0,000036	4,439
3,27	0,00002	39,5	19	20,5	0	0,000036	4,439
3,37	0,00002	39,5	19	20,5	0	0,000036	4,439
3,39	0,00002	39,5	19	20,5	0	0,000036	4,439
3,9	0,00002	39,5	19	20,5	0	0,000036	4,439
3,98	0,00002	39,5	19	20,5	0	0,000036	4,439
4,08	0,00002	39,5	19	20,5	0	0,000036	4,439
4,11	0,00002	39,5	19	20,5	0	0,000036	4,439
5,77	0,00002	2,5	23	-20,5	0	0,000036	4,439
5,78	0,00002	2,5	23	-20,5	0	0,000036	4,439
5,79	0,00002	2,5	23	-20,5	0	0,000036	4,439
5,8	0,00002	2,5	23	-20,5	0	0,000036	4,439
5,81	0,00002	2,5	23	-20,5	0	0,000036	4,439
5,82	0,00002	2,5	23	-20,5	0	0,000036	4,439
5,86	0,00002	2,5	23	-20,5	0	0,000036	4,439
5,87	0,00002	2,5	23	-20,5	0	0,000036	4,439
5,88	0,00002	2,5	23	-20,5	0	0,000036	4,439
5,89	0,00002	2,5	23	-20,5	0	0,000036	4,439
5,99	0,00002	2,5	23	-20,5	0	0,000036	4,439
7,18	0,00002	39,5	19	20,5	0	0,000036	4,439
7,2	0,00002	39,5	19	20,5	0	0,000036	4,439
7,21	0,00002	39,5	19	20,5	0	0,000036	4,439
7,26	0,00002	39,5	19	20,5	0	0,000036	4,439
7,28	0,00002	39,5	19	20,5	0	0,000036	4,439
7,33	0,00002	39,5	19	20,5	0	0,000036	4,439
7,52	0,00002	39,5	19	20,5	0	0,000036	4,439
7,53	0,00002	39,5	19	20,5	0	0,000036	4,439
7,55	0,00002	39,5	19	20,5	0	0,000036	4,439
7,56	0,00002	39,5	19	20,5	0	0,000036	4,439
7,63	0,00002	39,5	19	20,5	0	0,000036	4,439
5,95	0,00003	2,625	22,99	-20,36	0,5	0,000054	4,269
1,37	0,000039	39,25	19,03	20,22	1	0,000066	4,179
1,89	0,000039	39,25	19,03	20,22	1	0,000066	4,179
2,32	0,000039	39,25	19,03	20,22	1	0,000066	4,179
2,36	0,000039	39,25	19,03	20,22	1	0,000066	4,179
2,52	0,000039	39,25	19,03	20,22	1	0,000066	4,179
6	0,000039	2,75	22,97	-20,22	1	0,000066	4,179
6,79	0,000039	39,25	19,03	20,22	1	0,000066	4,179
1,11	0,000069	39	19,05	19,95	2	0,000115	3,941
1,09	0,000079	39	19,05	19,95	2	0,000119	3,925
1,12	0,000079	39	19,05	19,95	2	0,000119	3,925
2,15	0,000079	39	19,05	19,95	2	0,000119	3,925

2,48	0,000079	39	19,05	19,95	2	0,000119	3,925
2,7	0,000079	3	22,95	-19,95	2	0,000119	3,925
2,85	0,000079	3	22,95	-19,95	2	0,000119	3,925
3,84	0,000079	39	19,05	19,95	2	0,000119	3,925
6,01	0,000079	3	22,95	-19,95	2	0,000119	3,925
7,84	0,000079	39	19,05	19,95	2	0,000119	3,925
2,77	0,000089	3,125	22,93	-19,81	2,5	0,000133	3,878
2,49	0,000138	38,75	19,08	19,67	3	0,0002	3,699
2,99	0,000138	3,25	22,92	-19,67	3	0,0002	3,699
5,98	0,000138	3,25	22,92	-19,67	3	0,0002	3,699
1,23	0,000227	38,5	19,11	19,39	4	0,000324	3,489
0,97	0,000237	38,5	19,11	19,39	4	0,000324	3,489
2,33	0,000237	38,5	19,11	19,39	4	0,000324	3,489
3,06	0,000237	3,5	22,89	-19,39	4	0,000324	3,489
3,12	0,000237	3,5	22,89	-19,39	4	0,000324	3,489
3,95	0,000237	38,5	19,11	19,39	4	0,000324	3,489
2,74	0,000267	3,625	22,88	-19,25	4,5	0,000361	3,442
2,34	0,000346	38,25	19,14	19,11	5	0,000464	3,333
2,43	0,000355	38,25	19,14	19,11	5	0,000469	3,329
2,45	0,000355	38,25	19,14	19,11	5	0,000469	3,329
0,82	0,000513	38	19,16	18,84	6	0,000663	3,179
1,03	0,000533	38	19,16	18,84	6	0,000663	3,179
1,36	0,000533	38	19,16	18,84	6	0,000663	3,179
1,47	0,000533	38	19,16	18,84	6	0,000663	3,179
2,12	0,000533	38	19,16	18,84	6	0,000663	3,179
2,25	0,000533	38	19,16	18,84	6	0,000663	3,179
3,13	0,000533	4	22,84	-18,84	6	0,000663	3,179
2,63	0,000731	4,25	22,81	-18,56	7	0,000881	3,055
0,95	0,00075	37,75	19,19	18,56	7	0,000881	3,055
1,13	0,00075	37,75	19,19	18,56	7	0,000881	3,055
1,35	0,00075	37,75	19,19	18,56	7	0,000881	3,055
2,72	0,00075	4,25	22,81	-18,56	7	0,000881	3,055
3,04	0,00075	37,75	19,19	18,56	7	0,000881	3,055
3,07	0,00075	4,25	22,81	-18,56	7	0,000881	3,055
2,84	0,00082	4,375	22,8	-18,42	7,5	0,000955	3,02
6,91	0,000997	37,5	19,22	18,28	8	0,001153	2,938
0,87	0,001027	37,5	19,22	18,28	8	0,001156	2,937
1,53	0,001047	37,5	19,22	18,28	8	0,001156	2,937
1,88	0,001047	37,5	19,22	18,28	8	0,001156	2,937
2,17	0,001047	37,5	19,22	18,28	8	0,001156	2,937
3,31	0,001047	4,5	22,78	-18,28	8	0,001156	2,937
7,37	0,001047	37,5	19,22	18,28	8	0,001156	2,937
1,38	0,001333	37,25	19,24	18,01	9	0,001461	2,835
2,31	0,001402	37,25	19,24	18,01	9	0,001493	2,826
2,8	0,001402	4,75	22,76	-18,01	9	0,001493	2,826
3,02	0,001402	37,25	19,24	18,01	9	0,001493	2,826
3,28	0,001402	37,25	19,24	18,01	9	0,001493	2,826
0,83	0,001768	37	19,27	17,73	10	0,001869	2,728

3,23	0,001856	5	22,73	-17,73	10	0,001949	2,71
0,84	0,001876	36,88	19,28	17,59	10,5	0,001956	2,709
2,75	0,001916	5,125	22,72	-17,59	10,5	0,001984	2,703
2,19	0,00234	36,75	19,3	17,45	11	0,002406	2,619
1,27	0,00239	36,75	19,3	17,45	11	0,002407	2,618
1,3	0,00239	36,75	19,3	17,45	11	0,002407	2,618
3,92	0,00239	36,75	19,3	17,45	11	0,002407	2,618
7,42	0,002903	36,5	19,32	17,18	12	0,002905	2,537
2,35	0,002953	36,5	19,32	17,18	12	0,002935	2,532
6,84	0,003002	5,5	22,68	-17,18	12	0,002954	2,53
0,86	0,003022	36,5	19,32	17,18	12	0,002954	2,53
2,18	0,003031	36,5	19,32	17,18	12	0,002954	2,53
1,95	0,003061	36,5	19,32	17,18	12	0,002964	2,528
2,38	0,003782	36,25	19,35	16,9	13	0,003638	2,439
3,25	0,00476	36	19,38	16,62	14	0,00452	2,345
5,22	0,00476	36	19,38	16,62	14	0,00452	2,345
1,54	0,005629	35,75	19,41	16,34	15	0,005311	2,275
7,27	0,005727	35,75	19,41	16,34	15	0,005371	2,27
1,24	0,006942	35,5	19,43	16,07	16	0,006407	2,193
7,41	0,00706	35,5	19,43	16,07	16	0,006407	2,193
1	0,00709	35,5	19,43	16,07	16	0,006407	2,193
1,96	0,00709	35,5	19,43	16,07	16	0,006407	2,193
2,53	0,00709	6,5	22,57	-16,07	16	0,006407	2,193
2,58	0,00709	6,5	22,57	-16,07	16	0,006407	2,193
2,59	0,008364	6,75	22,54	-15,79	17	0,007512	2,124
2,2	0,008453	35,25	19,46	15,79	17	0,007547	2,122
1,25	0,009924	35	19,49	15,51	18	0,008808	2,055
7,04	0,010023	35	19,49	15,51	18	0,008843	2,053
2,66	0,010121	7	22,51	-15,51	18	0,008877	2,052
2,83	0,013676	7,5	22,46	-14,96	20	0,011729	1,931
2,01	0,013834	34,5	19,54	14,96	20	0,011729	1,931
7,36	0,013834	34,5	19,54	14,96	20	0,011729	1,931
7,38	0,013933	34,5	19,54	14,96	20	0,011729	1,931
1,99	0,014042	34,5	19,54	14,96	20	0,011729	1,931
0,92	0,014071	34,5	19,54	14,96	20	0,011729	1,931
1,98	0,014091	34,5	19,54	14,96	20	0,011729	1,931
0,93	0,01416	34,5	19,54	14,96	20	0,011729	1,931
1,97	0,01416	34,5	19,54	14,96	20	0,011729	1,931
4,09	0,01416	34,5	19,54	14,96	20	0,011729	1,931
3,34	0,016155	34,25	19,57	14,68	21	0,013308	1,876
2,24	0,016431	34,25	19,57	14,68	21	0,013461	1,871
0,98	0,01653	34,25	19,57	14,68	21	0,013468	1,871
7,34	0,019028	34	19,59	14,41	22	0,015404	1,812
1,01	0,019216	34	19,59	14,41	22	0,015404	1,812
1,55	0,019216	34	19,59	14,41	22	0,015404	1,812
1,42	0,021665	33,75	19,62	14,13	23	0,017274	1,763
1,26	0,022178	33,75	19,62	14,13	23	0,017404	1,759
2,96	0,022178	8,25	22,38	-14,13	23	0,017404	1,759

3,22	0,022178	8,25	22,38	-14,13	23	0,017404	1,759
0,88	0,02286	33,63	19,64	13,99	23,5	0,017845	1,748
0,89	0,025131	33,5	19,65	13,85	24	0,019516	1,71
3,14	0,025516	8,5	22,35	-13,85	24	0,019712	1,705
1,39	0,025931	33,38	19,66	13,71	24,5	0,019929	1,701
1,4	0,02833	33,25	19,68	13,57	25	0,021662	1,664
6,87	0,028933	8,75	22,32	-13,57	25	0,021855	1,66
2,57	0,02917	8,75	22,32	-13,57	25	0,021855	1,66
2,69	0,02917	8,75	22,32	-13,57	25	0,021855	1,66
3,03	0,02917	33,25	19,68	13,57	25	0,021855	1,66
2,79	0,032715	9	22,3	-13,3	26	0,024057	1,619
0,85	0,032912	33	19,7	13,3	26	0,024057	1,619
7,32	0,032961	33	19,7	13,3	26	0,024057	1,619
1,87	0,033238	33	19,7	13,3	26	0,024057	1,619
2,22	0,033238	33	19,7	13,3	26	0,024057	1,619
3,46	0,033238	33	19,7	13,3	26	0,024057	1,619
3,48	0,033238	33	19,7	13,3	26	0,024057	1,619
0,96	0,037681	32,75	19,73	13,02	27	0,027011	1,568
2,02	0,037681	32,75	19,73	13,02	27	0,027011	1,568
2,6	0,038906	9,375	22,26	-12,88	27,5	0,027755	1,557
1,28	0,042214	32,5	19,76	12,74	28	0,02968	1,528
1,43	0,042263	32,5	19,76	12,74	28	0,02968	1,528
1,41	0,042569	32,5	19,76	12,74	28	0,02968	1,528
0,65	0,042599	9,5	22,24	-12,74	28	0,02968	1,528
2,4	0,042599	32,5	19,76	12,74	28	0,02968	1,528
3,41	0,047931	32,25	19,78	12,47	29	0,03324	1,478
2,23	0,053283	32	19,81	12,19	30	0,03678	1,434
0,9	0,059307	31,75	19,84	11,91	31	0,04075	1,39
1,83	0,060136	10,25	22,16	-11,91	31	0,04113	1,386
1,82	0,067068	10,5	22,14	-11,64	32	0,045044	1,346
3,15	0,067068	10,5	22,14	-11,64	32	0,045044	1,346
3,17	0,067068	10,5	22,14	-11,64	32	0,045044	1,346
4,02	0,067068	31,5	19,86	11,64	32	0,045044	1,346
