# Supplementary material for: Ultrasonographic Evidence of Synovitis Correlates with Synovial Citrate and TBARS in Equine Osteoarthritis
Source: Vet Sci. 2026 Jan 31;13(2):140. doi: 10.3390/vetsci13020140 (PMC12945042; doi:10.3390/vetsci13020140)
Supplement: Supplementary file 1 [file vetsci-13-00140-s001.zip › 2025 Supplementary files/2025 3 Statistics of metabolomic and others.pdf]

Resultados Metabolômica

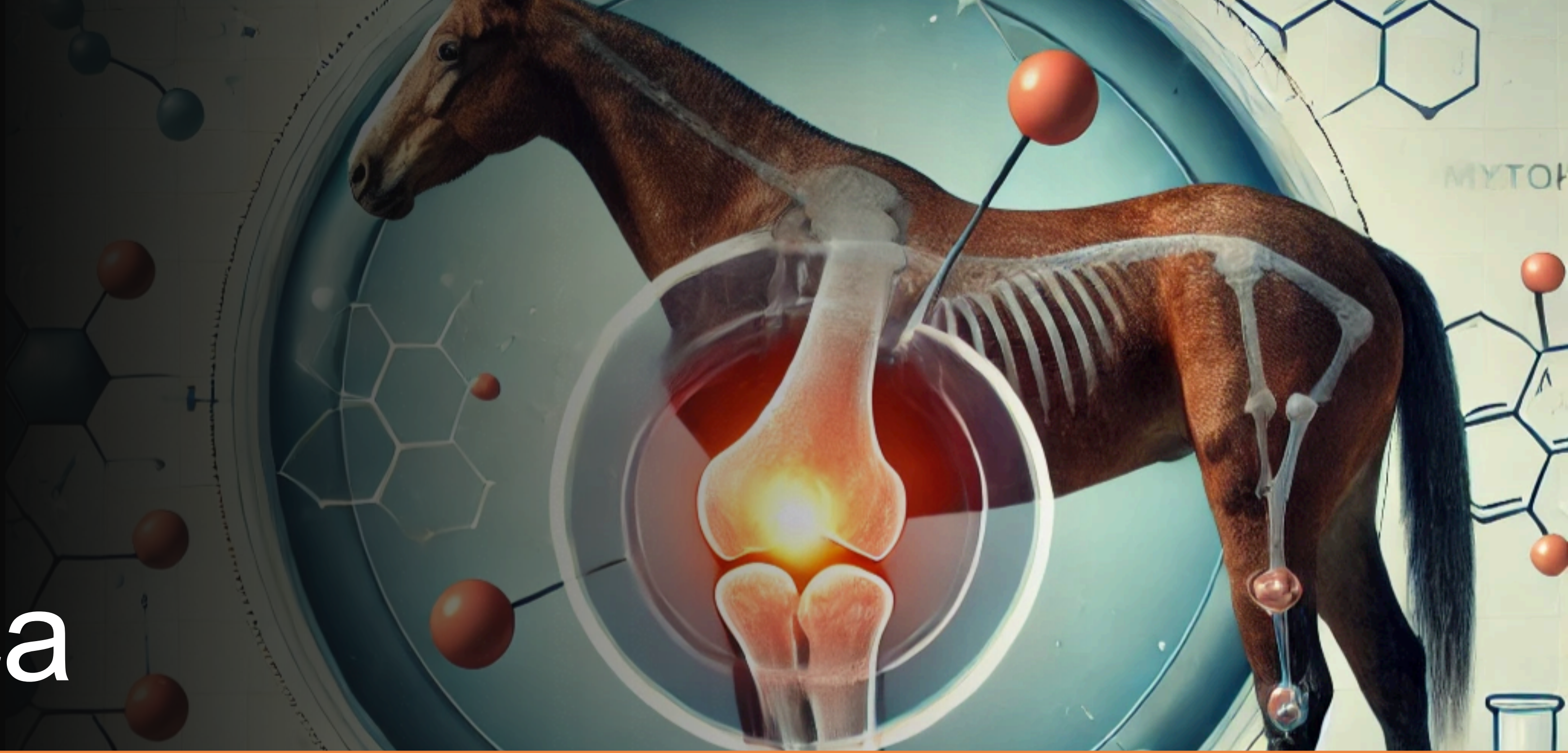

11/02/2025

LabMet

https://lab-met.com/

Data Integrity Check:

- Checking sample names - spaces will replaced with underscore, and special characters will be removed;
- Checking the class labels - at least three replicates are required in each class.
- The data (except class labels) must not contain non-numeric values.
- If the samples are paired, the pair labels must conform to the specified format.
- The presence of missing values or features with constant values (i.e. all zeros).

Data processing information:

Checking data content ...passed.

Samples are in rows and features in columns

The uploaded file is in comma separated values (.csv) format.

The uploaded data file contains 41 (samples) by 336 (compounds) data matrix.

Samples are not paired.

2 groups were detected in samples.

Only English letters, numbers, underscore, hyphen and forward slash (/) are allowed.

Other special characters or punctuations (if any) will be stripped off.

All data values are numeric.

A total of 0 (0%) missing values were detected.

By default, missing values will be replaced by 1/5 of min positive values of their corresponding variables

Click the **Proceed** button if you accept the default practice;

Or click the **Missing Values** button to use other methods.

Edit Groups | Missing Values | Proceed

PCA

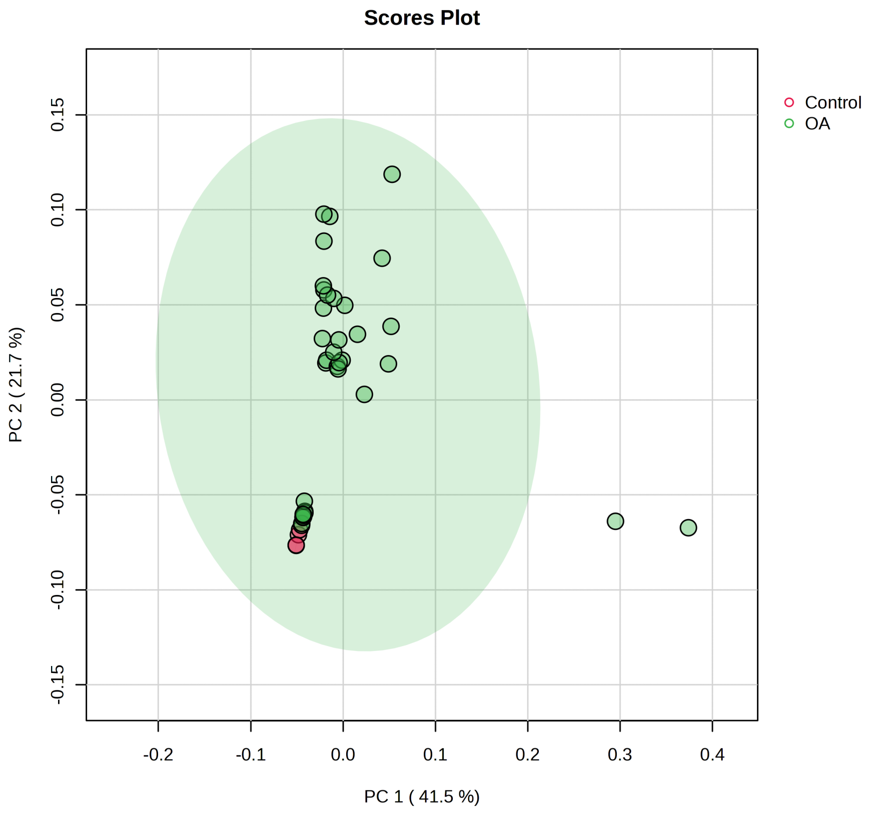

Scores Plot
Control
OA
PC 1 (41.5 %)
PC 2 (21.7 %)
-0.2
-0.1
0.0
0.1
0.2
0.3
0.4
0.15
0.10
0.05
0.00
-0.05
-0.10
-0.15

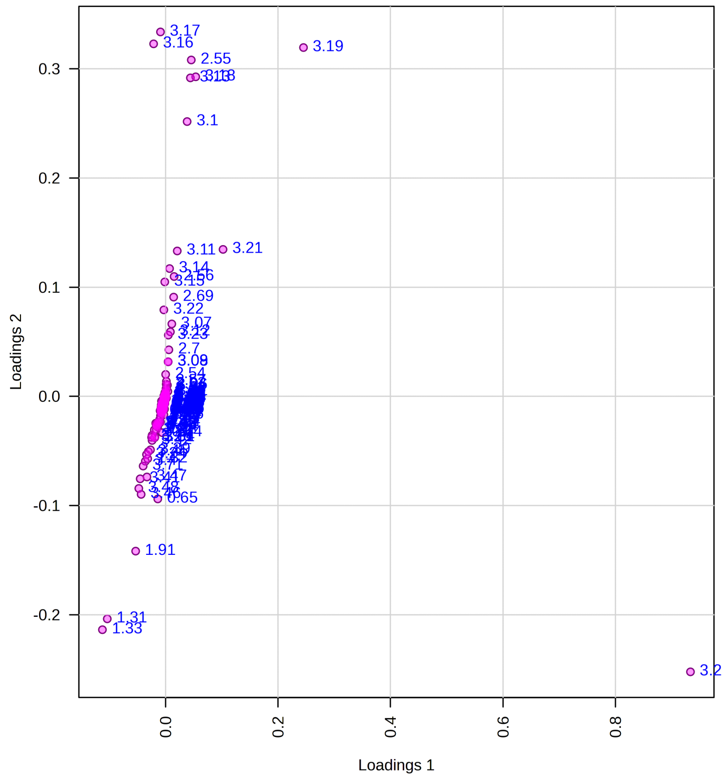

Loadings 1
Loadings 2
0.0
0.2
0.4
0.6
0.8
0.3
0.2
0.1
0.0
-0.1
-0.2
3.17
3.16
2.55
3.19
3.1
3.11
3.21
3.14
2.69
3.22
3.07
3.09
2.7
2.54
0.65
1.91
1.31
1.33
3.2

PLS-DA

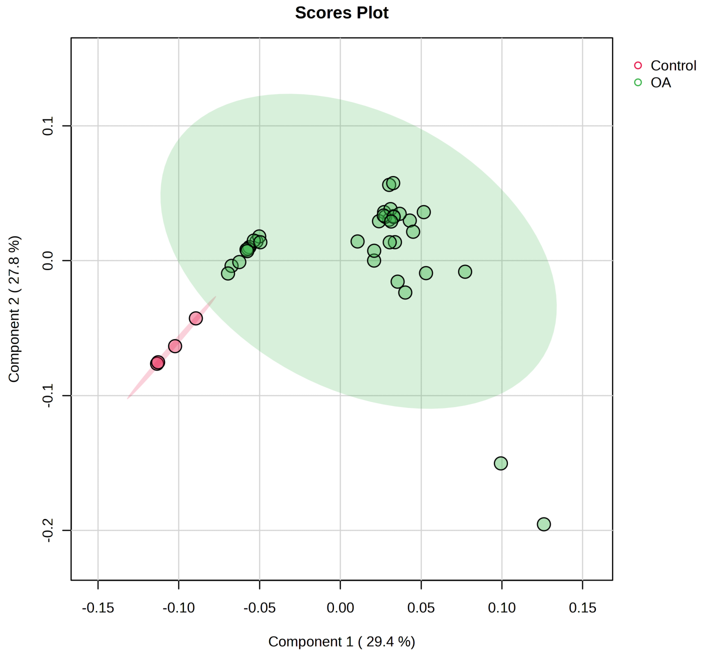

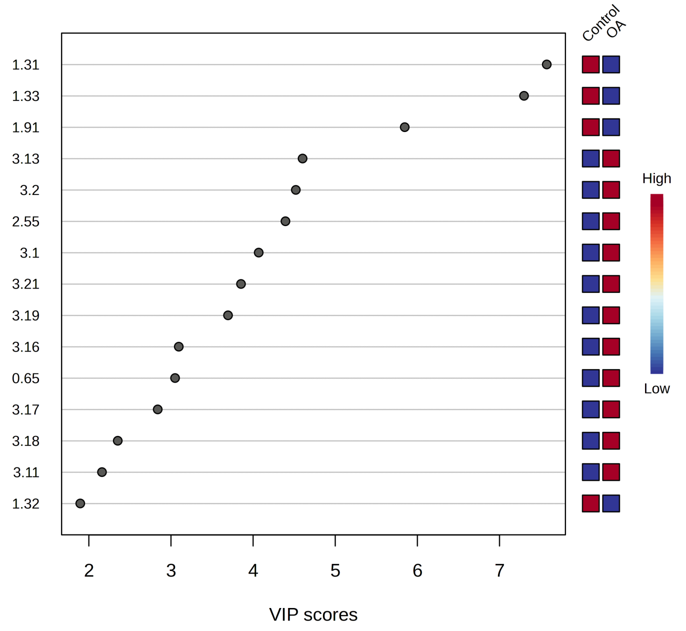

PLS-DA cross validation details:

Measure	1 comps	2 comps	3 comps	4 comps
Accuracy	0.90556	0.975	1.0	1.0
R2	0.34773	0.52992	0.80092	0.86741
Q2	0.20839	0.38403	0.70562	0.77651

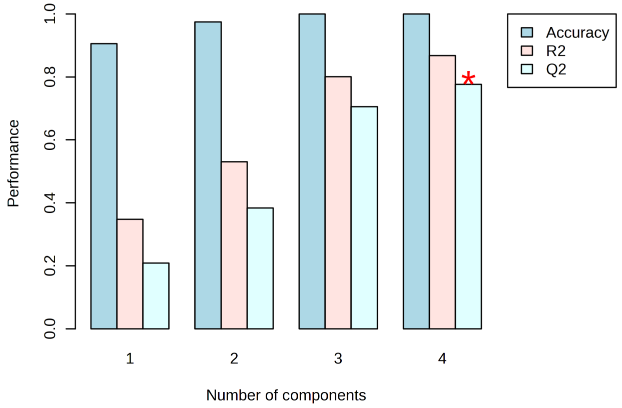

Análise univariada

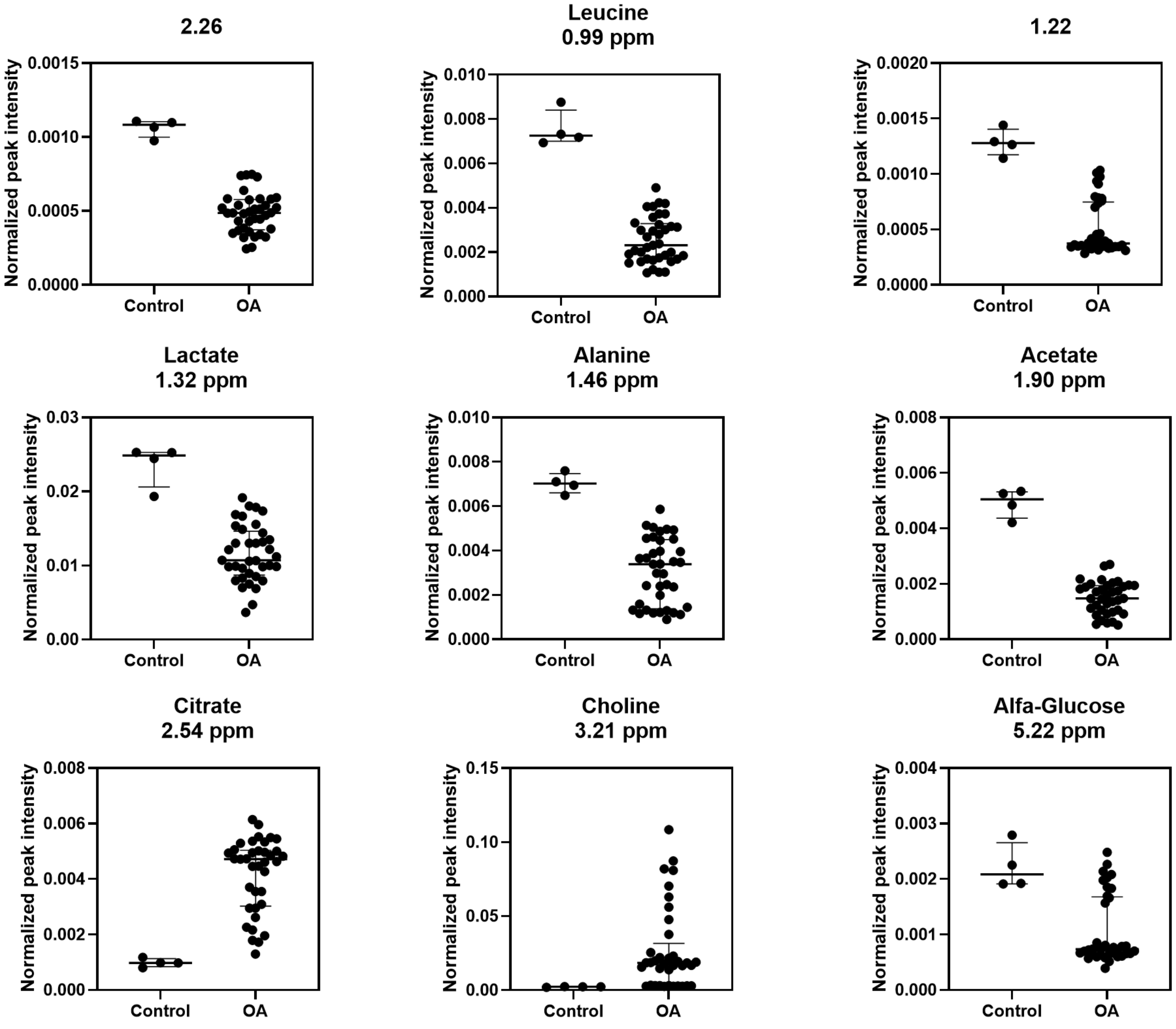

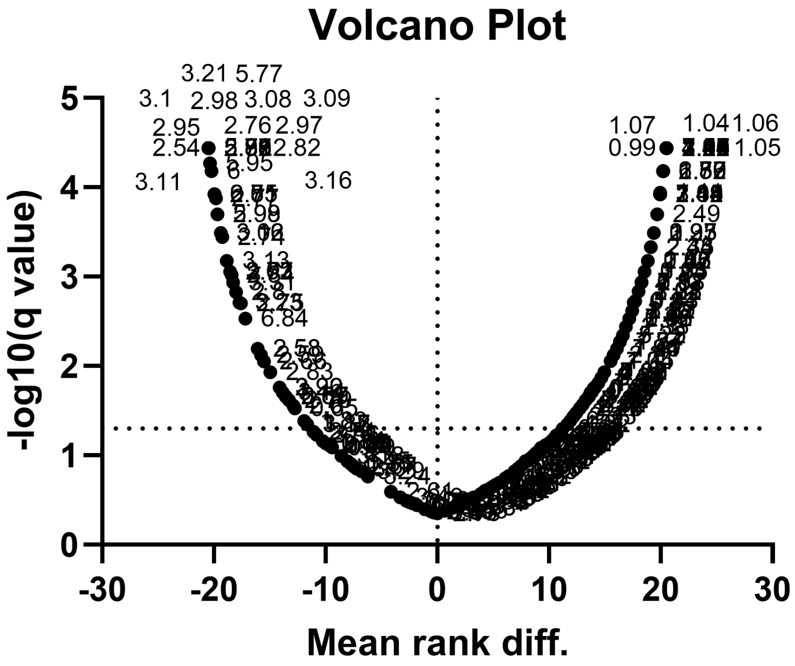

SAMPLE ID	GROUP	ANIMAL	SUM OF IMAGE SCORES	Radiographic score	Ultrasonographic score	equestrian discipline	sex	age (years)	wheight (Kg)	joint distention (efusion)	heat (score)	pain on palpation (score)	lameness in trot (score)	lameness in flexion (score)	volume (mL)	color (score)	turbidity (score)	viscosity (score)	density	total ptn (g/dL)	mucine (score)	ph	TNCC (cells/µL)	Neutrophils (%)	Limphocytes (%)	Monocytes (%)	Eosinophils (%)	TBARS (ng/mL)	C2C	CPII	CS846	HA
1	Control	Dilúvio MTD	6	1	5	pleasure	2	5	345	0	0	0	0	0	1.6	0	0	3	1010	0.6	1	7.5	100	35	32	32	1					
12	Control	Gabarito MTE	6	2	4	pleasure	2	2	286	0	0	0	0	0	2.2	0	0	3	1,008	0.2	2	7.5	126	30	8	62	0	441.86				
11	Control	Gabarito MTD	5	0	5	pleasure	2	2	286	0	0	0	0	0	2.4	0	0	2	1,008	0.2	0	7.5	140	18	29	51	2	420.5				
10	Control	Encantada MTE	6	4	2	pleasure	1	3	335	0	0	0	0	0	1.6	4	3	3	1,010	0.6	0	7.5	100	6	20	72	2	541.57				
39	OAG	Lemmon MTD	36	9	27	show jumping	2	17	470	2	1	1	2	2	3.5	1	0	3	0.4	1.01	2	9	100	26	28	46	0	531.97	87.86	1327.77	747.26	8.15
40	OAG	Lemmon MTE	30	2	28	flat racing	2	17	470	0	0	1	0	1	2	1	0	2	0.4	1.01	2	9	100	2	14	84	0	559.43	47.91	1327.77	718.51	0.46
42	OAG	Milongueiro MTE	76	53	23	show jumping	2	16	499	0	0	1	1	2	7	1	0	3	1	1.01	2	9						488.34	42.20	1806.65	936.74	0.39
41	OAG	Milongueiro MTD	77	40	37	show jumping	2	16	499	1	2	1	4	4	4	1	0	3	0.6	1.01								613.22	31.35	1871.74	931.52	11.50
43	OAG	Neanderthal MTD	57	37	20	show jumping	2	15	455	0	0	0	0	0	3	1	1	4	0.8	1.01		9						627.92	34.54	1481.20	864.87	12.90
44	OAG	Neanderthal MTE	56	37	19	show jumping	2	15	455	0	0	0	0	0	4	4	0	2	1.0	1.01								517.73	26.11	1499.79	871.40	12.90
50	OAG	Xtreme MTE	47	13	34	show jumping	2	11	554	1	1	0	0	0	7	1	0	1	1.4	1.02	3	8						492.75	92.80	1439.35	850.50	0.70
49	OAG	Xtreme MTD	38	11	27	show jumping	2	11	554	0	2	0	0	0	8	1	0	2	0.6	1.01	2	8						607.34	87.86	1327.77	806.07	0.87
52	OAG	Zélia MTE	33	8	25	show jumping	1	17	430	0	1	0	2	2	2	4	0	2	0.3	1.01	3	9						392.85	109.36	1355.66	897.54	1.32
51	OAG	Zélia MTD	37	13	24	show jumping	1	17	430	1	0	0	0	3	6	1	1	3	0	1.01	3	8						569.15	71.46	1406.81	853.11	1.79
15	OAG	Best yet MTD	42	17	25	flat racing	2	4	496	1	0	0	0	0	8	1	0	3	1012	0.8	3	8	25	4	30	66	0	598.03	109.32	1239.43	653.83	2.48
16	OAG	Best yet MTE	48	13	35	flat racing	2	4	496	0	0	0	0	0	7	1	0	3	1012	1	2	8	20	14	41	45	0	507.64	86.02	1392.86	629.62	0.03
13	OAG	A todo vapor MTD	85	66	19	flat racing	2	5	505	1	0	0	0	1	8	2	2	3	1014	1.2	2	8	30	7	16	77	0	536.80	67.97	1569.53	978.56	1.71
14	OAG	A todo vapor MTE	91	65	26	flat racing	2	5	505	1	0	0	0	2	10	4	3	2	1020	2.4	0	9	150	20	10	69	1	870.66	67.97	1304.52	962.88	0.66
17	OAG	Caetano da Serra MTD	41	6	35	flat racing	2	6	475	1	0	0	1	2	5	3	2	10	1012	1	0	8	30					1026.65	140.40	1499.79	697.85	7.66
18	OAG	Caetano da Serra MTE	76	46	30	flat racing	2	6	475	1	0	0	1	1	5	3	2	2	1012	0.8	3	7	140	5	27	68	0	596.57	67.97	1499.79	702.25	3.02
19	OAG	Dreher MTD	59	21	38	flat racing	2	3	417	0	1	0	0	0	6	4	1	3	1010	0.6	3	8		3	12	85	0	666.55	52.78	1039.51	601.00	25.76
20	OAG	Dreher MTE	92	51	41	flat racing	2	3	417	1	1	0	0	0	5	4	1	2	1010	0.4	3	8		5	0	95	0	596.57	64.18	1206.89	471.14	0.66
21	OAG	End one MTD	46	8	38	flat racing	2	3	480	0	0	0	0	0	6.5	1	1	3	1012	1	3	7	100	38	2	60	0	786.10	93.97	1518.39	875.32	2.51
22	OAG	End one MTE	55	27	28	flat racing	2	3	480	0	0	0	0	0	6	3	1	2	1012	1	3	7		54	1	44	1	963.96	89.52	1499.79	851.80	71.82
23	OAG	Farawhip MTD	69	26	43	flat racing	2	6	490	1	0	2	3	3	6	4	3	2	1020	2.4	3	7	30	9	33	58	0	627.92	44.20	1578.83	696.75	61.07
24	OAG	Farawhip MTE	69	28	41	flat racing	2	6	490	2	0	2	0	1	9	4	2	2	1012	1	3	7	85	13	16	71	0	618.44	68.45	1430.05	696.75	55.02
25	OAG	Ishtevish MTD	62	18	44	flat racing	2	3	470	1	0	0	0	1	4.5	1	0	4	1010	0.6	0	8		0	0	100	0	637.39	35.62	1327.77	719.86	81.32
26	OAG	Ishtevish MTE	43	13	30	flat racing	2	3	470	2	1	0	0	2	5	1	0	5	1010	0.6	0	8	70	1	7	92	0	583.45	92.80	1369.61	701.15	15.82
27	OAG	Love is forever MTD	32	6	26	flat racing	1	5	470	0	0	0	0	0	2.5	1	0	3	1010	0.6	0	8						742.36	101.78	1462.60	949.81	0.73
28	OAG	Love is forever MTE	52	24	28	flat racing	1	5	470	2	0	2	0	1	10	2	1	3	1012	2.4	3	7						638.85	151.13	1643.92	945.89	67.12
29	OAG	Luke MTD	52	22	30	flat racing	2	4	439	0	0	0	1	2	10	1	0	4	1014	1.2	3	8	35	2	10	88	0	1020.82	96.62	1420.75	744.65	23.42
30	OAG	Luke MTE	33	14	19	flat racing	2	4	439	0	0	0	0	3	7	1	1	3	1012	1	3	8	30					559.15	57.64	839.59	744.65	1.32
31	OAG	Oscar winner MTD	47	12	35	flat racing	2	4	489	0	0	0	1	2	8	1	1	5	1012	0.8	0	8	100	0	2	98	0	721.95	119.67	1546.29	931.52	3.93
32	OAG	Oscar winner MTE	37	9	28	flat racing	2	4	489	0	0	0	0	0	8	2	1	5	1010	0.6	0	8		0	19	81	0	635.93	95.61	1178.99	943.28	6.30
37	OAG	Dommis MTD	42	11	31	show jumping	2	8	450	0	0	0	2	2	2	4	0	3	0.4	1.01	1	8	230	18	40	42	0	568.10	55.80	1469.57	945.89	3.60
48	OAG	Soledad MTE	87	52	35	show jumping	1	22	400	0	0	0	0	0	3	2	0	4	1.8	1.02	2	8	370	5	25	70	0	523.30	106.34	1981.46	921.28	0.66
45	OAG	Pinguim MTD	31	7	24	show jumping	2	12	400	1	0	0	0	0	6	4	1	3	0	1.01	3	9						448.68	31.80	1760.15	884.47	2.26
34	OAG	Alpargata MTE	62	43	19	show jumping	1	11	470	0	0	0	2	2	1	1	0	3	0.6	1.01	0	8	80	15	61	24	0	491.51	84.46	1378.91	905.38	12.90
33	OAG	Alpargata MTD	53	35	18	show jumping	1	11	470	0	0	0	0	1	2	1	0	3	0.6	1.01	0	8	100	20	10	70	0	591.23	115.33	1057.18	915.84	3.22
36	OAG	Dengosa MTE	52	36	16	show jumping	1	18	400	1	1	0	0	3	8	2	0	2	1.7	1.02	2	9						489.81	91.54	1802.00	847.88	3.60
35	OAG	Dengosa MTD	48	31	17	show jumping	1	18	400	0	1	0	0	0	8	1	0	2	0.3	1.01	2	9						525.07	66.73	1671.82	819.13	43.84

Análises – tabela sem dados faltantes

Data Integrity Check:

- Checking sample names - spaces will replaced with underscore, and special characters will be removed;
- Checking the class labels - at least three replicates are required in each class.
- The data (except class labels) must not contain non-numeric values.
- If the samples are paired, the pair labels must conform to the specified format.
- The presence of missing values or features with constant values (i.e. all zeros).

Data processing information:

Checking data content ...passed.

Samples are in rows and features in columns

The uploaded file is in comma separated values (.csv) format.

The uploaded data file contains 41 (samples) by 21 (compounds) data matrix.

Samples are not paired.

2 groups were detected in samples.

Only English letters, numbers, underscore, hyphen and forward slash (/) are allowed.

Other special characters or punctuations (if any) will be stripped off.

Non-numeric values were found and replaced by NA.

1 features with a constant or single value across samples were found and deleted.

A total of 6 (0.7%) missing values were detected.

By default, missing values will be replaced by 1/5 of min positive values of their corresponding variables

Click the **Proceed** button if you accept the default practice;

Or click the **Missing Values** button to use other methods.

Edit Groups | Missing Values | Proceed

17/02/2025

Sample normalization

- ( ) **None**
- ( ) **Sample-specific normalization** (i.e. weight, volume) Specify
- ( ) **Normalization by sum**
- ( ) **Normalization by median**
- ( ) **Normalization by a reference sample (PQN)** Specify
- ( ) **Normalization by a pooled sample from group (group PQN)** Specify
- ( ) **Normalization by reference feature** Specify
- ( ) **Quantile normalization** (suggested only for > 1000 features)

Data transformation

- ( ) **None**
- ( ) **Log transformation** (base 10)
- ( ) **Square root transformation** (square root of data values)
- ( ) **Cube root transformation** (cube root of data values)

Data scaling

- ( ) **None**
- ( ) **Mean centering** (mean-centered only)
- ( ) **Auto scaling** (mean-centered and divided by the standard deviation of each variable)
- ( ) **Pareto scaling** (mean-centered and divided by the square root of the standard deviation of each variable)
- ( ) **Range scaling** (mean-centered and divided by the range of each variable)

Normalize View Result Proceed

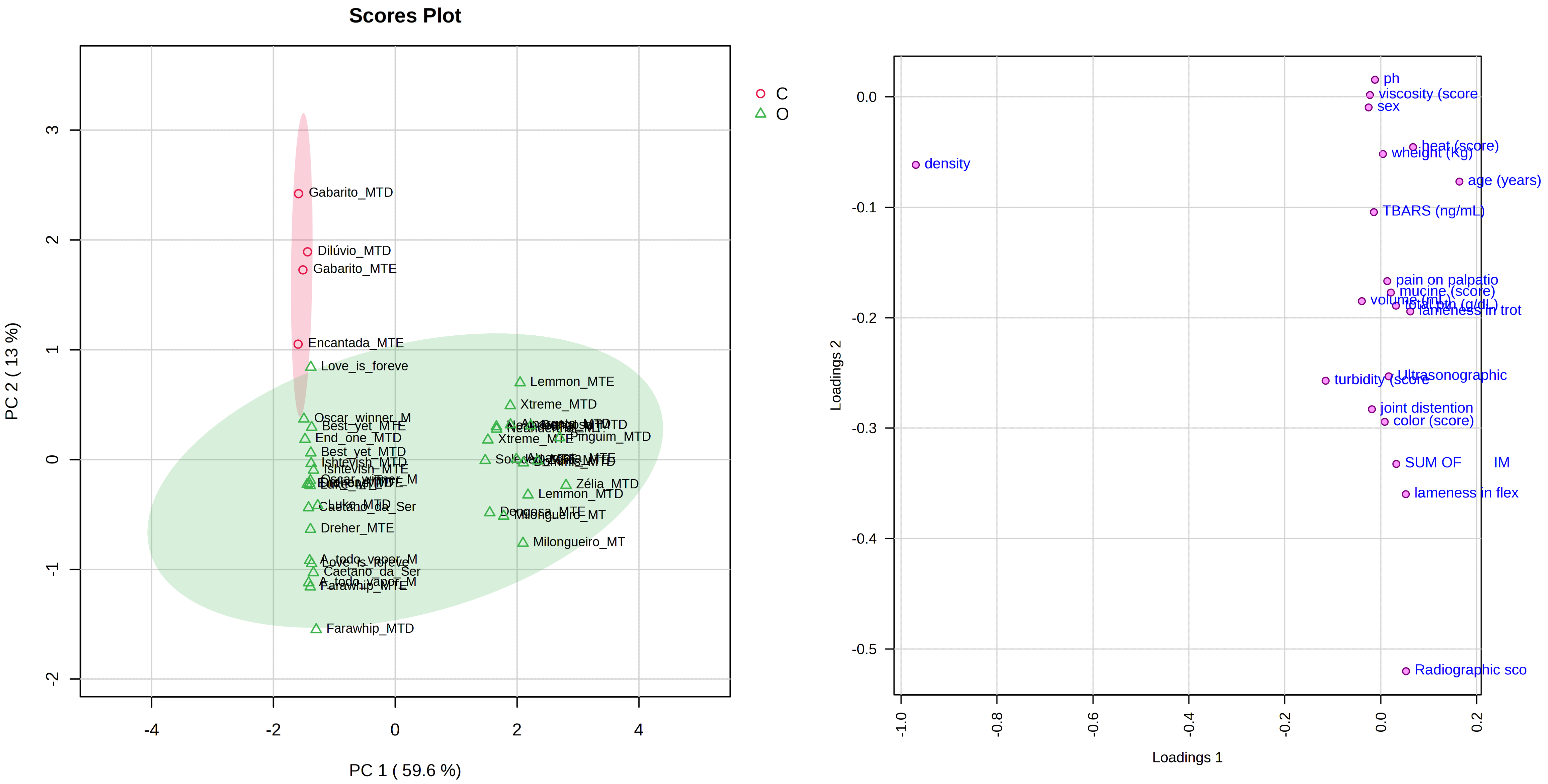
Scores Plot
PC 1 (59.6 %)
PC 2 (13 %)
C
O
Gabarito_MTD
Dilúvio_MTD
Gabarito_MTE
Encantada_MTE
Love_is_foreve
Lemmon_MTE
Xtreme_MTD
Oscar_winner_M
Best_yet_MTE
End_one_MTD
Best_yet_MTD
Xtreme_MTE
Pinguim_MTD
Zélia_MTD
Lemmon_MTD
Caetano_da_Ser
Dreher_MTE
Milongueiro_MT
Farawhip_MTE
Farawhip_MTD
density
ph
viscosity (score
sex
heat (score)
wheight (Kg)
age (years)
TBARS (ng/mL)
pain on palpatio
mucine (score)
volume (mL)
lameness in trot
turbidity (score
Ultrasonographic
joint distention
color (score)
SUM OF
IM
lameness in flex
Radiographic sco
Loadings 1
Loadings 2

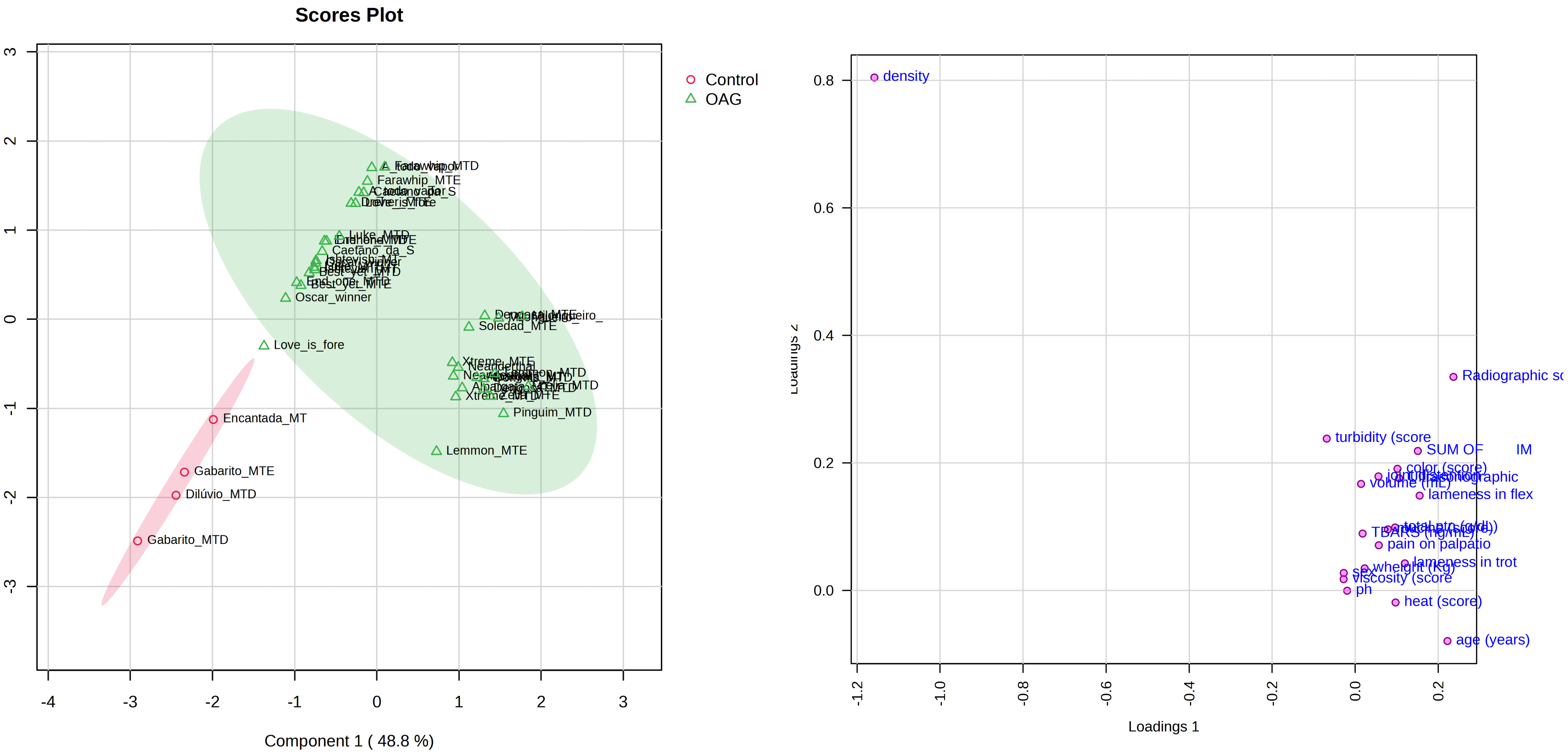

Scores Plot
Control
OAG
Component 1 (48.8 %)
Farawhip_MTE
Caetano_da_S
Oscar_winner
Soledad_MTE
Love_is_fore
Xtreme_MTE
Pinguim_MTD
Lemmon_MTE
Encantada_MT
Gabarito_MTE
Dilúvio_MTD
Gabarito_MTD
Loadings 1
density
Radiographic sc
turbidity (score
SUM OF IM
color (score)
volume (mL)
lameness in flex
TBARS (ng/mL)
pain on palpatio
lameness in trot
wheight (Kg)
viscosity (score
ph
heat (score)
age (years)

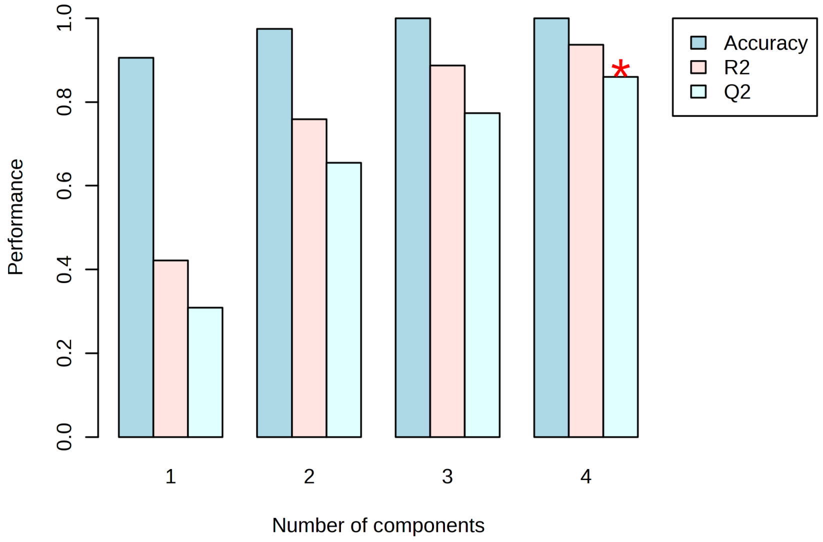

PLS-DA cross validation details:

Measure	1 comps	2 comps	3 comps	4 comps
Accuracy	0.90556	0.975	1.0	1.0
R2	0.42188	0.75849	0.88754	0.93679
Q2	0.30829	0.65468	0.77328	0.85993

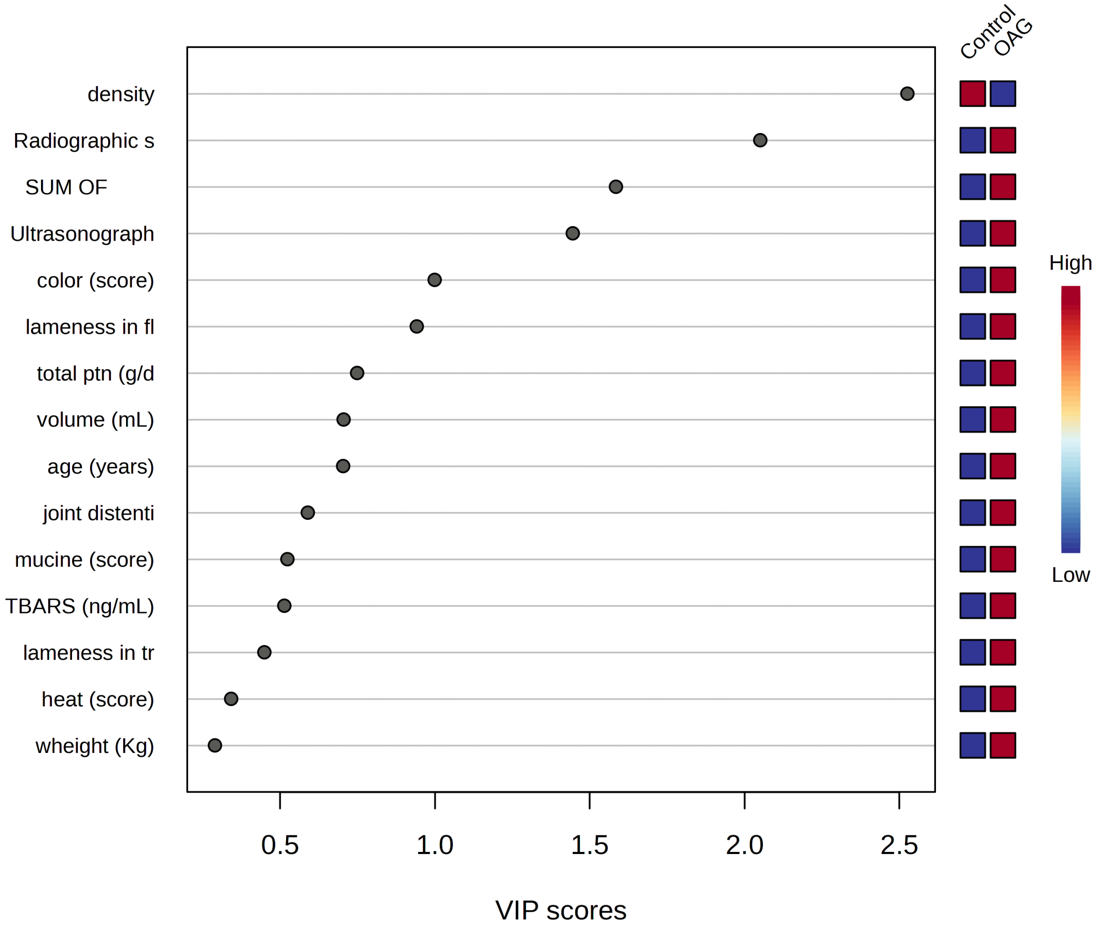

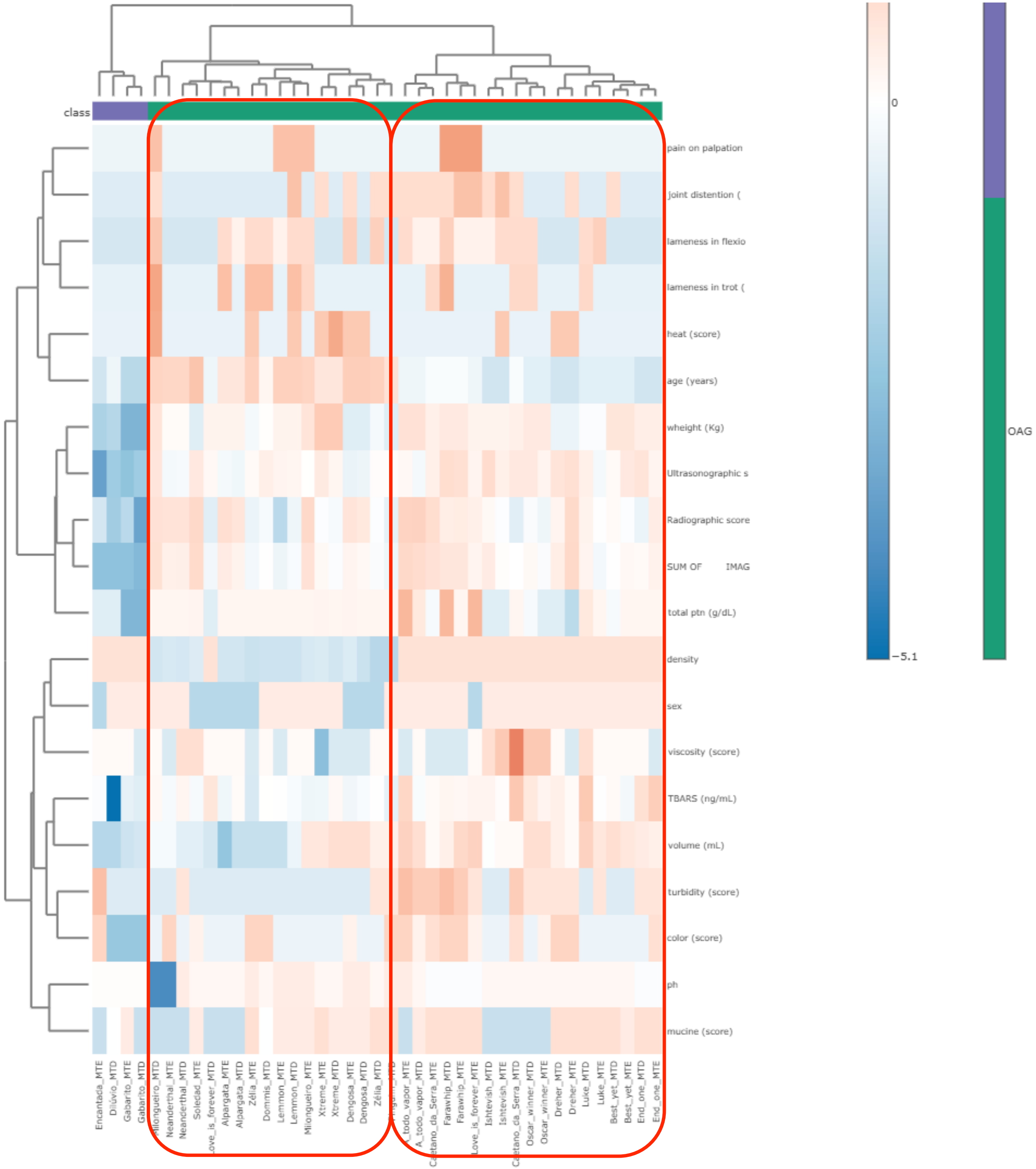

class
pain on palpation
joint distention (
lameness in flexio
lameness in trot (
heat (score)
age (years)
wheight (Kg)
Ultrasonographic s
Radiographic score
SUM OF IMAG
total ptn (g/dL)
density
sex
viscosity (score)
TBARS (ng/mL)
volume (mL)
turbidity (score)
color (score)
ph
mucine (score)
Encantada_MTE
Dilúvio_MTD
Gabarito_MTE
Gabarito_MTD
Milongueiro_MTD
Neanderthal_MTE
Neanderthal_MTD
Soledad_MTE
Alpargata_MTE
Alpargata_MTD
Zélia_MTE
Dommis_MTD
Lemmon_MTE
Lemmon_MTD
Milongueiro_MTE
Xtreme_MTE
Xtreme_MTD
Dengosa_MTE
Dengosa_MTD
Zélia_MTD
A_todo_vapor_MTE
A_todo_vapor_MTD
Farawhip_MTD
Farawhip_MTE
Ishtevish_MTD
Ishtevish_MTE
Oscar_winner_MTD
Oscar_winner_MTE
Dreher_MTD
Dreher_MTE
Luke_MTD
Luke_MTE
Best_yet_MTD
Best_yet_MTE
End_one_MTD
End_one_MTE
0
−5.1
OAG

	V	P.VALUE	[-LOG10(P)[	FDR
wheight (Kg)	0	0.001176	2.9295	0.007735
Ultrasonographic score	0	0.001216	2.9152	0.007735
SUM OF IMAGE SCORES	0	0.001231	2.9098	0.007735
Radiographic score	1.5	0.001547	2.8105	0.007735
total ptn (g/dL)	7	0.002737	2.5627	0.010948
volume (mL)	12	0.006617	2.1794	0.022055
TBARS (ng/mL)	14	0.008936	2.0489	0.02553
age (years)	17	0.012479	1.9038	0.031198

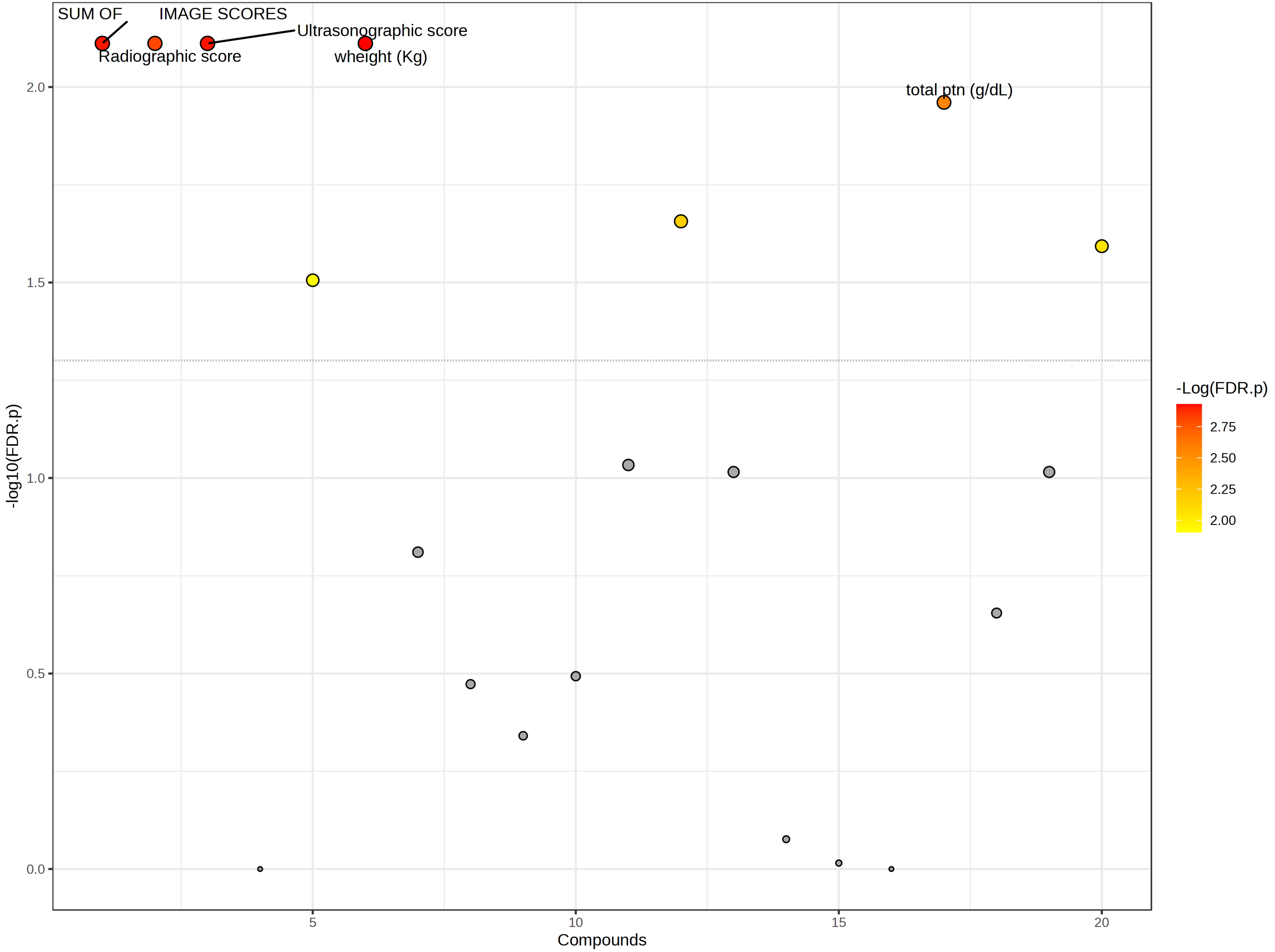

SUM OF
IMAGE SCORES
Ultrasonographic score
Radiographic score
wheight (Kg)
total ptn (g/dL)
-log10(FDR.p)
2.0
1.5
1.0
0.5
0.0
5
10
15
20
Compounds
-Log(FDR.p)
2.75
2.50
2.25
2.00

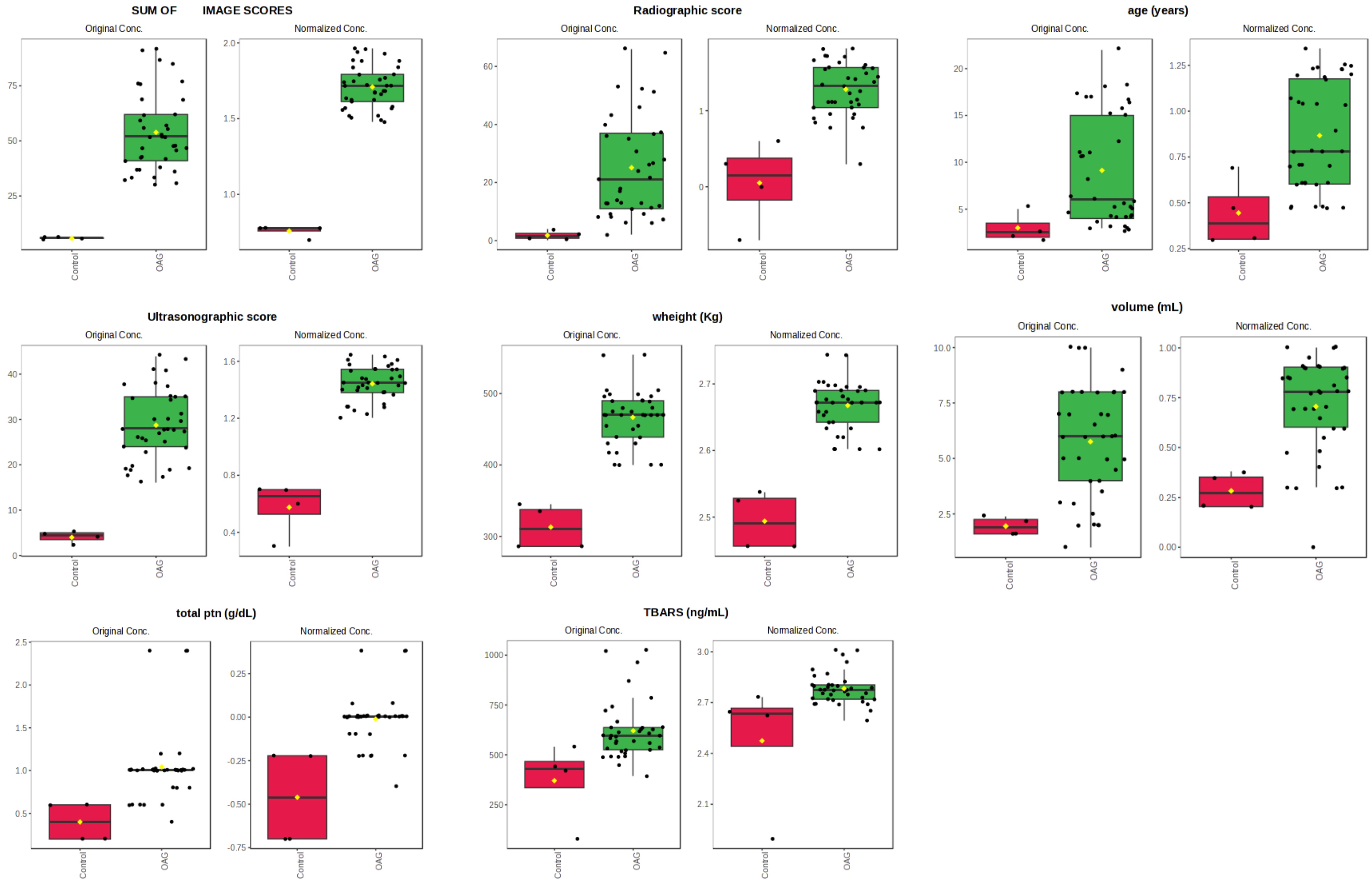
SUM OF IMAGE SCORES
Original Conc.
Normalized Conc.
Radiographic score
Original Conc.
Normalized Conc.
age (years)
Original Conc.
Normalized Conc.
Ultrasonographic score
Original Conc.
Normalized Conc.
wheight (Kg)
Original Conc.
Normalized Conc.
volume (mL)
Original Conc.
Normalized Conc.
total ptn (g/dL)
Original Conc.
Normalized Conc.
TBARS (ng/mL)
Original Conc.
Normalized Conc.
Control
OAG
