# Supplementary material for: Ultrasonographic Evidence of Synovitis Correlates with Synovial Citrate and TBARS in Equine Osteoarthritis
Source: Vet Sci. 2026 Jan 31;13(2):140. doi: 10.3390/vetsci13020140 (PMC12945042; doi:10.3390/vetsci13020140)
Supplement: Supplementary file 1 [file vetsci-13-00140-s001.zip › 2025 Supplementary files/2025 4 Statistics of C2C.pdf]

Resultados

Estatística Descritiva

Estatística Descritiva

	Grupo	C2C
N	CG	8
	OAG	37
Omisso	CG	0
	OAG	0
Média	CG	47.4
	OAG	78.0
Erro-padrão da média	CG	7.53
	OAG	5.05
W de Shapiro-Wilk	CG	0.972
	OAG	0.972
p Shapiro-Wilk	CG	0.910
	OAG	0.464

Teste t para amostras independentes

Teste t para amostras independentes

		Estatística	gl	p
C2C	t de Student	-2.67	43.0	0.011

Nota. H_a $\mu_{CG} \neq \mu_{OAG}$

Referências

[1] The jamovi project (2022). *jamovi*. (Version 2.3) [Computer Software]. Retrieved from https://www.jamovi.org.

[2] R Core Team (2021). *R: A Language and environment for statistical computing*. (Version 4.1) [Computer software]. Retrieved from https://cran.r-project.org. (R packages retrieved from MRAN snapshot 2022-01-01).
