# Supplementary material for: Ultrasonographic Evidence of Synovitis Correlates with Synovial Citrate and TBARS in Equine Osteoarthritis
Source: Vet Sci. 2026 Jan 31;13(2):140. doi: 10.3390/vetsci13020140 (PMC12945042; doi:10.3390/vetsci13020140)
Supplement: Supplementary file 1 [file vetsci-13-00140-s001.zip › 2025 Supplementary files/2025 5 Statistics of ultrasonographic scores.pdf]

Resultados

Estatística Descritiva

Estatística Descritiva

	grupo	N	Mediana	Mínimo	Máximo	Percentis	
						25th	75th
cápsula articular	Controle	12	0.000	0	3	0.00	1.250
	Sinovite	40	2.000	0	4	0.00	3.000
prega sinovial	Controle	12	0.000	0	1	0.00	1.000
	Sinovite	40	3.000	0	4	2.00	4.000
TEDC	Controle	12	0.000	0	0	0.00	0.000
	Sinovite	40	0.000	0	3	0.00	1.000
Sup. MCIII	Controle	12	0.000	0	0	0.00	0.000
	Sinovite	40	1.000	0	3	0.00	2.000
Sup. FP	Controle	12	0.000	0	0	0.00	0.000
	Sinovite	40	1.000	0	3	0.00	2.000
L. SUS RL	Controle	12	0.000	0	1	0.00	1.000
	Sinovite	40	2.000	0	5	1.00	3.000
L. col curto	Controle	12	1.000	0	1	0.00	1.000
	Sinovite	40	0.000	0	4	0.00	1.250
L. col longo	Controle	12	0.000	0	0	0.00	0.000
	Sinovite	40	0.000	0	4	0.00	1.000
Sup. ses lat	Controle	12	0.000	0	1	0.00	0.000
	Sinovite	40	1.500	0	3	1.00	2.000
LS recesso	Controle	12	0.000	0	0	0.00	0.000
	Sinovite	40	2.000	0	2	0.00	2.000
L SUS RM	Controle	12	0.000	0	2	0.00	1.000
	Sinovite	40	3.000	0	5	2.00	4.000
L.col curto	Controle	12	0.000	0	1	0.00	1.000
	Sinovite	40	0.500	0	4	0.00	2.250
L. col longo (2)	Controle	12	0.000	0	0	0.00	0.000
	Sinovite	40	0.000	0	3	0.00	1.250
Sup. ses med	Controle	12	0.000	0	1	0.00	0.000
	Sinovite	40	2.000	0	2	1.00	2.000
LS recesso (2)	Controle	12	0.000	0	0	0.00	0.000
	Sinovite	40	1.000	0	2	0.00	2.000
LAPP	Controle	12	0.000	0	0	0.00	0.000
	Sinovite	40	0.000	0	1	0.00	0.000
TFDS	Controle	12	0.000	0	0	0.00	0.000
	Sinovite	40	0.000	0	2	0.00	1.000
TFDP	Controle	12	0.000	0	0	0.00	0.000
	Sinovite	40	0.000	0	3	0.00	1.000
L ses obl lat	Controle	12	0.000	0	2	0.00	1.000
	Sinovite	40	2.000	0	4	1.00	3.000
L ses obl med	Controle	12	0.000	0	1	0.00	0.250
	Sinovite	40	3.000	0	4	2.00	3.000
L ses reto	Controle	12	0.000	0	1	0.00	0.000

Estatística Descritiva

						Percentis	
	grupo	N	Mediana	Mínimo	Máximo	25th	75th
	Sinovite	40	1.000	0	4	1.00	2.000

Gráficos

cápsula articular

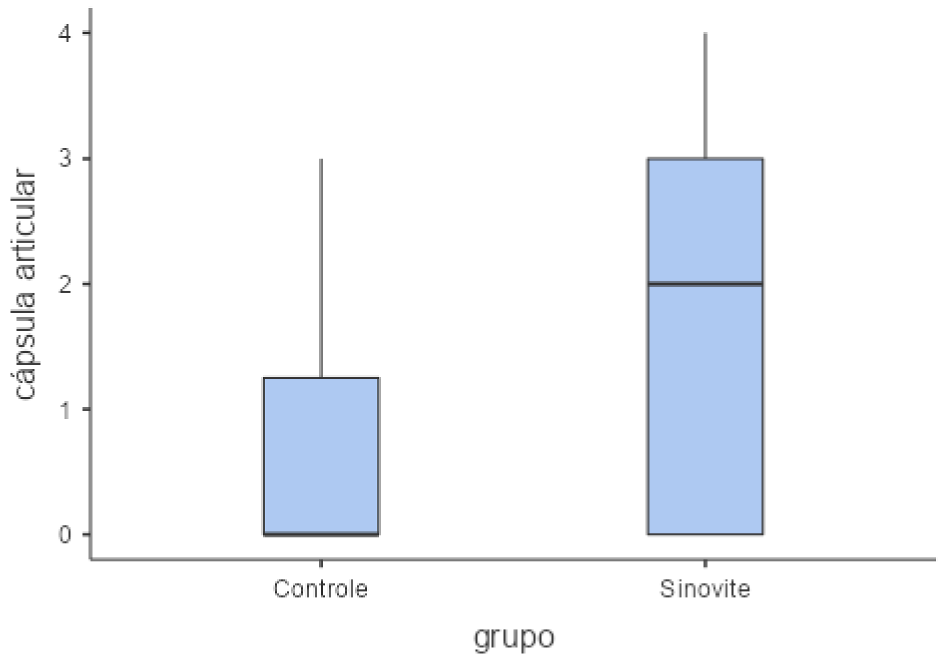

prega sinovial

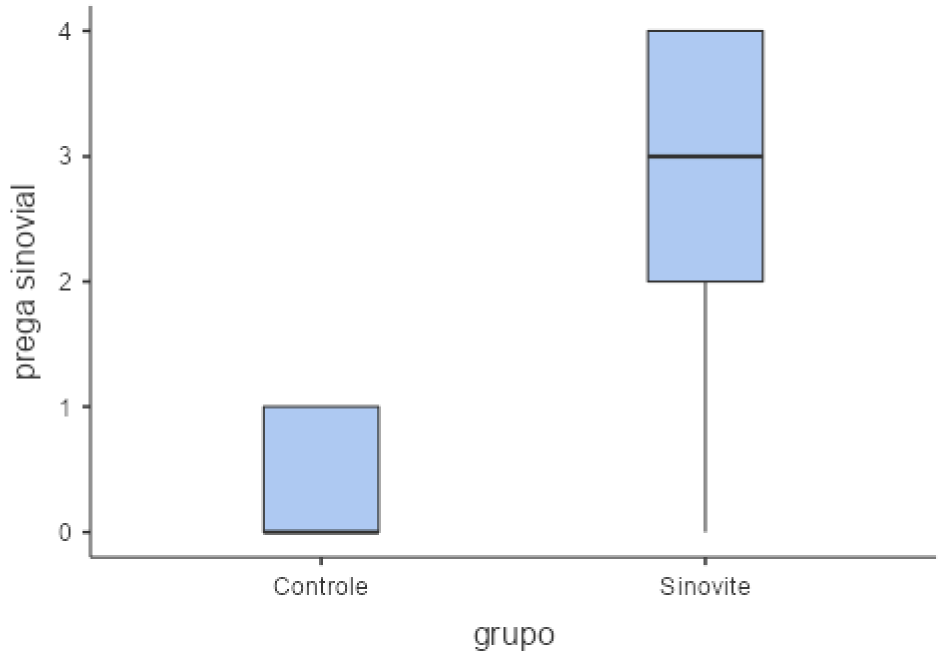

TEDC

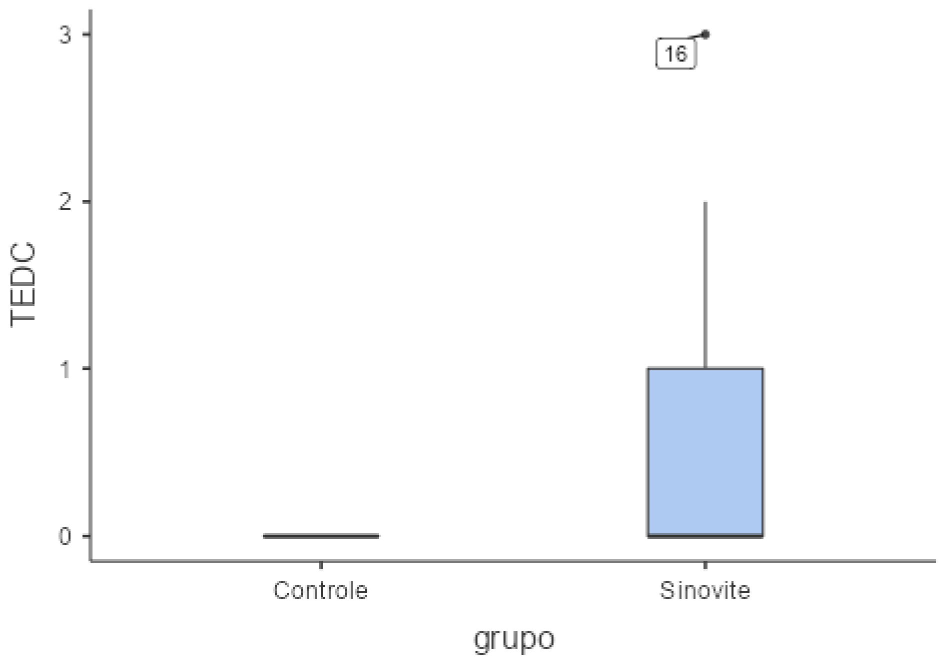

Sup. MCIII

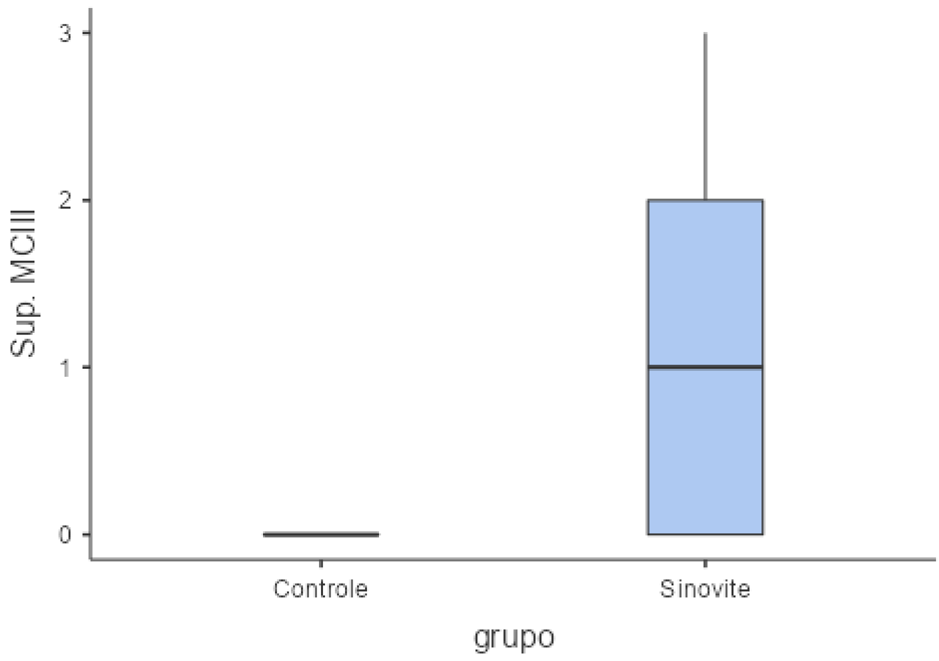

Sup. FP

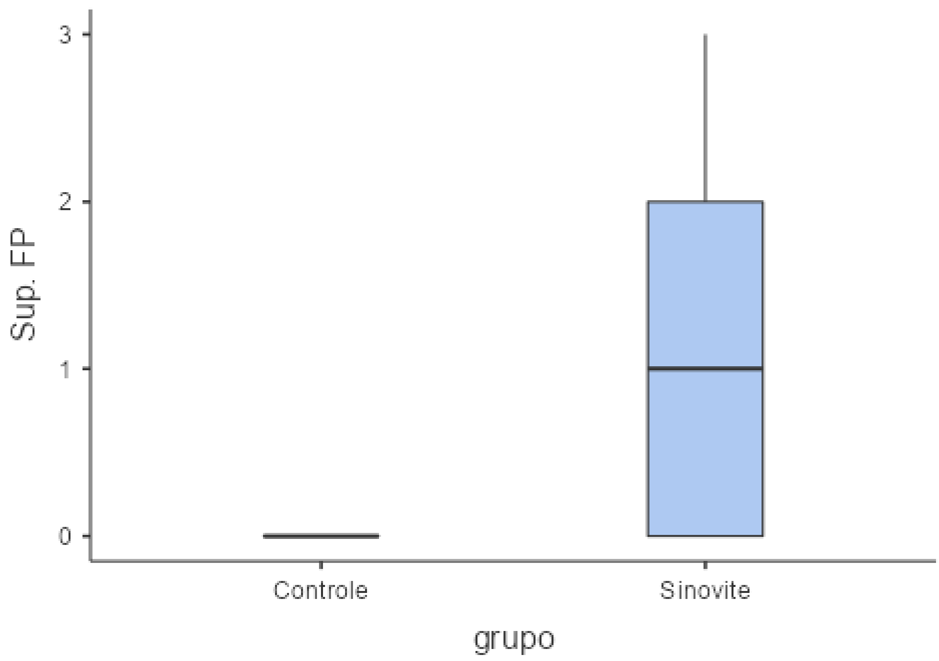

L. SUS RL

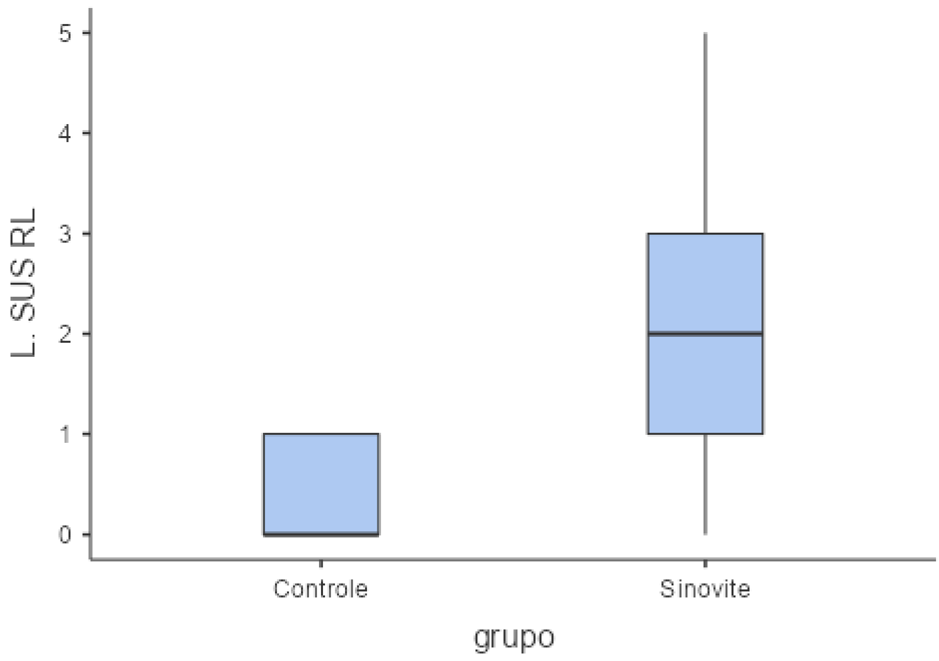

L. col curto

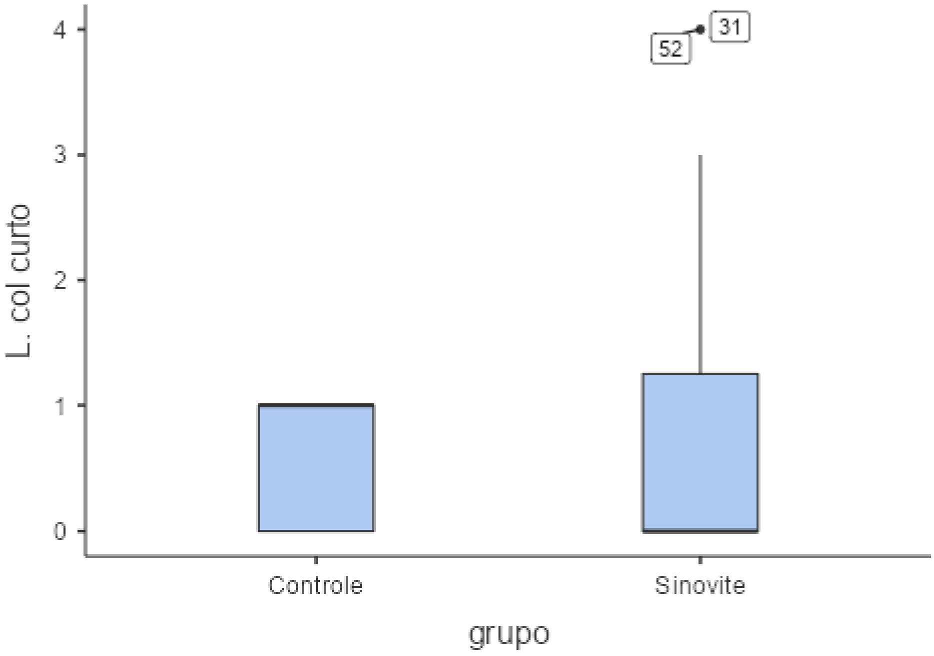

L. col longo

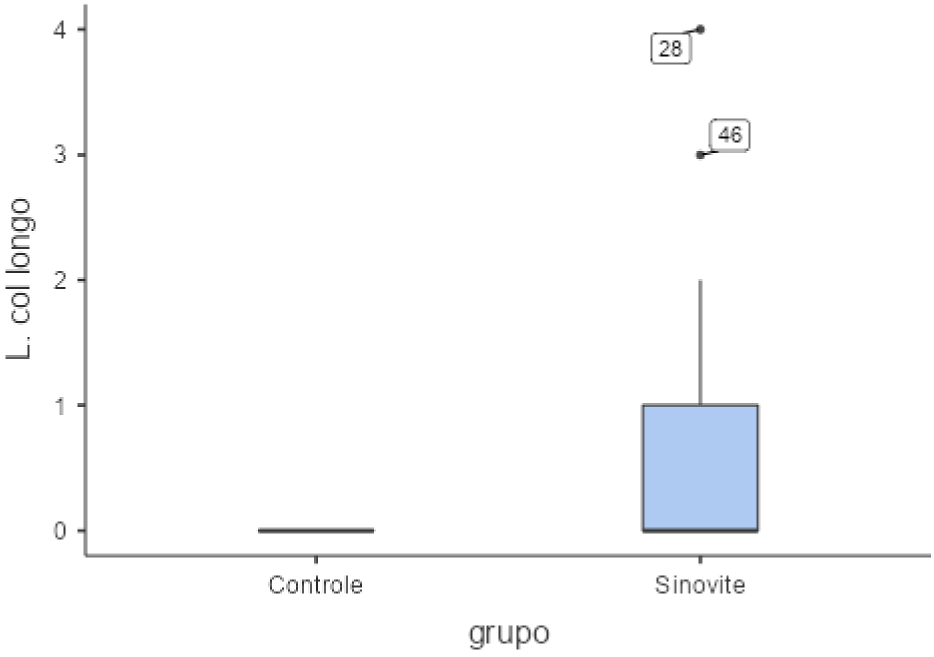

Sup. ses lat

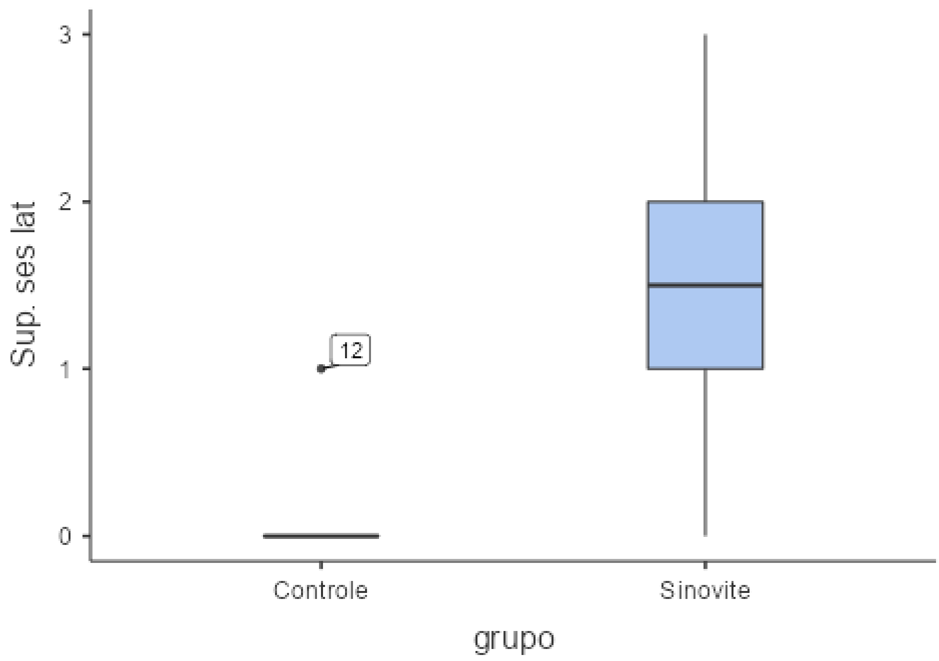

LS recesso

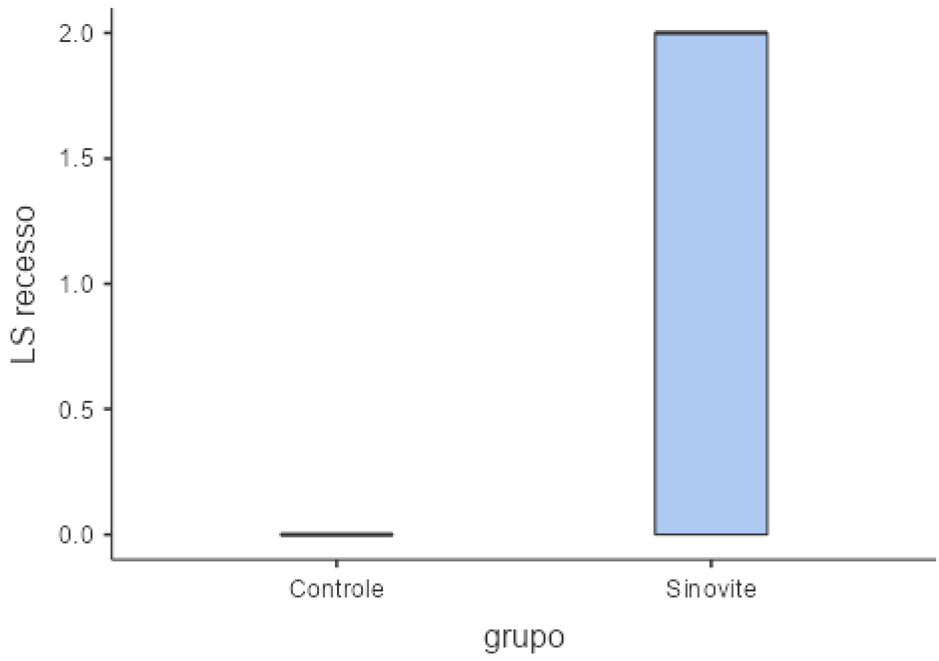

L SUS RM

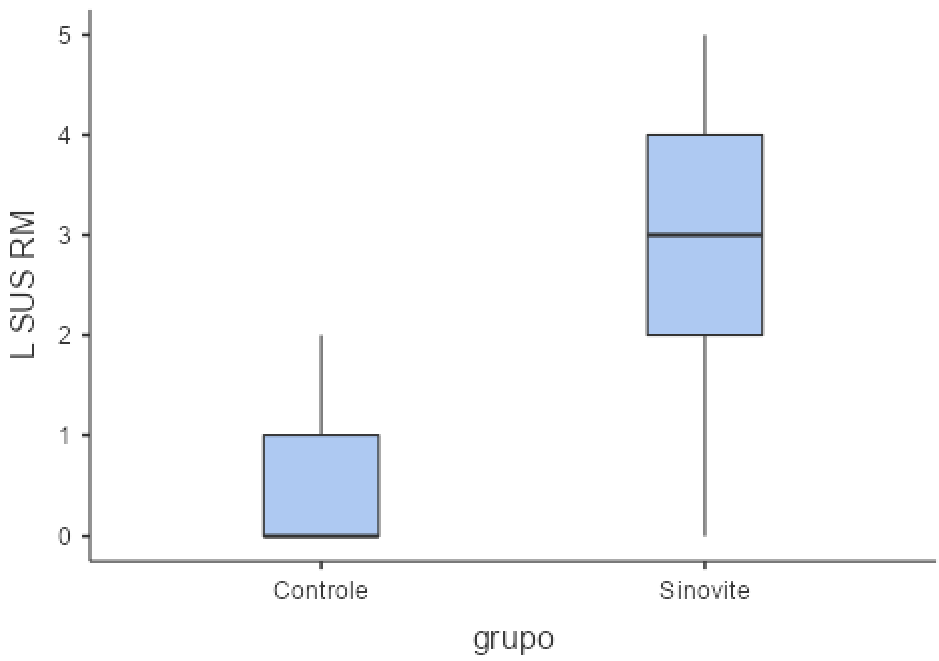

L.col curto

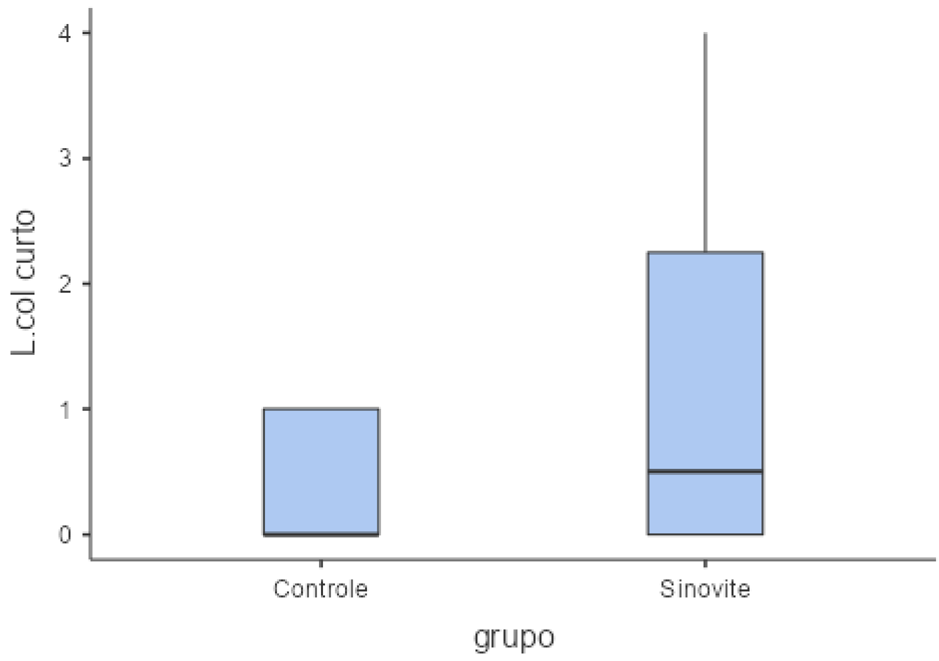

L. col longo (2)

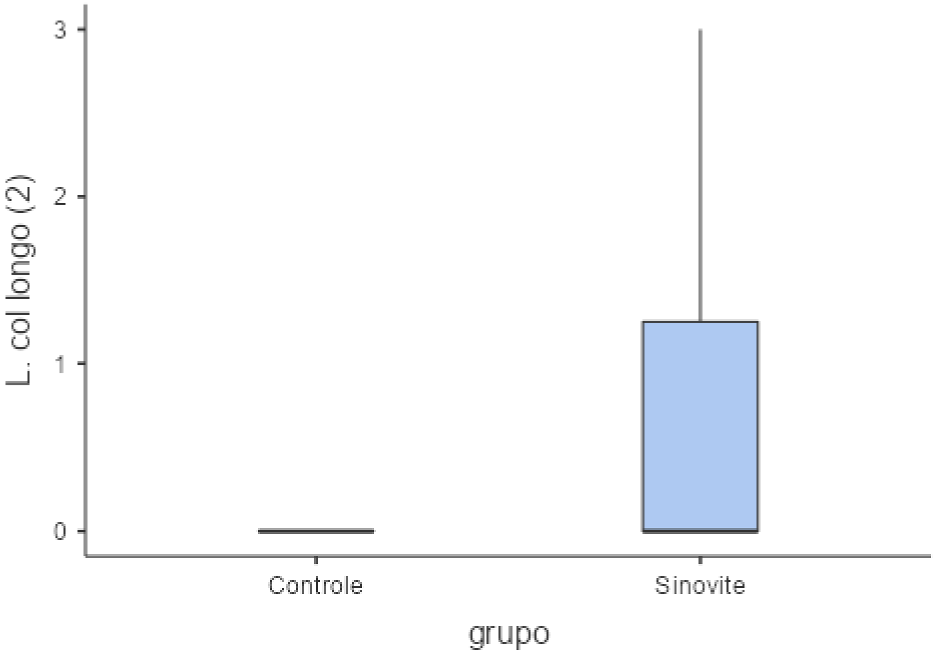

Sup. ses med

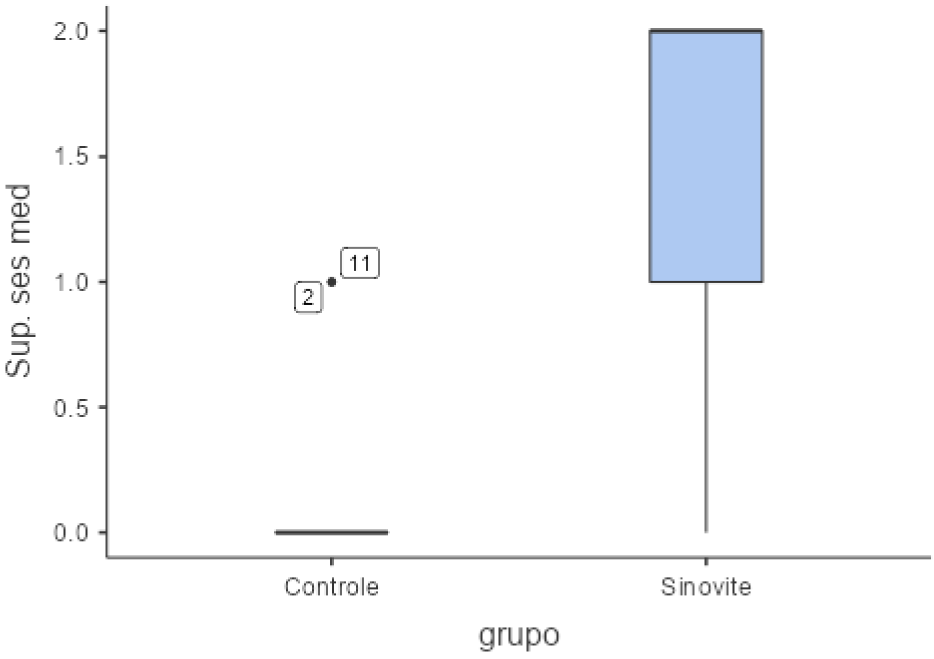

LS recesso (2)

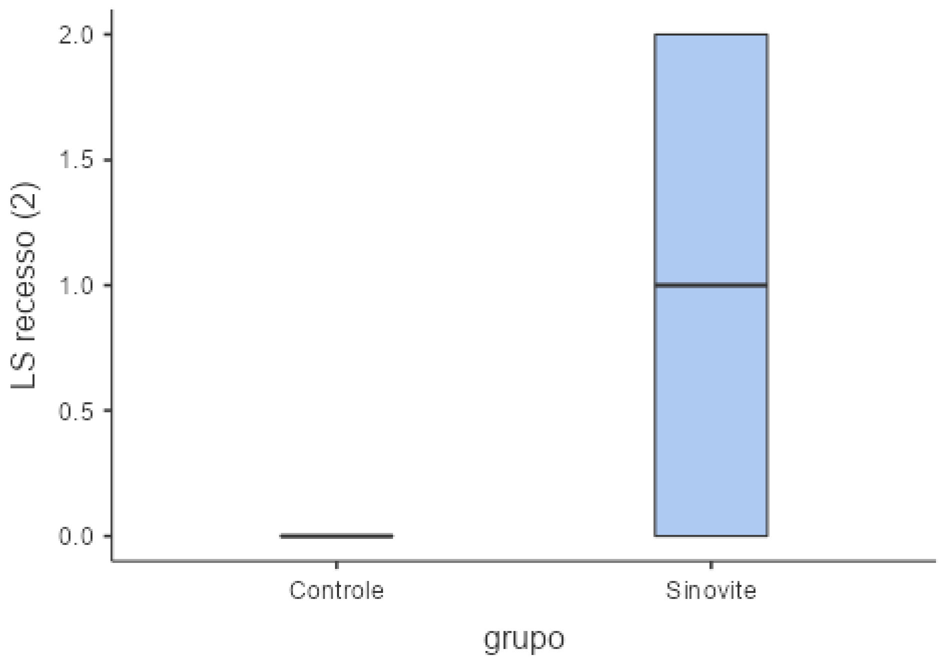
2.0
1.5
1.0
0.5
0.0
LS recesso (2)
Controle
Sinovite
grupo

LAPP

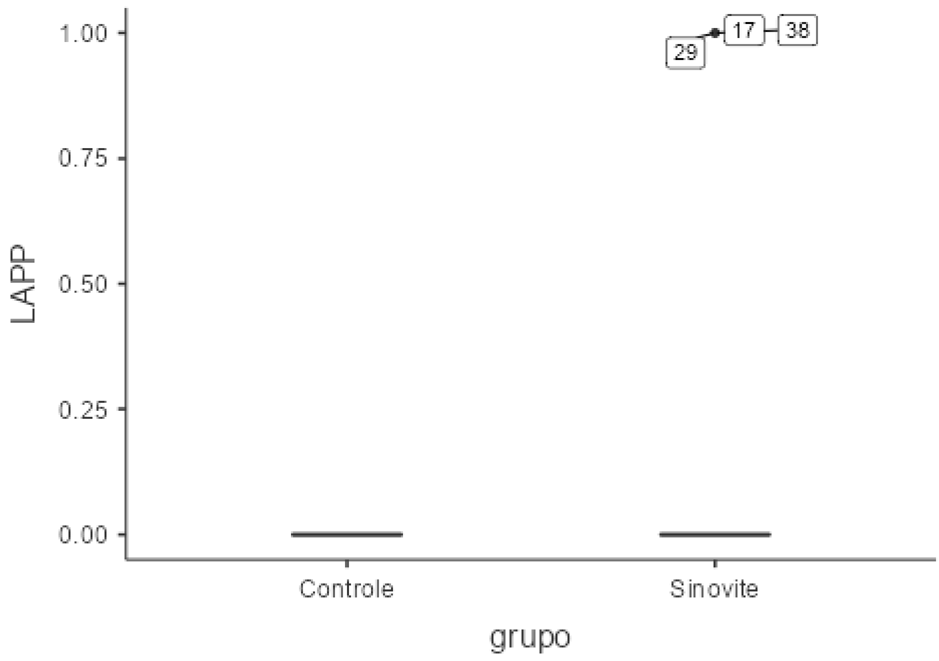
1.00
0.75
0.50
0.25
0.00
LAPP
29
17
38
Controle
Sinovite
grupo

TFDS

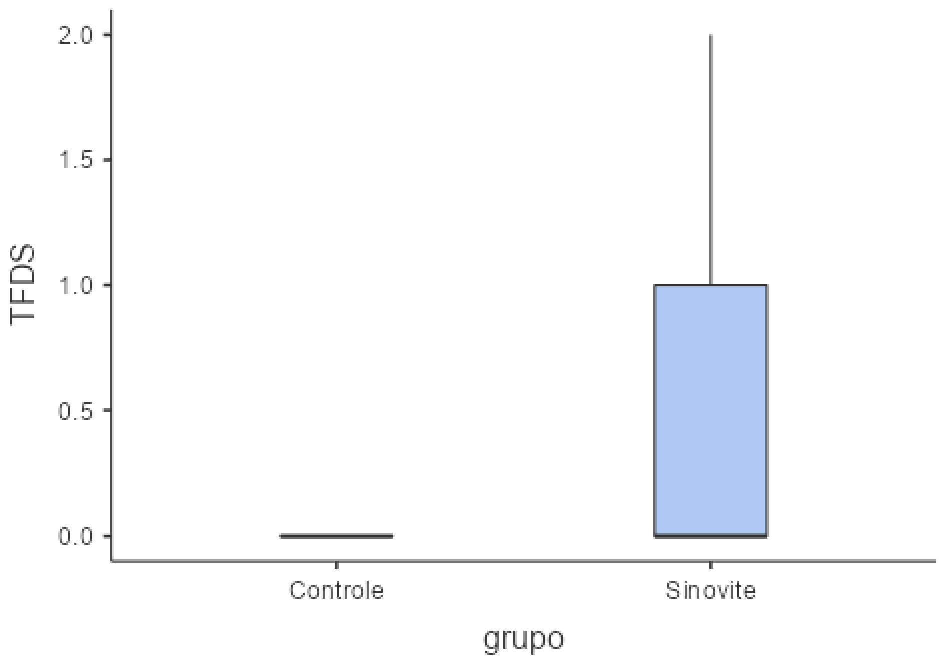

TFDP

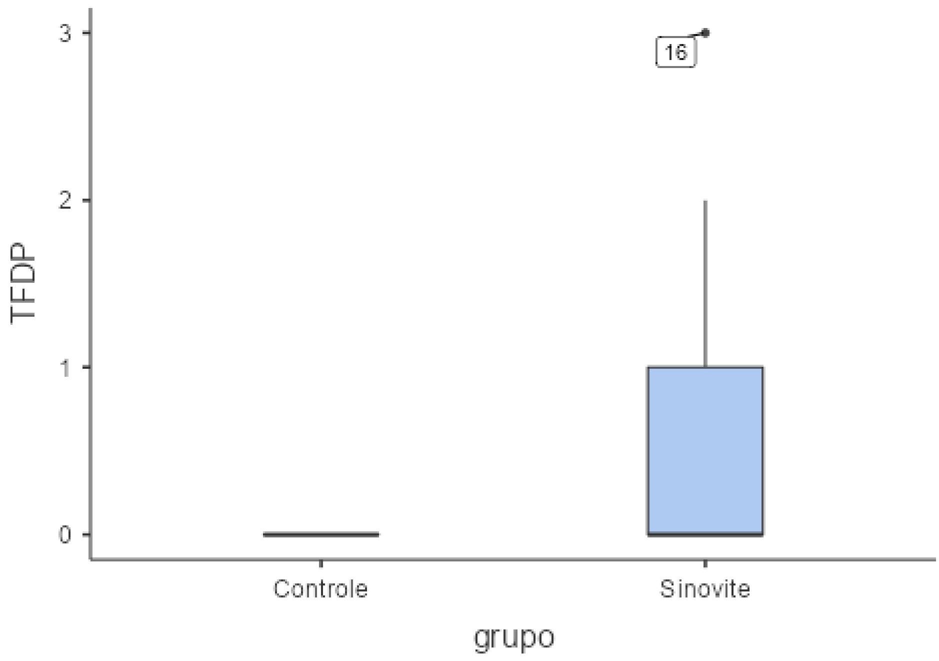

L ses obl lat

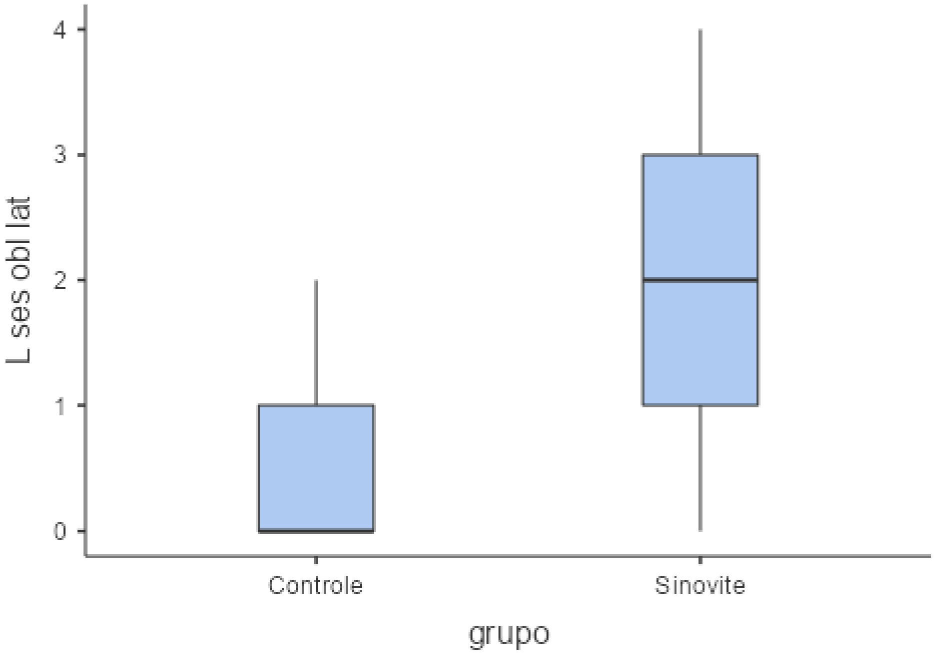

L ses obl med

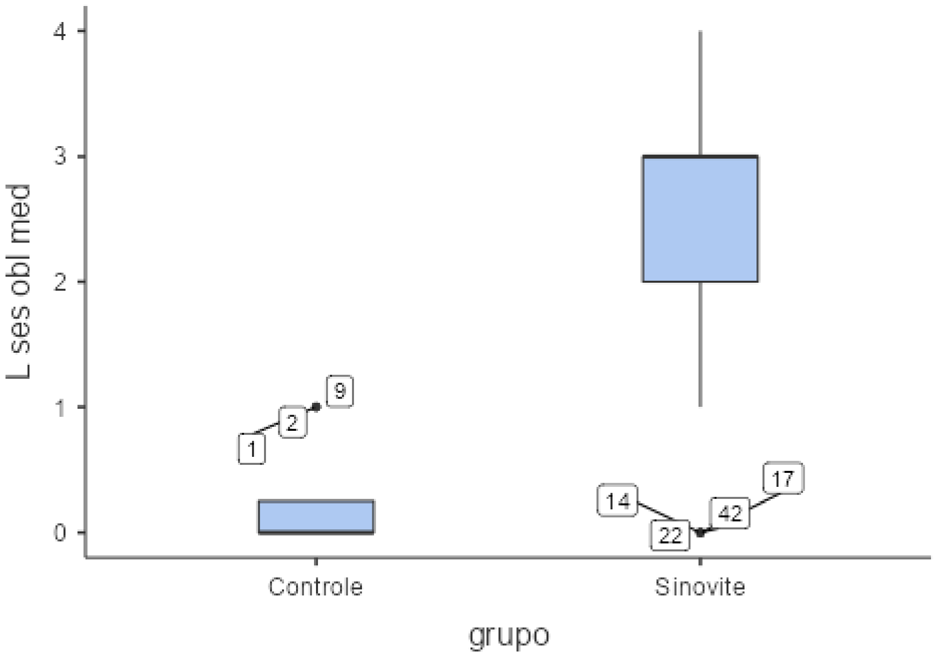

L ses reto

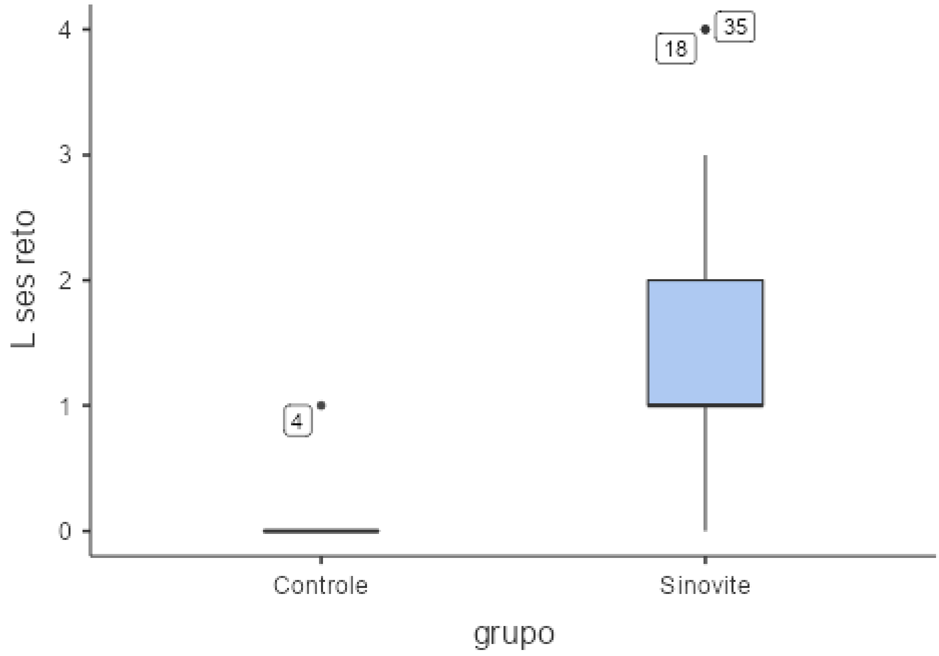

ANOVA a um fator (não-paramétrica)

Kruskal-Wallis

	χ^2	gl	p
cápsula articular	4.9554	1	0.026
prega sinovial	24.1950	1	< .001
TEDC	6.0188	1	0.014
Sup. MCIII	16.1291	1	< .001
Sup. FP	16.3370	1	< .001
L. SUS RL	12.4476	1	< .001
L. col curto	0.0804	1	0.777
L. col longo	7.1602	1	0.007
Sup. ses lat	22.4255	1	< .001
LS recesso	15.9690	1	< .001
L SUS RM	19.1666	1	< .001
L.col curto	1.6818	1	0.195
L. col longo (2)	6.5360	1	0.011
Sup. ses med	25.6096	1	< .001
LS recesso (2)	11.1714	1	< .001
LAPP	0.9367	1	0.333
TFDS	5.5032	1	0.019
TFDP	6.5633	1	0.010
L ses obl lat	15.3328	1	< .001
L ses obl med	20.8930	1	< .001
L ses reto	17.9082	1	< .001

Comparações múltiplas Dwass-Steel-Critchlow-Fligner

Comparações múltiplas - cápsula articular

		W	p
Controle	Sinovite	3.15	0.026

Comparações múltiplas - prega sinovial

		W	p
Controle	Sinovite	6.96	< .001

Comparações múltiplas - TEDC

		W	p
Controle	Sinovite	3.47	0.014

Comparações múltiplas - Sup. MCIII

		W	p
Controle	Sinovite	5.68	< .001

Comparações múltiplas - Sup. FP

		W	p
Controle	Sinovite	5.72	< .001

Comparações múltiplas - L. SUS RL

		W	p
Controle	Sinovite	4.99	< .001

Comparações múltiplas - L. col curto

		W	p
Controle	Sinovite	-0.401	0.777

Comparações múltiplas - L. col longo

		W	p
Controle	Sinovite	3.78	0.007

Comparações múltiplas - Sup. ses lat

		W	p
Controle	Sinovite	6.70	< .001

Comparações múltiplas - LS recesso

		W	p
Controle	Sinovite	5.65	< .001

Comparações múltiplas - L SUS RM

		W	p
Controle	Sinovite	6.19	< .001

Comparações múltiplas - L.col curto

		W	p
Controle	Sinovite	1.83	0.195

Comparações múltiplas - L. col longo (2)

		W	p
Controle	Sinovite	3.62	0.011

Comparações múltiplas - Sup. ses med

		W	p
Controle	Sinovite	7.16	< .001

Comparações múltiplas - LS recesso (2)

		W	p
Controle	Sinovite	4.73	< .001

Comparações múltiplas - LAPP

		W	p
Controle	Sinovite	1.37	0.333

Comparações múltiplas - TFDS

		W	p
Controle	Sinovite	3.32	0.019

Comparações múltiplas - TFDP

		W	p
Controle	Sinovite	3.62	0.010

Comparações múltiplas - L ses obl lat

		W	p
Controle	Sinovite	5.54	< .001

Comparações múltiplas - L ses obl med

		W	p
Controle	Sinovite	6.46	< .001

Comparações múltiplas - L ses reto

		W	p
Controle	Sinovite	5.98	< .001

Referências

[1] The jamovi project (2022). *jamovi*. (Version 2.3) [Computer Software]. Retrieved from https://www.jamovi.org.

[2] R Core Team (2021). *R: A Language and environment for statistical computing*. (Version 4.1) [Computer software]. Retrieved from https://cran.r-project.org. (R packages retrieved from MRAN snapshot 2022-01-01).
