# Supplementary material for: Ultrasonographic Evidence of Synovitis Correlates with Synovial Citrate and TBARS in Equine Osteoarthritis
Source: Vet Sci. 2026 Jan 31;13(2):140. doi: 10.3390/vetsci13020140 (PMC12945042; doi:10.3390/vetsci13020140)
Supplement: Supplementary file 1 [file vetsci-13-00140-s001.zip › 2025 Supplementary files/2025 6 Statistics of radiographic scores.pdf]

Resultados

Estatística Descritiva

Estatística Descritiva

	Grupo	esclerose do osso subcondral
Mediana	CONTROL	0.00
	OSTEOARTHRITIS	1.00
25º percentil	CONTROL	0.00
	OSTEOARTHRITIS	1.00
75º percentil	CONTROL	0.00
	OSTEOARTHRITIS	1.00

ANOVA a um fator (não-paramétrica)

Kruskal-Wallis

	χ^2	gl	p
esclerose do osso subcondral	20.2	1	< .001

Estatística Descritiva

Estatística Descritiva

	Grupo	lise do osso subcondral
Mediana	CONTROL	0.500
	OSTEOARTHRITIS	1.00
25º percentil	CONTROL	0.00
	OSTEOARTHRITIS	1.00
75º percentil	CONTROL	1.00
	OSTEOARTHRITIS	2.00

ANOVA a um fator (não-paramétrica)

Kruskal-Wallis

	χ^2	gl	p
lise do osso subcondral	16.1	1	< .001

Estatística Descritiva

Estatística Descritiva

	Grupo	espessamento e radiopacidade dos tecidos moles
Mediana	CONTROL	0.00
	OSTEOARTHRITIS	1.00
25º percentil	CONTROL	0.00
	OSTEOARTHRITIS	1.00
75º percentil	CONTROL	0.00
	OSTEOARTHRITIS	3.00

ANOVA a um fator (não-paramétrica)

Kruskal-Wallis

	χ^2	gl	p
espessamento e radiopacidade dos tecidos moles	21.5	1	< .001

Estatística Descritiva

Estatística Descritiva

	Grupo	estreitamento do espaço articular
Mediana	CONTROL	0.00
	OSTEOARTHRITIS	0.00
25º percentil	CONTROL	0.00
	OSTEOARTHRITIS	0.00
75º percentil	CONTROL	0.00
	OSTEOARTHRITIS	0.00

ANOVA a um fator (não-paramétrica)

Kruskal-Wallis

	χ^2	gl	p
estreitamento do espaço articular	3.20	1	0.074

Estatística Descritiva

Estatística Descritiva

	Grupo	numero de osteófitos/entesófitos
Mediana	CONTROL	0.00
	OSTEOARTHRITIS	1.00
25º percentil	CONTROL	0.00
	OSTEOARTHRITIS	1.00
75º percentil	CONTROL	0.00
	OSTEOARTHRITIS	3.00

ANOVA a um fator (não-paramétrica)

Kruskal-Wallis

	χ^2	gl	p
numero de osteófitos/entesófitos	13.6	1	< .001

Estatística Descritiva

Estatística Descritiva

	Grupo	tamanho de osteófitos/entesófitos
Mediana	CONTROL	0.00
	OSTEOARTHRITIS	1.00
25º percentil	CONTROL	0.00
	OSTEOARTHRITIS	1.00
75º percentil	CONTROL	0.00
	OSTEOARTHRITIS	3.00

ANOVA a um fator (não-paramétrica)

Kruskal-Wallis

	χ^2	gl	p
tamanho de osteófitos/entesófitos	13.7	1	< .001

Estatística Descritiva

Estatística Descritiva

	Grupo	número de fragmentos osteocondrais
Mediana	CONTROL	0.00
	OSTEOARTHRITIS	0.00
25º percentil	CONTROL	0.00
	OSTEOARTHRITIS	0.00
75º percentil	CONTROL	0.00
	OSTEOARTHRITIS	1.00

ANOVA a um fator (não-paramétrica)

Kruskal-Wallis

	χ^2	gl	p
número de fragmentos osteocondrais	7.73	1	0.005

Estatística Descritiva

Estatística Descritiva

	Grupo	tamanho dos fragmentos osteocondrais
Mediana	CONTROL	0.00
	OSTEOARTHRITIS	0.00
25º percentil	CONTROL	0.00
	OSTEOARTHRITIS	0.00
75º percentil	CONTROL	0.00
	OSTEOARTHRITIS	1.25

ANOVA a um fator (não-paramétrica)

Kruskal-Wallis

	χ^2	gl	p
tamanho dos fragmentos osteocondrais	7.73	1	0.005

Estatística Descritiva

Estatística Descritiva

	Grupo	soma do escore da articulação
Mediana	CONTROL	1.00
	OSTEOARTHRITIS	6.50
25º percentil	CONTROL	0.00
	OSTEOARTHRITIS	4.75
75º percentil	CONTROL	1.00
	OSTEOARTHRITIS	15.0

ANOVA a um fator (não-paramétrica)

Kruskal-Wallis

	χ^2	gl	p
soma do escore da articulação	25.6	1	< .001

Referências

[1] The jamovi project (2022). *jamovi*. (Version 2.3) [Computer Software]. Retrieved from https://www.jamovi.org.

[2] R Core Team (2021). *R: A Language and environment for statistical computing*. (Version 4.1) [Computer software]. Retrieved from https://cran.r-project.org. (R packages retrieved from MRAN snapshot 2022-01-01).
