# Supplementary material for: Ultrasonographic Evidence of Synovitis Correlates with Synovial Citrate and TBARS in Equine Osteoarthritis
Source: Vet Sci. 2026 Jan 31;13(2):140. doi: 10.3390/vetsci13020140 (PMC12945042; doi:10.3390/vetsci13020140)
Supplement: Supplementary file 1 [file vetsci-13-00140-s001.zip › 2025 Supplementary files/2025 9 Statistics cytology - monocytes % .pdf]

Resultados

Estatística Descritiva

Estatística Descritiva

	Grupo	Monócitos
N	CG	8
	OAG	24
Omisso	CG	0
	OAG	0
Média	CG	76.5
	OAG	48.0
Erro-padrão da média	CG	16.5
	OAG	11.9
W de Shapiro-Wilk	CG	0.923
	OAG	0.764
p Shapiro-Wilk	CG	0.458
	OAG	< .001

Teste t para amostras independentes

Teste t para amostras independentes

		Estatística	p
Monócitos	U de Mann-Whitney	57.0	0.092

Nota. H_a $\mu_{CG} \neq \mu_{OAG}$

Pressupostos

Teste à Normalidade (Shapiro-Wilk)

	W	p
Monócitos	0.830	< .001

Nota. Um p-value pequeno sugere a violação do pressuposto da normalidade

Referências

[1] The jamovi project (2022). *jamovi*. (Version 2.3) [Computer Software]. Retrieved from https://www.jamovi.org.

[2] R Core Team (2021). *R: A Language and environment for statistical computing*. (Version 4.1) [Computer software]. Retrieved from https://cran.r-project.org. (R packages retrieved from MRAN snapshot 2022-01-01).
