# Supplementary material for: Ultrasonographic Evidence of Synovitis Correlates with Synovial Citrate and TBARS in Equine Osteoarthritis
Source: Vet Sci. 2026 Jan 31;13(2):140. doi: 10.3390/vetsci13020140 (PMC12945042; doi:10.3390/vetsci13020140)
Supplement: Supplementary file 1 [file vetsci-13-00140-s001.zip › 2026 13 multivariate analysis .pdf]

Multivariate analysis

19/01/2026

Gilson Costa

Data Integrity Check:

- Checking sample names - spaces will replaced with underscore, and special characters will be removed;
- Checking the class labels - at least three replicates are required in each class.
- The data (except class labels) must not contain non-numeric values.
- If the samples are paired, the pair labels must conform to the specified format.
- The presence of missing values or features with constant values (i.e. all zeros).

Data processing information:

Checking data content ...passed.

Samples are in rows and features in columns

The uploaded file is in comma separated values (.csv) format.

The uploaded data file contains 48 (samples) by 33 (compounds) data matrix.

Samples are not paired.

2 groups were detected in samples.

Only English letters, numbers, underscore, hyphen and forward slash (/) are allowed.

Other special characters or punctuations (if any) will be stripped off.

All data values are numeric.

A total of 150 (9.5%) missing values were detected.

Edit Groups

Proceed

- **Low-quality filter** - exclude heavily contaminated features (based on BLANK samples), or features contain too many missing values.
- **Low-repeatability filter** - exclude features showing high percent RSD based on QC replicates (suggested threshold: 20% for LC-MS and 30% for GC-MS);
- **Low-variance filter** - exclude features that are near-constant across the experiment conditions - detected using different statistical measures of variance;
- **Low-abundance filter** - exclude features are of very small values (close to baseline) - these variables can be detected using mean or median (useful when BLANK samples are unavailable).

**Low-quality filter**	☐ No BLANK detected<br>☑ Enable missing-value exclusion	**For blank substraction:**<br>Sample/blank ratio threshold: 10<br>**For missing value exclusion:**<br>Remove features with > 50 %<br>Use group-wise threshold ☐ View
**Low-repeatability filter**	☐ No QC samples detected	RSDs greater than: 20% View
**Low-variance filter**	◉ Interquantile range (IQR)<br>○ Standard deviation (SD)<br>○ Median absolute deviation (MAD)<br>○ Relative standard deviation (RSD = SD/mean)<br>○ Non-parametric relative standard deviation (MAD/median)	Percentage to filter out:<br>5%
**Low-abundance filter**	◉ Mean intensity value<br>○ Median intensity value	Percentage to filter out:<br>0%

Submit Proceed

Missing value estimation:

Too many missing values will cause difficulties for downstream analysis. There are three general categories for missing value imputation.

- **Left-censored data estimation**: it assumes that the true value of an observation is known to be below a certain threshold (the LoD), but its exact value is unknown. The default method LoD (limit of detection) will replace all the missing values with 1/5 of the minimum positive values of individual features as proxies of their detection limits. Alternatively, the Quantile Regression Imputation of Left-Censored data (QRILC) method which imputes missing data by modeling each feature's low tail as log-normal and sampling replacements.
- **Univariate statistical methods**: this approach replaces missing values by mean/median/min based on the non-missing values of the feature;
- **Multivariate statistical methods**: these methods leverage correlations between features or samples to estimate the missing entries, including k-nearest neighbours based on similar features - KNN (feature-wise), k-nearest neighbours based on similar samples - KNN (sample-wise), probabilistic PCA (PPCA), Bayesian PCA (BPCA) method, singular value decomposition (SVD) (ref), and *missForest* based on random forest(ref). Note k is set to 10 for KNN; the max missing values is set to 30000 for missForest due to its computational intensive nature.

Missing Value Summary | Missing Value Heatmap

After filtering step, there are 150 missing values (9.5% of the data). Kruskal-Wallis test: **p** = **0.0108**.

When the percentage of missing values or average abundance differs significantly across experimental groups, it suggests there are systematic differences in samples across groups ('batch effect'). **Sample-wise normalization** such as normalize by mean, median, sum or probabilistic quotient normalization (PQR) are recommended before transformation or scaling.

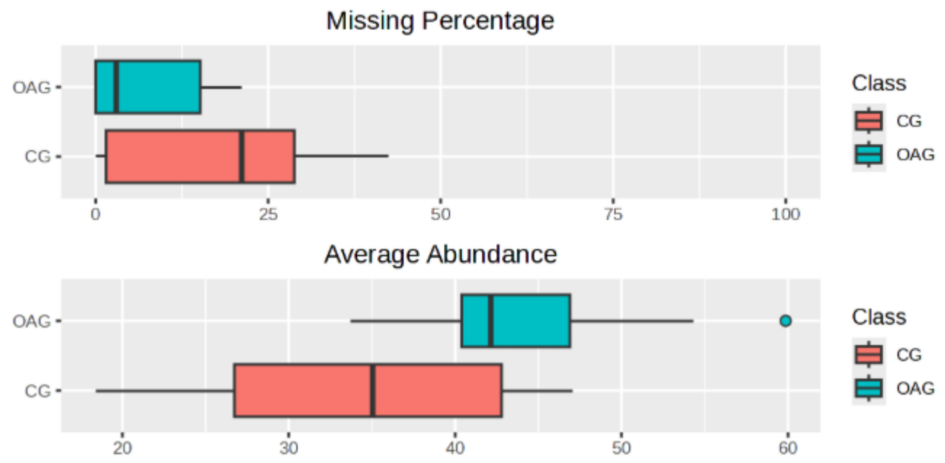

- Normalização pelo log10

PCA

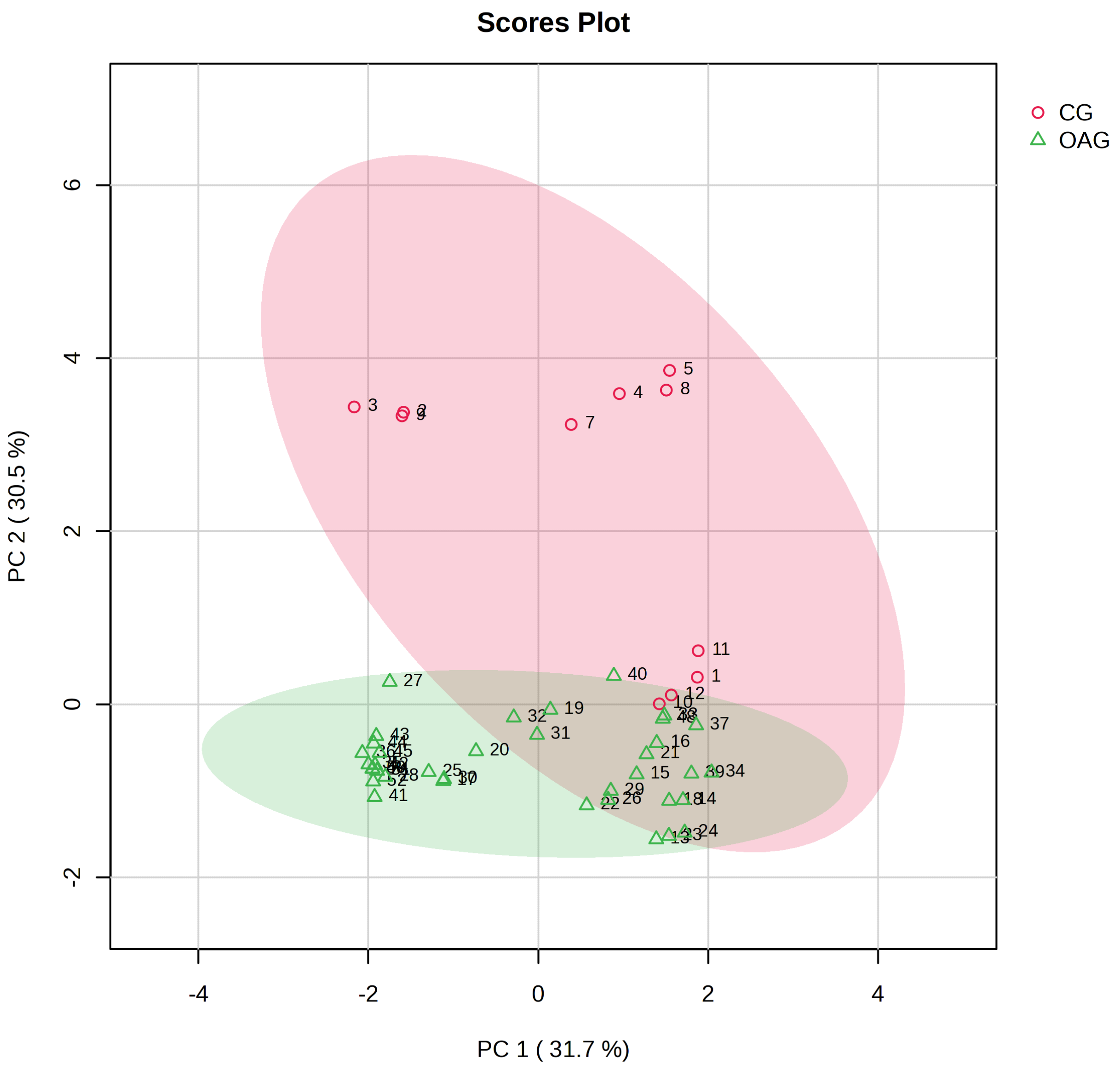

Scores Plot
CG
OAG
PC 1 (31.7 %)
PC 2 (30.5 %)
-4
-2
0
2
4
6

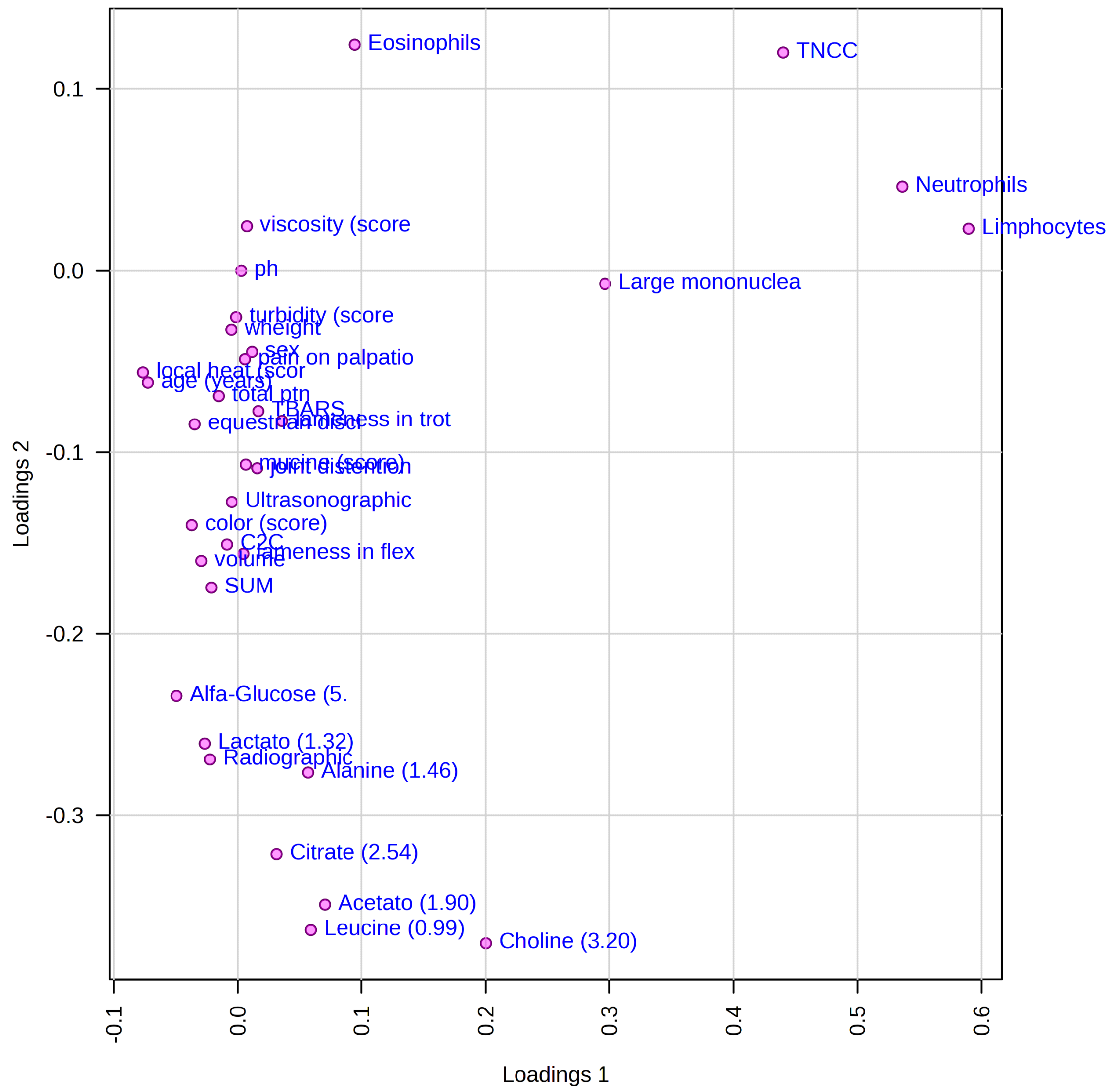

Eosinophils
TNCC
Neutrophils
Limphocytes
viscosity (score
ph
Large mononuclea
turbidity (score
wheight
sex
pain on palpatio
local heat (scor
age (years)
total ptn
TBARS
lameness in trot
equestrian discl
mucine (score)
joint distention
Ultrasonographic
color (score)
C2C
lameness in flex
volume
SUM
Alfa-Glucose (5.
Lactato (1.32)
Radiographic
Alanine (1.46)
Citrate (2.54)
Acetato (1.90)
Leucine (0.99)
Choline (3.20)
Loadings 1
Loadings 2
-0.1
0.0
0.1
0.2
0.3
0.4
0.5
0.6
-0.2
-0.3

PLSDA – Geração de modelo biológico

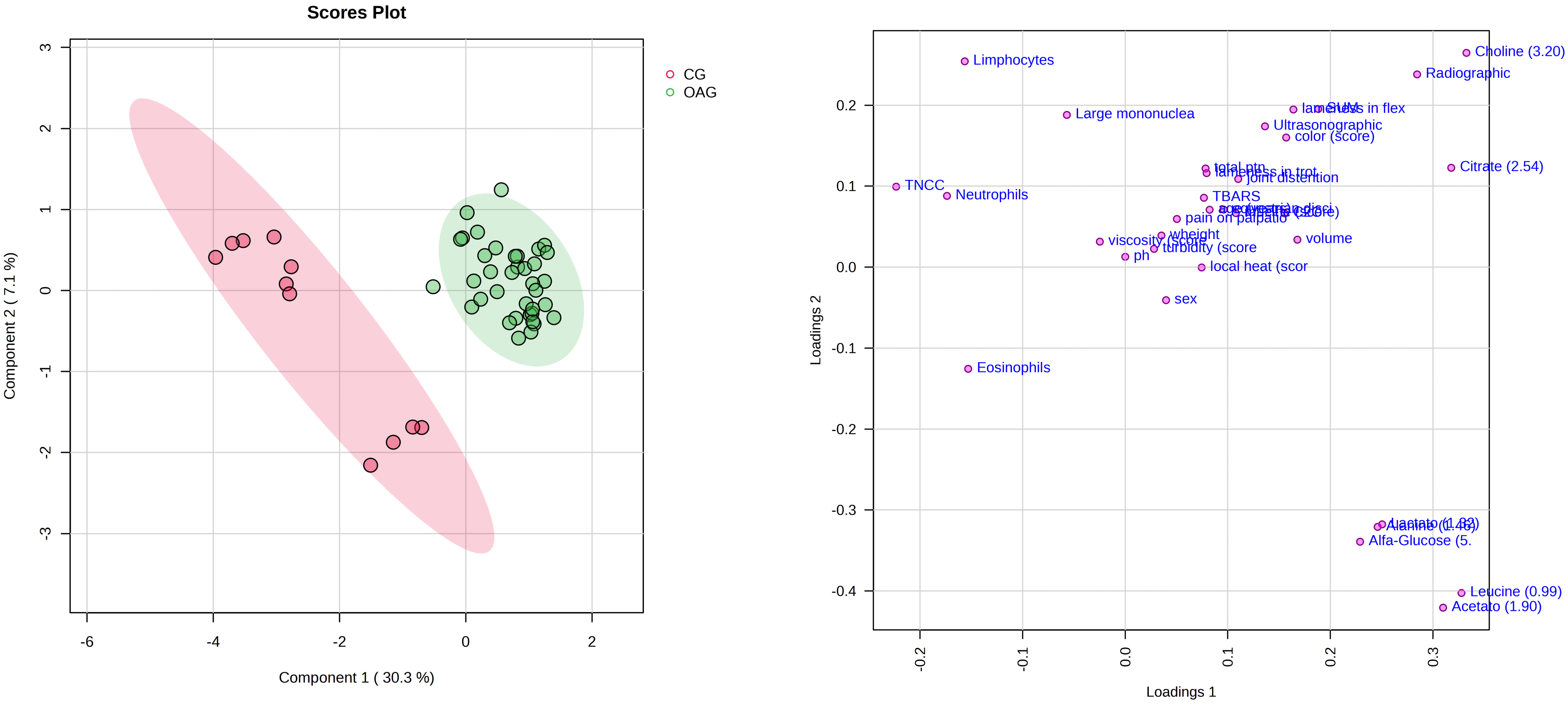
Scores Plot
CG
OAG
Component 1 (30.3 %)
Component 2 (7.1 %)
Limphocytes
Choline (3.20)
Radiographic
Large mononuclea
Ultrasonographic
color (score)
Citrate (2.54)
joint distention
TNCC
Neutrophils
TBARS
pain on palpatio
wheight
viscosity (score
volume
turbidity (score
ph
local heat (scor
sex
Eosinophils
Alfa-Glucose (5.
Leucine (0.99)
Acetato (1.90)
Loadings 1
Loadings 2

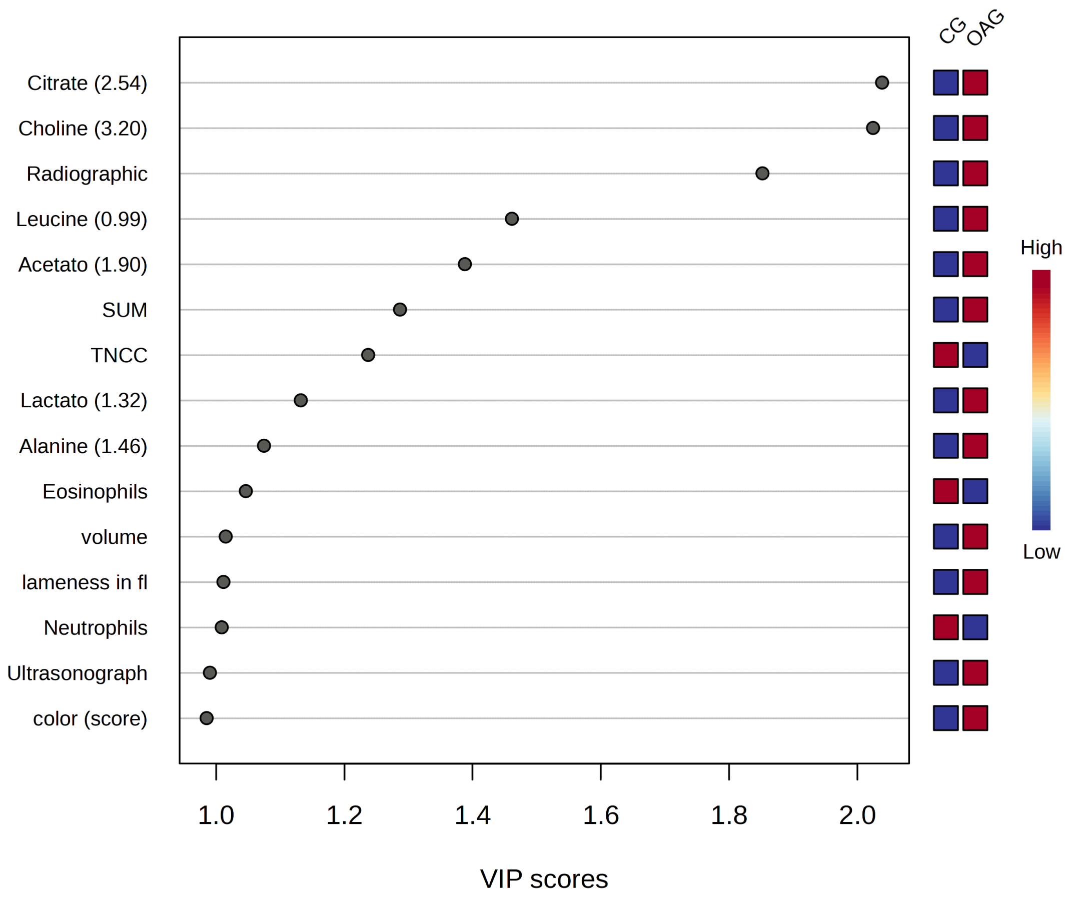

PLS-DA cross validation details:

Measure	1 comps	2 comps	3 comps	4 comps	5 comps
Accuracy	0.91556	0.95778	1.0	1.0	1.0
R2	0.79439	0.91413	0.92517	0.94404	0.96372
Q2	0.72424	0.82124	0.88713	0.89223	0.87429
