# Supplementary figures and images for: Ultrasonographic Evidence of Synovitis Correlates with Synovial Citrate and TBARS in Equine Osteoarthritis
Source: Vet Sci. 2026 Jan 31;13(2):140. doi: 10.3390/vetsci13020140 (PMC12945042; doi:10.3390/vetsci13020140)

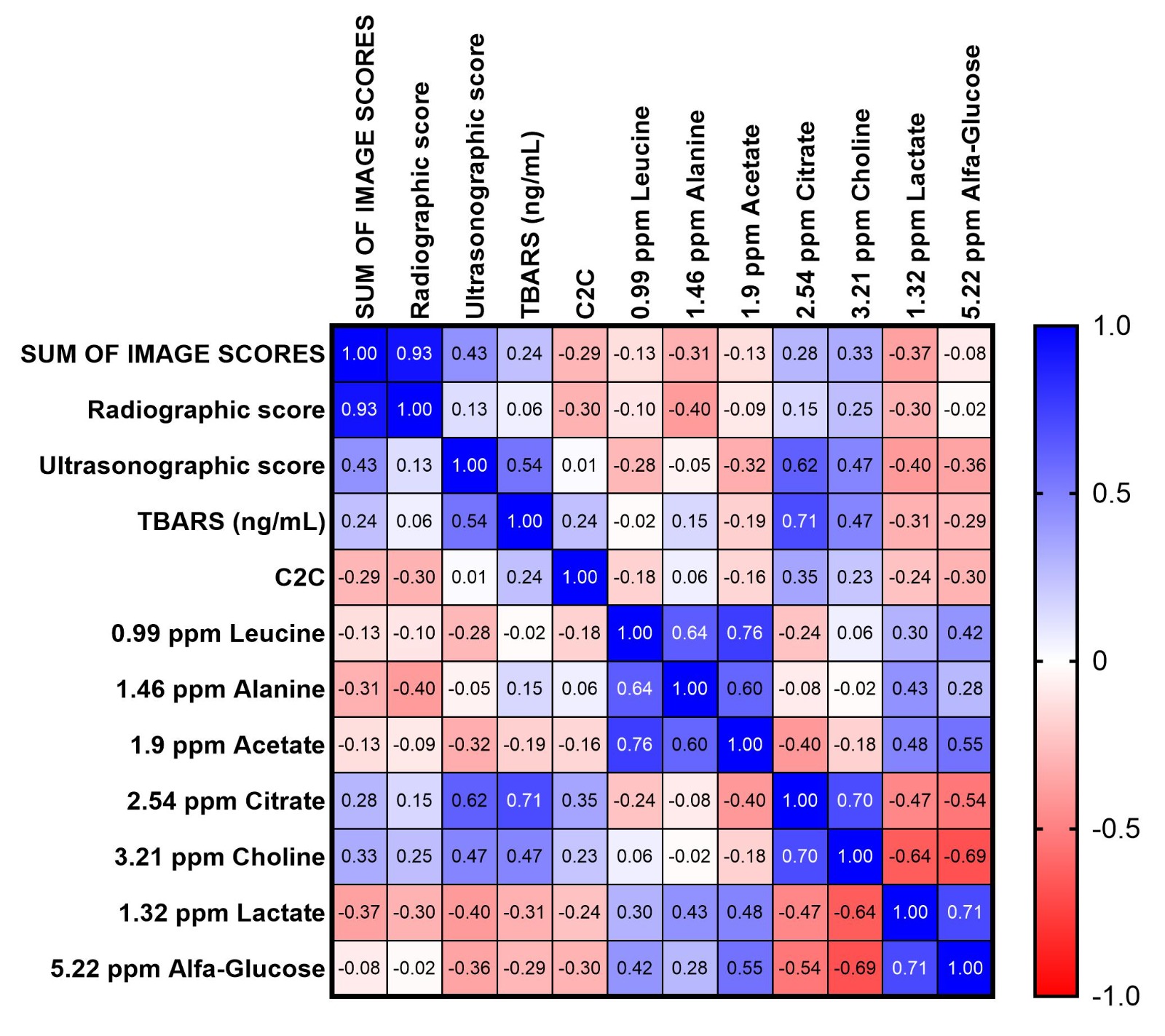

Supplement: Supplementary file 1 [file vetsci-13-00140-s001.zip › 2025 Supplementary files/2025 12 Spearmans' correlation test (heatmap).jpeg]
